# Programmable type III-A CRISPR-Cas DNA targeting modules

**DOI:** 10.1371/journal.pone.0176221

**Published:** 2017-04-25

**Authors:** H. Travis Ichikawa, John C. Cooper, Leja Lo, Jason Potter, Rebecca M. Terns, Michael P. Terns

**Affiliations:** 1 Department of Biochemistry and Molecular Biology, University of Georgia, Athens, Georgia, United States of America; 2 Thermo Fisher Scientific, Carlsbad, California, United States of America; 3 Department of Genetics, University of Georgia, Athens, Georgia, United States of America; 4 Department of Microbiology, University of Georgia, Athens, Georgia, United States of America; Florida International University, UNITED STATES

## Abstract

The CRISPR-Cas systems provide invader defense in a wide variety of prokaryotes, as well as technologies for many powerful applications. The Type III-A or Csm CRISPR-Cas system is one of the most widely distributed across prokaryotic phyla, and cleaves targeted DNA and RNA molecules. In this work, we have constructed modules of Csm systems from 3 bacterial species and heterologously expressed the functional modules in *E*. *coli*. The modules include a Cas6 protein and a CRISPR locus for crRNA production, and Csm effector complex proteins. The expressed modules from *L*. *lactis*, *S*. *epidermidis* and *S*. *thermophilus* specifically eliminate invading plasmids recognized by the crRNAs of the systems. Characteristically, activation of plasmid targeting activity depends on transcription of the plasmid sequence recognized by the crRNA. Activity was not observed when transcription of the crRNA target sequence was blocked, or when the opposite strand or a non-target sequence was transcribed. Moreover, the Csm module can be programmed to recognize plasmids with novel target sequences by addition of appropriate crRNA coding sequences to the module. These systems provide a platform for investigation of Type III-A CRISPR-Cas systems in *E*. *coli*, and for introduction of programmable transcription-activated DNA targeting into novel organisms.

## Introduction

CRISPR-Cas systems eliminate plasmid and virus invaders in many bacteria and archaea. These systems acquire invader sequences and utilize the sequences to recognize and destroy invader nucleic acid molecules [1–6]. Invader sequences are incorporated into the CRISPR loci, which are transcribed to produce the small CRISPR (cr)RNAs that guide Cas (CRISPR-associated) protein effector complexes to destroy corresponding invader nucleic acid molecules. There are many CRISPR-Cas systems (6 types (I-VI) and 19 subtypes) that carry out these processes using distinct mechanisms, Cas proteins, and crRNA species [5, 7, 8]. The RNA-guided activities of CRISPR-Cas effector complexes are being exploited to great effect in genome editing and other applications for science, industry and medicine [9–11].

**Fig 1:**
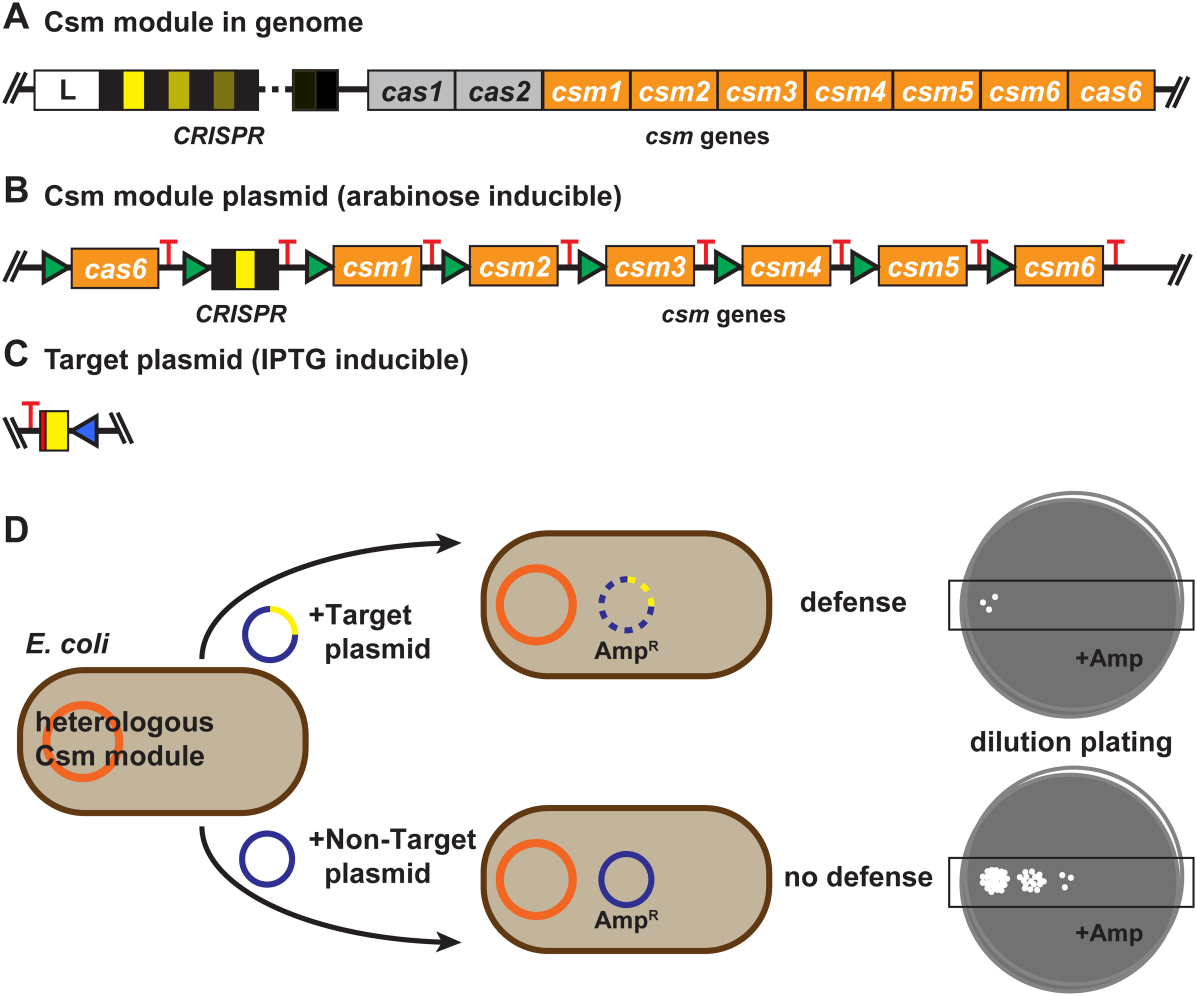
Csm module plasmids, target plasmids, and the plasmid targeting assay. (A) Representative genome organization of a Csm CRISPR-Cas module locus. The organization shown is based on *Staphylococcus epidermidis* (SEP) RP62a (GenBank NC002976). The CRISPR leader sequence (L, white box) is located upstream of the CRISPR, which can consist of various numbers of spacers (shades of yellow; 17 in SEP, 3 in STH, and 15 in LLA) bracketed by repeats (black). cas1, cas2, cas6, and csm1-csm6 genes are found in proximity to the CRISPR. The relative organization of the cas1, cas2 and cas6 genes is slightly different in *Streptococcus thermophilus* (STH) JIM 8232 (GenBank FR875178.1) and *Lactococcus lactis* (LLA) DGCC 7167 plasmid pKLM (GenBank JX524189.1). In addition, the STH Csm system includes a second csm6 gene (csm6-2) that was not used in this work. The LLA-associated module (found on LLA-associated plasmid pKLM) lacks a cas2 gene and includes an lch gene that shows partial homology to the relE/parE toxin gene. (B) Csm module plasmids. For each Csm system (SEP, STH and LLA), csm1-6 and cas6 sequences were codon-optimized for expression in E. coli and inserted as illustrated into a pACYC vector with chloramphenicol resistance and p15-derived origin of replication (ori). A T7 promoter (green) was engineered upstream of the csm and cas6 genes and CRISPR, providing increased expression in the presence of arabinose in BL21AI. A T7 terminator (or variant; red) was engineered downstream of each element. (C) Target plasmids. A target sequence comprised of the spacer of the corresponding Csm system (yellow) was inserted into a pTrcHis-TOPO plasmid with ampicillin resistance and pBR322-derived ori. The Trc promoter element (blue) allows IPTG-inducible increase in expression of a target RNA complementary to the crRNA of the Csm system. The sequence flanking the target (red) was designed so that the sequence of the DNA target strand and the target RNA was identical to, and would not interact with, the crRNA 5’ tag. (D) Plasmid targeting assay. *E*. *coli* BL21AI strains containing the Csm module plasmid (orange; chloramphenicol selectable) are transformed with target or non-target plasmids that confer ampicillin resistance (blue; ampicillin selectable). Serial dilutions of transformed cells are spotted onto plates containing chloramphenicol and ampicillin. Plasmid targeting (defense) results in a reduction in colonies able to grow in the presence of ampicillin.

Type III-A or Csm CRISPR-Cas systems typically include *cas1* and *cas2* genes that function in invader sequence acquisition, a *cas6* gene that functions in crRNA production, and six subtype specific genes, *csm1*-*csm6*, as well as a CRISPR locus that encodes the crRNAs (see Fig 1A). Like the related Type III-B Cmr complex [12], the Csm CRISPR-Cas effector complex is stimulated to cleave DNA by interaction with a target RNA that is complementary to the crRNA of the effector complex. Thus, these Type III defense systems depend on directional transcription of the target sequence of the invader for invader resistance [13–16]. The Csm (and Cmr) complex also cleaves the target RNA, [14, 16–18] which provides protection against RNA viruses [18]. The Csm1 (Cas10) protein provides DNA cleavage activity and Csm3 provides RNA cleavage activity [19–22]. Much remains to be learned about these intricate, powerful complexes that target both DNA and RNA.

In this work, we have developed self-contained DNA targeting modules comprised of Type III-A Csm CRISPR-Cas systems from 3 organisms: *Staphylococcus epidermidis* [15], *Streptococcus thermophilus* [16] and *Lactococcus lactis* [23]. When expressed in *E*. *coli*, the Csm modules confer specific resistance to plasmids that express transcripts containing crRNA-targeted sequences. The system is programmable for novel targets via simple introduction of appropriate sequences in the CRISPR of the module and adaptable for use in a wide range of organisms.

## Materials and methods

### Csm module plasmids

*csm1-csm6* and *cas6* genes were identified in each *L*. *lactis* plasmid pKLM, *S*. *epidermidis*, and *S*. *thermophilus* JIM 8232 and codon optimized for expression in *E*. *coli* (Figures A-C in S1 File). Internal restriction sites were minimized. Each gene and the CRISPR had an independent T7 promoter and high expression RBS as well as a T7 terminator (or variant) followed by a random 20 bp buffer sequence. Restriction sites were added to allow for modification in the final vector. The CRISPR contained BbsI sites flanking repeat sequences to allow for Golden Gate cloning of a spacer sequence and flanking repeat sequence as needed. The full module was designed as shown in Fig 1B and divided into ~1000bp fragments with Golden Gate (BbsI) compatible ends. The fragments were synthesized by GeneArt (ThermoFisher Scientific) then assembled by Golden Gate into a minimized pACYC vector. The p15a origin and the tetracycline and chloramphenicol resistance marker components from pACYC184 were synthesized eliminating any unneeded sequences and restriction sites to generate the minimized pACYC. The plasmids were transformed into a BL21 strain with an arabinose inducible T7 RNA polymerase gene (BL21-AI).

### Target plasmids

To generate target plasmids for each module, a target region containing sequence complementary to a specified crRNA (encoded by the CRISPR spacer on the Csm module plasmid) plus an 8-nt sequence designed not to interact with the crRNA 5’ tag (identical to the 8-nt crRNA tag) was synthesized and cloned into the vector using DNA Topoisomerase I between the Trc promoter element (IPTG inducible) and rrnB T1/T2 terminator of the pTrcHis-TOPO plasmid, which carries the colE1 origin of replication and ampicillin resistance marker (ThermoFisher Scientific) (Figures D-H in S1 File). For the LLA system, we also generated plasmids with the target region in reverse orientation relative to the Trc promoter (LLA-r), and lacking a promoter and flanked by terminators (CAGAAAGTCAAAAGCCTCCGACCGGAGGCTTTTGACTTGAT and ATCCGGCAAACAAACCACCGCTGGTAGCGGTGGTTTTTTTGTTTG; t-LLA-f-t). A control plasmid not targeted by the CRISPR-Cas system (HET-f) included the target region sequence: CTGAAGTGCTCTCAGCCGCAAGGACCGCATACTACAA. The pTrcHis-TOPO plasmid was circularized and confirmed by sequencing to obtain the no target control plasmid.

### Plasmid interference assay

BL21-AI (Thermo Fisher Scientific) was transformed with a Csm module plasmid to generate a host strain. Fifty μl of electrocompetent host strain cells were transformed with 100 ng of target plasmid by electroporation using Gene Pulser II Electroporation System (Bio-Rad). Transformants were immediately transferred to 1 ml of SOC medium and shaken at 200 rpm in a shaker incubator at 37°C. After one hour of shaking, a series of 10-fold dilutions was made in LB medium prior to plating either 5 or 10 μl each on grid lined LB-agar plates containing antibiotic with or without 0.2% arabinose or 1mM IPTG inducer as indicated. LB-agar plates were incubated overnight at 37°C and emerging colonies were documented under white light in Gel Doc XR (Bio-Rad).

### Plasmid detection assay

To assess the physical presence of target plasmids, 10mL LB medium containing chloramphenicol (34 μg/ml) with or without 1mM IPTG was inoculated with single colonies isolated from a LB-agar plate containing chloramphenicol (34 μg/ml) and ampicillin (50 μg/ml). Cultures were shaken at 200 rpm at 37°C overnight. Cells were collected and plasmids were purified by PureLink Quick Plasmid Miniprep Kit (Invitrogen). Csm module plasmid and target plasmid were linearized by NheI (New England Biolabs), 100 ng total DNA was analyzed by agarose gel electrophoresis. The gel was stained with ethidium bromide (0.5 μg/ml) and documented under UV light in Gel Doc XR (Bio-Rad).

## Results and discussion

### Csm modules from STH, SEP and LLA defend against plasmid invaders in *E*. *coli*

Systems for heterologous expression of CRISPR-Cas systems provide the potential to confer invader protection and other potential functions to desired organisms. A functional Type II-A (Cas9-based) system derived from *Streptococcus thermophilus* genes was the first functional CRISPR-Cas system to be heterologously expressed in *E*. *coli* [24]. To test the functionality and portability of various Type III-A Csm defense systems, we generated Csm module expression plasmids and tested them for the ability to eliminate potential target plasmids when expressed in *E*. *coli* (BL21AI, which lacks *cas* genes [25, 26]. Type III-A Csm systems are found in the genomes of *Staphylococcus epidermidis* RP62a (SEP: GenBank NC002976) and *Streptococcus thermophilus* JIM 8232 (STH: GenBank FR875178.1) and on the *Lactococcus lactis*-associated plasmid pKLM (LLA; pKLM: GenBank JX524189.1 [23]). Csm modules typically include Cas1 and Cas2 proteins for adaptation, a CRISPR locus and Cas6 protein for crRNA production, and six Csm proteins for invader destruction. The organization of the Csm module genes found in SEP is shown in Fig 1A.

To generate Csm defense module expression plasmids for each the STH, SEP and LLA system, codon optimized *csm1-csm6* and *cas6* genes and a minimal CRISPR array were each cloned with a T7 promoter and terminator as shown in Fig 1B. (Note: the Csm module in STH JIM 8232 includes a second *csm6* gene (*csm6-2*) that was not used in this work.) The genes were cloned into a pACYC-derived minimal plasmid containing the p15a replicon and chloramphenicol resistance gene [27]. The constructs were transformed into BL21-AI *E*. *coli*, which contains a chromosomal insertion of the T7 RNA polymerase gene in the *ara*B locus of the *ara*BAD operon, allowing up-regulation of T7 RNAP (and Csm module) expression via the arabinose-inducible *ara*BAD promoter. Csm module-expressing *E*. *coli* strains were maintained on chloramphenicol. The minimal CRISPR consists of a single spacer (the first spacer of the native CRISPR locus; Fig 1B, yellow) flanked by repeat elements (Fig 1B, black).

Potential target plasmids were generated for each Csm defense system by cloning crRNA target sequences (i.e. the CRISPR spacer) into the pTrcHis-TOPO plasmid (Fig 1C, yellow). The IPTG-inducible Trc promoter element was included downstream of the spacer sequence in the target plasmid to allow expression of a target RNA complementary to the crRNA of the modular Csm system that can be increased in the presence of IPTG. The 8-nt sequence adjacent to the spacer sequence on the target plasmid was designed to avoid interaction of the DNA target strand or the target RNA with the crRNA 5’ tag (Fig 1C, red). The target plasmid encodes a gene that confers ampicillin resistance, so plasmid transformation results in growth on media containing ampicillin. Non-target control plasmids lacked the target sequence insert.

The *E*. *coli* strains expressing the three heterologous Csm modules were tested for plasmid defense by challenge with target and non-target plasmids (Fig 1D). Target and non-target plasmids were transformed into the three Csm module-expressing *E*. *coli* strains, and dilutions of the bacteria were plated on ampicillin-containing medium to assess defense against plasmid transformation.

**Fig 2:**
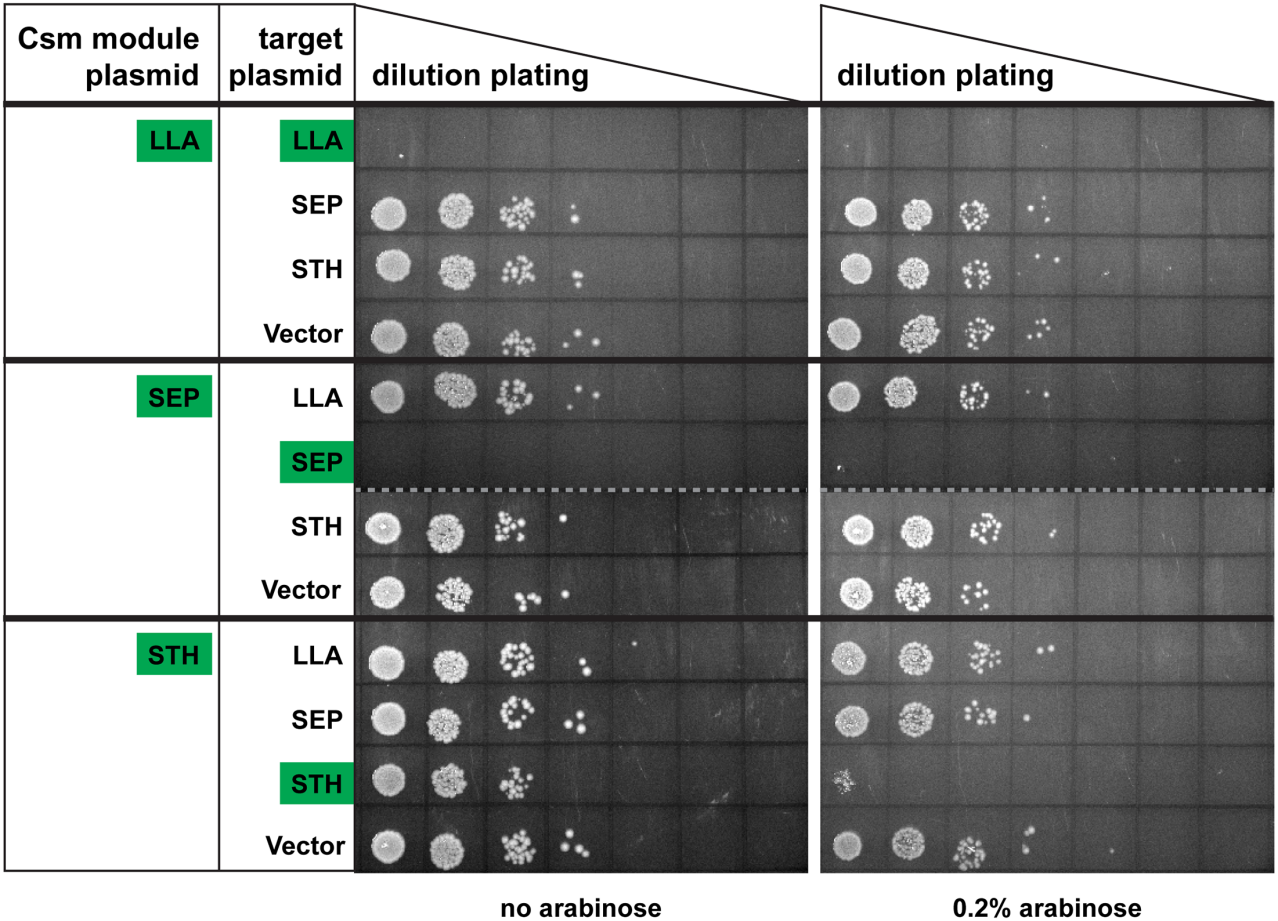
Csm modules prevent plasmid invasion in *E*. *coli*. The BL21-AI strains containing the indicated Csm module plasmids (first column; LLA, SEP or STH) were each transformed with four target plasmids (second column; LLA, SEP, STH or vector). A series of 10-fold dilutions (10^0^ to 10^6^) of transformed cells were plated on a LB agar containing 50 μg/ml ampicillin and 34 μg/ml chloramphenicol with or without 0.2% arabinose as indicated. Representative results of 3 experiments are shown. Corresponding Csm modules and targets are indicated with green boxes. Dashed lines separate data from different plates.

The Csm modules derived from LLA and SEP conferred specific resistance to plasmids containing target sequences, without a requirement for induction of either module gene or target RNA expression (with arabinose or IPTG, respectively) above baseline levels. As shown in Fig 2, plasmids lacking an LLA crRNA target sequence (containing no inserted sequence (vector) or containing a non-target sequence (i.e. target sequence for crRNAs from the SEP or STH Csm system module (SEP, STH))) successfully transformed the *E*. *coli* strain expressing the LLA Csm module, producing significant numbers of ampicillin-resistant colonies at the plated dilutions (Fig 2, top row, LLA Csm module plasmid). However, the LLA target plasmid (containing a target sequence specific for the LLA Csm system crRNA (LLA)) failed to produce ampicillin-resistant colonies at the same dilutions in the *E*. *coli* strain with the LLA Csm module expression plasmid (Fig 2, top row, LLA Csm module plasmid). Similarly, the *E*. *coli* strain with the SEP Csm module expression plasmid was transformed by plasmids lacking target sequences (no inserted sequence or unrecognized inserted sequence) but was resistant to plasmids containing the target sequence for the respective Csm module (Fig 2, middle row). The experiments were done in the absence of IPTG. The LLA and SEP modules also did not require arabinose induction; plasmid resistance was observed in the absence of arabinose, suggesting sufficient expression of module components in the absence of induction (Fig 2, top and middle rows, no arabinose). The STH module required arabinose induction of Csm module expression for substantial reduction in invader-induced colony formation (Fig 2, bottom row, 0.2% arabinose). The results shown here demonstrate the functionality of three distinct Type III-A Csm CRISPR-Cas systems, comprised of a minimal CRISPR and Cas6 for crRNA production and Csm1-Csm6, to confer specific invader defense to *E*. *coli*.

### Target sequence transcription stimulates plasmid elimination

**Fig 3:**
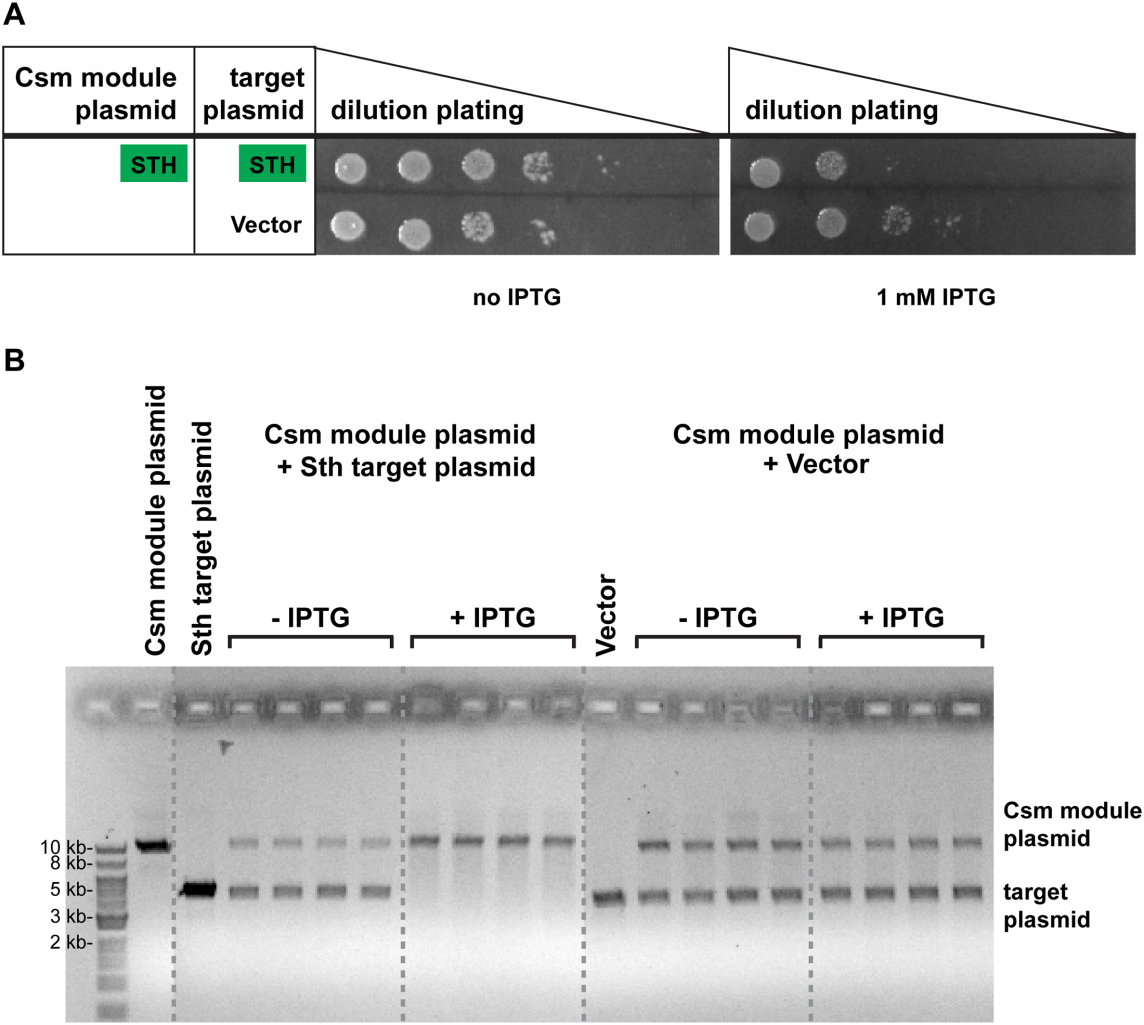
Transcription-dependent loss of the target plasmid. BL21-AI strain with the STH Csm module plasmid was challenged with plasmids with STH target sequence or without a target sequence with or without IPTG (1μM) as indicated. (A) Plasmid targeting assay was performed as in Fig 2 (without arabinose). Corresponding Csm modules and targets are indicated with green boxes. (B) Presence of plasmids was assessed by plasmid extraction following overnight growth in medium with or without 1mM IPTG as indicated. Plasmid DNA was linearized, separated by agarose gel electrophoresis and visualized with ethidium bromide. Plasmid DNA from 4 individual colonies is shown for each condition. Positions of linearized Csm module and target plasmids are indicated. Dashed lines indicate rearranged non-contiguous sections of single original gel.

To determine whether plasmid defense is stimulated by transcription of the target sequence found on the plasmid, we examined plasmid transformation in the presence of IPTG. The target plasmids tested in Fig 2 include IPTG-inducible promoters downstream of the target sequence to increase expression from the Trc promoter (Fig 1C). The LLA and SEP systems eliminate plasmids very efficiently at the dilutions examined, even in the absence of IPTG induction of target transcription (Fig 2). To examine the potential effect of increased target transcription, the *E*. *coli* strain expressing baseline levels of the STH Csm module (in the absence of arabinose) was challenged with plasmids in the presence (and absence) of IPTG. While increased plasmid transcription had little or no effect on resistance against the plasmid lacking target sequence (Fig 3A, vector), resistance against the target plasmid was increased in the presence of IPTG (Fig 3A, STH). The results indicate that transcription of the target sequence stimulates plasmid defense by the STH Type III-A Csm module expressed in *E*. *coli*.

To assess whether the observed growth defect was due to transcription-dependent, Csm crRNP-mediated plasmid degradation, we also directly examined the presence of the target and non-target plasmids in the transformed *E*. *coli* strains expressing the STH Csm module by plasmid extraction and agarose gel electrophoresis. Plasmids were extracted from strains transformed with both the STH target plasmid (Fig 3B, left panels) and the no target plasmid (Fig 3B, right panels) and assessed by agarose gel electrophoresis and DNA staining. Both the Csm module plasmid and the target plasmid can be observed in the strain extracts in the absence of IPTG induction of target sequence transcription (-IPTG). Induction of transcription resulted in loss of the target plasmid containing the STH target sequence, but not the plasmid lacking the target sequence (Fig 3B, +IPTG). The results indicate that the STH Type III-A Csm module expressed in *E*. *coli* specifically eliminates the target plasmid and that elimination depends on transcription of the target sequence.

### Plasmid resistance depends on directional transcription of the target sequence

**Fig 4:**
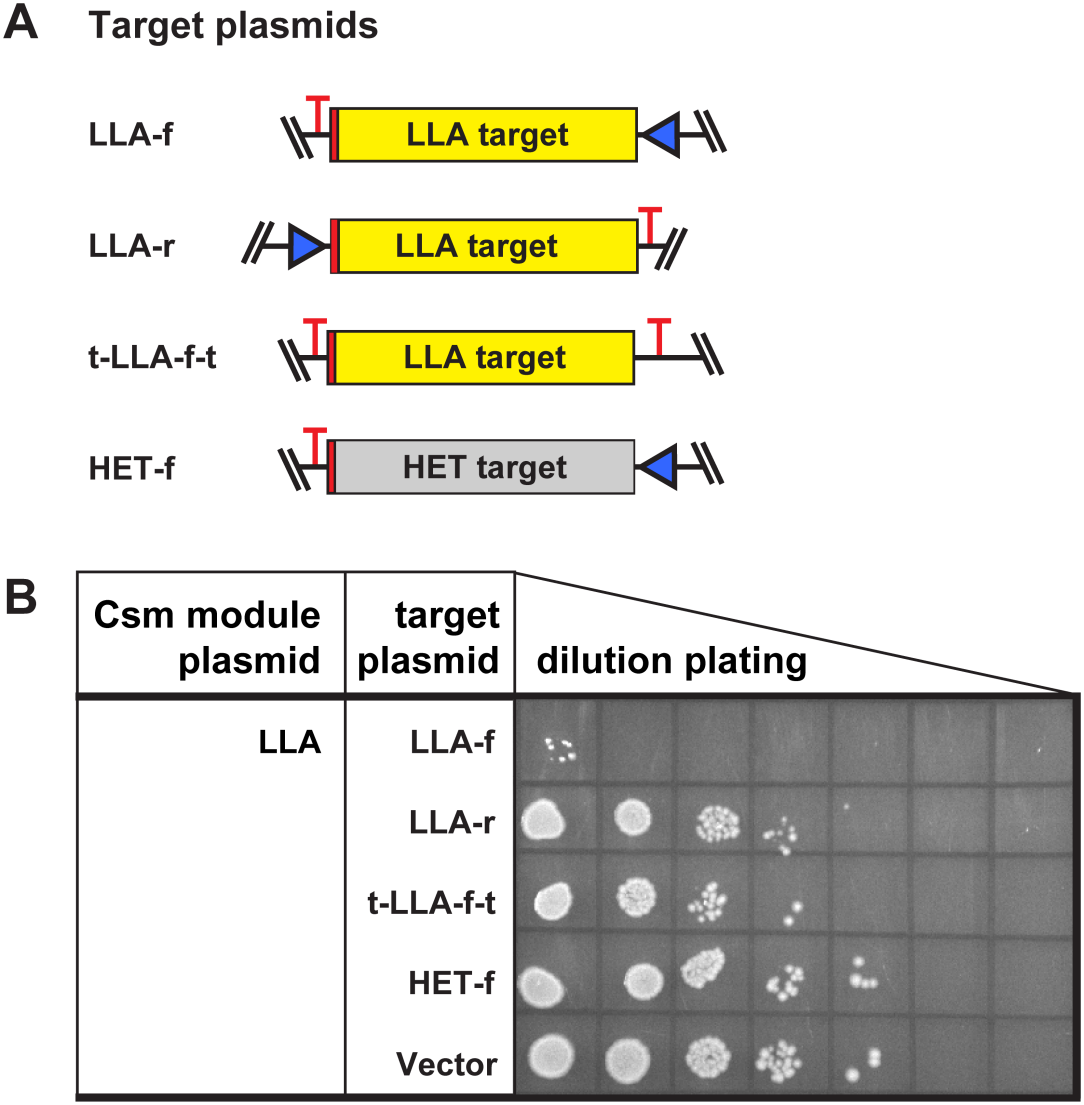
Directional transcription is required for plasmid interference. The BL21AI strain containing the LLA Csm module plasmid was challenged with five different target plasmids. (A) The target plasmids contain the LLA target sequence (yellow), a heterologous sequence (grey) or no target sequence (not shown in panel A). The LLA target (yellow) was oriented relative to the Trc promoter (blue) to effect transcription of either an RNA complementary to the crRNA (LLA-f) or the reverse complement (LLA-r). Transcription of the LLA target sequence was inhibited by flanking transcription terminators (red T) in the t-LLA-f-t target plasmid. (B) Plasmid targeting assay was performed as in Fig 2. Representative results of 3 experiments are shown.

In order to determine whether transcription of the target region from either direction stimulates plasmid targeting, we examined the ability of the *E*. *coli* strain expressing the LLA Csm module to respond to a series of target plasmids (Fig 4A). The originally tested target plasmid includes a promoter for transcription of a target RNA complementary to the crRNA (Figs 1C and 4A, LLA-f). Additional target plasmids were designed with promoters and terminators for: transcription of the reverse complement of the target RNA (RNA not complementary to the crRNA; Fig 4A, LLA-r), minimal transcription of the target region (target sequence flanked by terminators; Fig 4A, t-LLA-f-t), or transcription of a heterologous non-target RNA (Fig 4A, HET-f). The plasmid transcribing the target RNA was successfully targeted by the Csm module as indicated by the reduction in ampicillin-resistant colony formation relative to the plasmid lacking target sequence (vector), containing a minimally transcribed target sequence (t-LLA-f-t) or transcribing a heterologous sequence (HET-f; Fig 4B). However, the plasmid transcribing the reverse complement of the target RNA (LLA-r) was not targeted significantly relative to the controls (Fig 4B).

The results of these experiments demonstrate that activation of plasmid targeting by the LLA Type III-A Csm module depends on directional transcription of the target sequence. Moreover, the plasmids successfully targeted by all three Csm modules in our experiments include a promoter downstream of the target sequence that generates a crRNA-complementary target RNA (Figs 2–4). Our findings indicate that transcription of the strand of the target region that generates a crRNA-complementary RNA activates plasmid targeting by these Type III-A Csm CRISPR-Cas systems. Transcription of a target region in one direction but not the other was previously found to be effective in activating DNA targeting for the SEP system expressed in *E*. *coli*, and initially ascribed to the inability of crRNAs complementary to the coding strand of the DNA to activate defense [15]. However, target RNA activation of ssDNA cleavage has more recently been directly demonstrated for the reconstituted Type III-B Cmr effector complexes from *Pyrococcus furiosus* [28] and *Thermatoga maritima* [29] and Type III-A Csm system from the STH DGCC8004 strain [16].

### The Csm module can be programmed to target novel heterologous sequences

**Fig 5:**
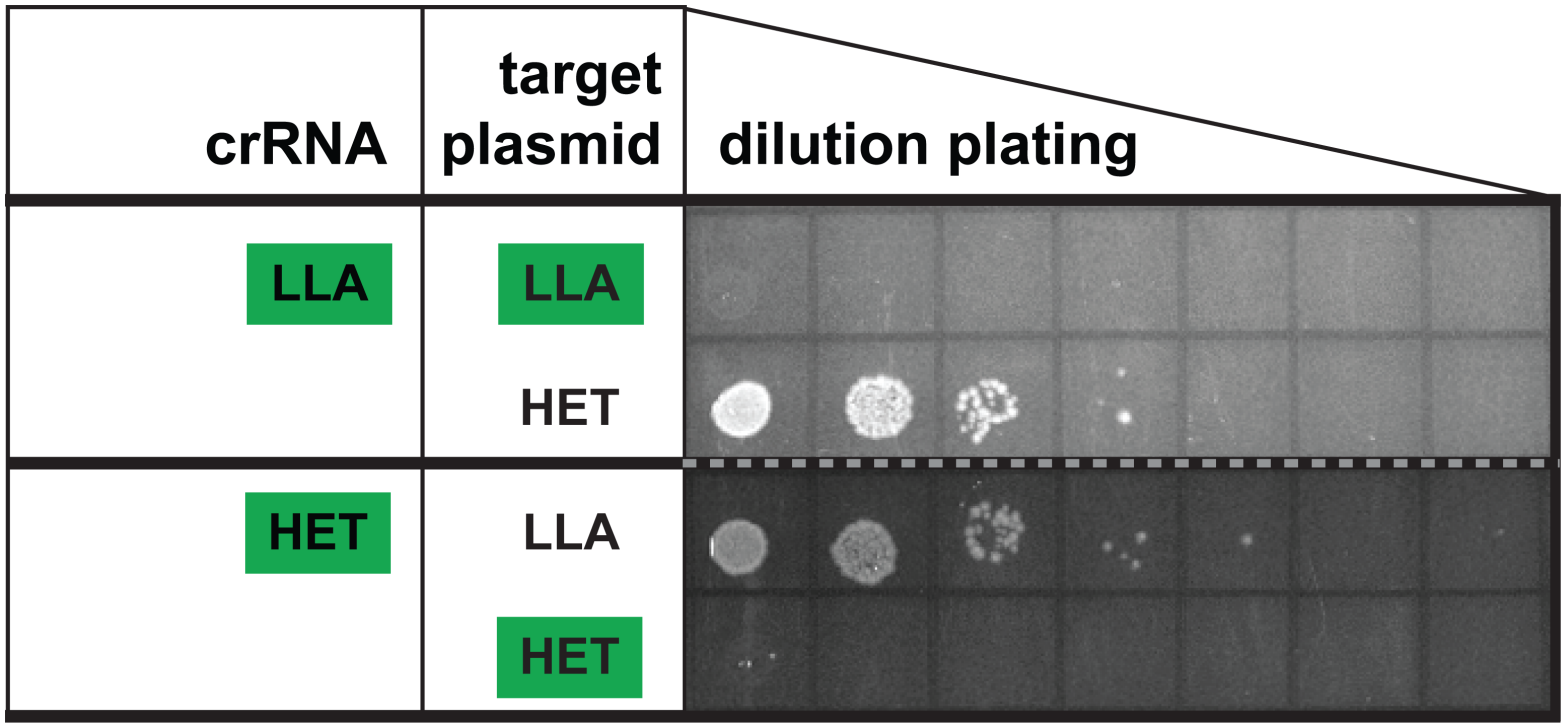
crRNA engineering to eliminate novel targets. BL21AI strains containing the LLA Csm module plasmid encoding either the LLA crRNA or a novel crRNA targeting the heterologous target sequence (first column; LLA or HET) were challenged with LLA or HET target plasmids as indicated (second column; LLA or HET). Plasmid targeting assay was performed as in Fig 2. Representative results of 3 experiments are shown. Corresponding Csm modules and targets are indicated with green boxes.

To test for the ability of the system encoded on the Csm module plasmid to be programmed to recognize and eliminate novel targets, we modified the CRISPR of the Csm module to encode a crRNA targeting a new sequence—the heterologous control sequence (HET). As we have observed (Fig 4), *E*. *coli* expressing the LLA module with a native encoded crRNA successfully eliminated a plasmid containing the LLA target sequence but not a plasmid containing the heterologous target sequence (Fig 5, LLA crRNA). Notably, the *E*. *coli* strain expressing the LLA module encoding the new heterologous crRNA successfully eliminated the plasmid containing the HET target sequence but not a plasmid containing LLA sequence (Fig 5, HET crRNA). (All target plasmids included promoters for transcription of the activating target RNA.) Our results indicate that plasmid-encoded Type III-A Csm modules can be engineered to target novel invaders in heterologous systems. Moreover, the ability of Csm modules from three distinct organisms to function in the heterologous *E*. *coli* host, indicates that the Csm systems do not require additional genes that are present in their native hosts and absent in *E*. *coli*.

The series of modules of Type III-A CRISPR-Cas systems developed here provide a platform for facile investigation of Type III-A CRISPR-Cas systems in *E*. *coli*. Moreover, these studies establish tools for the introduction of sophisticated programmable transcription-activated DNA targeting into novel organisms. In addition to invader elimination, the Type III-A Csm and Type III-B Cmr systems provide attractive platforms for functions triggered by detection of specific RNAs within cells.

## Supporting information

S1 File Sequences of the plasmids used in this study. (DOCX)
