# Supplementary material for: Programmable type III-A CRISPR-Cas DNA targeting modules
Source: PLoS One. 2017 Apr 25;12(4):e0176221. doi: 10.1371/journal.pone.0176221 (PMC5404769; doi:10.1371/journal.pone.0176221)
Supplement: S1 File — (DOCX) [file pone.0176221.s001.docx]

**Supporting information**

**Figure A in S1 file. *Lactococcus lactis* (LLA) Csm module.** LLA system genes *csm1-6* and cas6 (orange arrows) cloned into a pACYC-based vector, along with minimal a CRISPR spacer (yellow), flanked by repeat sequences (black). Green arrows and red rectangles indicate transcriptional promoters or terminators, respectively. Chloramphenicol resistance cassette (CmR) and p15A origin of replication region (p15A) are indicated with blue or pink arrows, respectively. Plasmid map illustration made using the SnapGene program. Plasmid sequence information is in GenBank format, using ApE program.


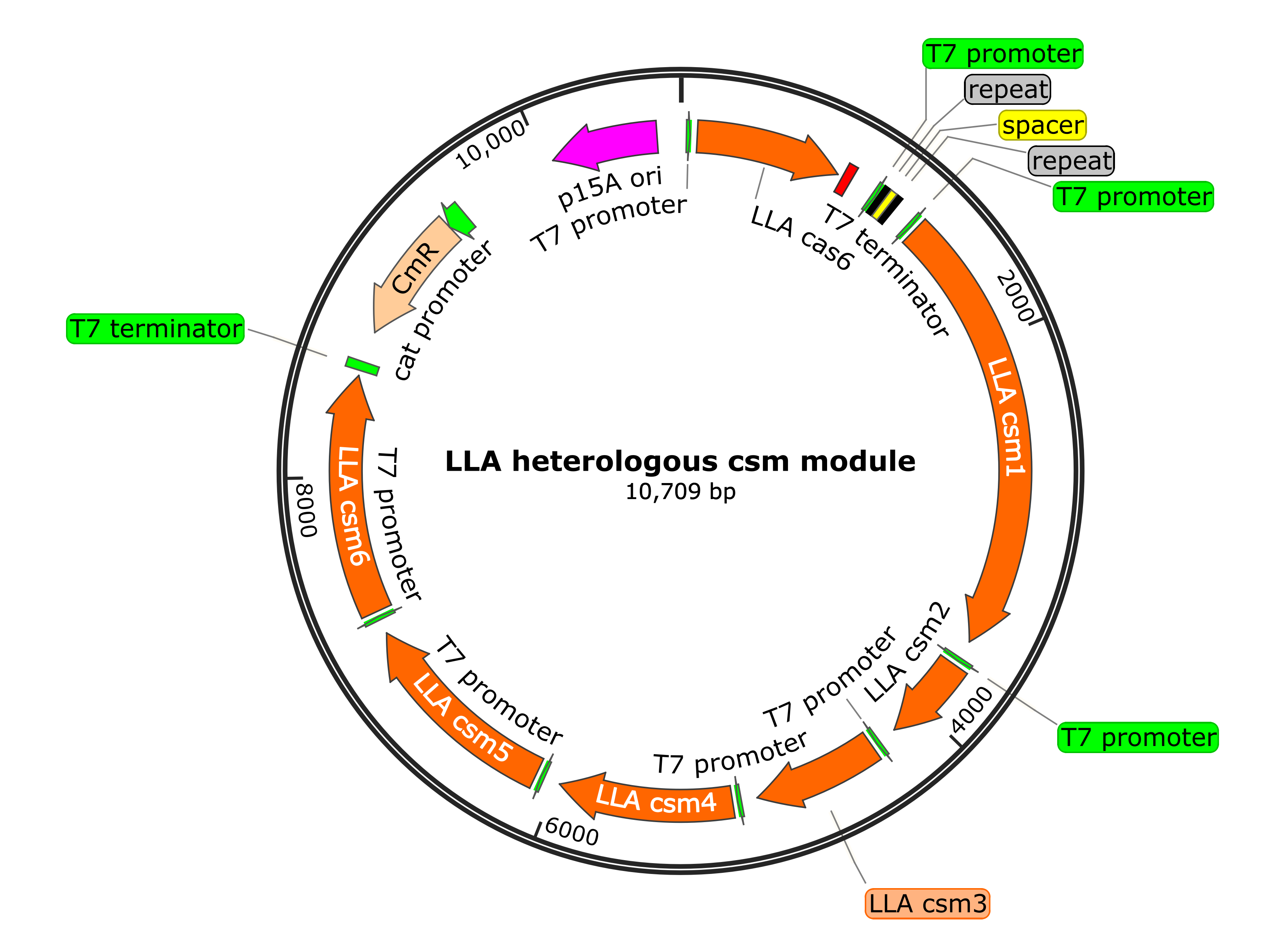


LOCUS LLA_heterologous 10709 bp ds-DNA circular 19-FEB-2017

DEFINITION synthetic circular DNA

ACCESSION .

VERSION .

KEYWORDS LLA all in one pACYC

SOURCE synthetic DNA construct

ORGANISM synthetic DNA construct

REFERENCE 1 (bases 1 to 10709)

AUTHORS .

TITLE Direct Submission

JOURNAL Exported Saturday, Feb 18, 2017 from SnapGene 3.3.3

http://www.snapgene.com

COMMENT

COMMENT ApEinfo:methylated:1

FEATURES Location/Qualifiers

promoter 31..49

/note="T7 promoter"

/note="promoter for bacteriophage T7 RNA polymerase"

/label=T7 promoter

/ApEinfo_fwdcolor=#00ff00

/ApEinfo_revcolor=pink

/ApEinfo_graphicformat=arrow_data {{0 1 2 0 0 -1} {} 0}

width 5 offset 0

CDS 86..832

/codon_start=1

/note="LLA Cas6"

/translation="MIVKLRYKINLPNSLRTQNIGSTLHGVLMELLPSELVEHLHNLSYNPFRQRLIFEKELVIWEIVGLHKMVSEELLKLENLREITIKRAQKTVSLSLLSKDAIAVDDLVKKEMGREIDSRIISLKFTSPTSFKANGHYDIFPDIRKIFRSLMMNFDFFSETTKIYDYEVLSYIEENVHIVSYKLMTKNFHLEKIKVKGFQGDMTLKVTGAEQFVKLVLLMIKYATFAGIGMKTSLGMGGVSINERHYLR"

/label=LLA cas6

/ApEinfo_fwdcolor=#ff8000

/ApEinfo_revcolor=pink

/ApEinfo_graphicformat=arrow_data {{0 1 2 0 0 -1} {} 0}

width 5 offset 0

terminator 858..905

/note="T7 terminator"

/note="transcription terminator for bacteriophage T7 RNA

polymerase"

/label=T7 terminator

/ApEinfo_fwdcolor=#ff0000

/ApEinfo_revcolor=pink

/ApEinfo_graphicformat=arrow_data {{0 1 2 0 0 -1} {} 0}

width 5 offset 0

promoter 1033..1051

/note="T7 promoter"

/note="promoter for bacteriophage T7 RNA polymerase"

/label=T7 promoter(1)

/ApEinfo_label=T7 promoter

/ApEinfo_fwdcolor=#00ff00

/ApEinfo_revcolor=pink

/ApEinfo_graphicformat=arrow_data {{0 1 2 0 0 -1} {} 0}

width 5 offset 0

misc_feature 1062..1097

/note="repeat"

/label=repeat

/ApEinfo_fwdcolor=#000000

/ApEinfo_revcolor=pink

/ApEinfo_graphicformat=arrow_data {{0 1 2 0 0 -1} {} 0}

width 5 offset 0

misc_feature 1098..1132

/note="spacer"

/label=spacer

/ApEinfo_fwdcolor=#ffff00

/ApEinfo_revcolor=pink

/ApEinfo_graphicformat=arrow_data {{0 1 2 0 0 -1} {} 0}

width 5 offset 0

misc_feature 1133..1168

/note="repeat"

/label=repeat(1)

/ApEinfo_label=repeat

/ApEinfo_fwdcolor=#000000

/ApEinfo_revcolor=pink

/ApEinfo_graphicformat=arrow_data {{0 1 2 0 0 -1} {} 0}

width 5 offset 0

promoter 1270..1288

/note="T7 promoter"

/note="promoter for bacteriophage T7 RNA polymerase"

/label=T7 promoter(2)

/ApEinfo_label=T7 promoter

/ApEinfo_fwdcolor=#00ff00

/ApEinfo_revcolor=pink

/ApEinfo_graphicformat=arrow_data {{0 1 2 0 0 -1} {} 0}

width 5 offset 0

CDS 1315..3588

/codon_start=1

/note="LLA Csm1"

/translation="MDKINLVCGSLLHDIGKIIYRGTSERAKHSKLGGDFIKSFEQFRNTELTDCIRYHHAQEITSVKSNKEKNSLFYITYIADNISSGMDRRKDLEEGAEGFNWDKKVALGSVFNVLNEKEKGRQNYSYPFVARTRIKEEPLNFPTATQNQYTTSYYDGLITDMKTILQRLKPDKEHINSLLQMMESLWSYVPSSTDKNQLVDISLYDHSRTTAAIASAIYDYFQAENITDYQKELFDYNATEFYDKNAFLMMNFDMSGVQNFIYNISGSKALKSLRARSFYLDMLLEYISDNLLEKLELSRANILYVGGGHAYLLLANTNKTKAILSDFEHDLKTWFLDKFKIDLYVAMAYTEVSANDLMNHNGHYRDIYRRLSQKTSAKKANRYTAEEILNLNHQGTENARECRECKRSDLLIEEDDICEICDSLQKVSRDLTRENIFVIANEGVLDMPFGKKMSALSYSQADKLKKSNAEVQIYAKNISEIGQNLMTRIDMGDYTYRSDFHEMLEEVEVGINRLGVLRADVDNLGQAFINGIPDDYLSISRTATFSRAMSRFFKNYLNQLLAEKSYKINVIYAGGDDLFMIGAWQDILDFSIVLKQKFADFTQNKLSISAGIGMFREKYPVARMASLTGDLEDAAKDYKPDERAVQATKNAVTLFDATNVFSWDTLENDIFVKLDAITKNFEKLDETGKAFIYRLIDLLRGVNENQQINIARLAYTLSRMEEKIGKTFAQELYNWANADRKTLIMALEIYILKTRER"

/label=LLA csm1

/ApEinfo_fwdcolor=#ff8000

/ApEinfo_revcolor=pink

/ApEinfo_graphicformat=arrow_data {{0 1 2 0 0 -1} {} 0}

width 5 offset 0

promoter 3682..3700

/note="T7 promoter"

/note="promoter for bacteriophage T7 RNA polymerase"

/label=T7 promoter(3)

/ApEinfo_label=T7 promoter

/ApEinfo_fwdcolor=#00ff00

/ApEinfo_revcolor=pink

/ApEinfo_graphicformat=arrow_data {{0 1 2 0 0 -1} {} 0}

width 5 offset 0

CDS 3727..4179

/codon_start=1

/note="LLA Csm2"

/translation="MGHHHHHHSGGTELKIGNEKVNSTNFGDFAEKAIRGINHKPFVNSKGGEQKITTSKIRGILELVNKVYNRVINTNDVELSENILADIAYIKVKIAYESGREPVVKDFIQRTAFTAAITDVMNQRTRESFLLFARYVESLIAYFKFYGGKD"

/label=LLA csm2

/ApEinfo_fwdcolor=#ff8000

/ApEinfo_revcolor=pink

/ApEinfo_graphicformat=arrow_data {{0 1 2 0 0 -1} {} 0}

width 5 offset 0

promoter 4273..4291

/note="T7 promoter"

/note="promoter for bacteriophage T7 RNA polymerase"

/label=T7 promoter(4)

/ApEinfo_label=T7 promoter

/ApEinfo_fwdcolor=#00ff00

/ApEinfo_revcolor=pink

/ApEinfo_graphicformat=arrow_data {{0 1 2 0 0 -1} {} 0}

width 5 offset 0

CDS 4318..4962

/codon_start=1

/note="LLA Csm3"

/translation="MKLVIEGTIVLKTGMHIGGSSDFSAIGAVDSPVVRDTLTRLPLIPGSSLKGKMRYLLAKELNNGILLNEPNNDQDEILRLFGSSEKDKIRRARLKFNDIKLSNLAELETFNVSSTEVKFENTINRKTAVANPRQIERVIAGSKFDFEIFYNLDDIKEVEKDFENIKQGFDLLEFDYLGGHGTRGSGRIAFENLSVITAVGNFEKINTLNEILGA"

/label=LLA csm3

/ApEinfo_fwdcolor=#ff8000

/ApEinfo_revcolor=pink

/ApEinfo_graphicformat=arrow_data {{0 1 2 0 0 -1} {} 0}

width 5 offset 0

promoter 5045..5063

/note="T7 promoter"

/note="promoter for bacteriophage T7 RNA polymerase"

/label=T7 promoter(5)

/ApEinfo_label=T7 promoter

/ApEinfo_fwdcolor=#00ff00

/ApEinfo_revcolor=pink

/ApEinfo_graphicformat=arrow_data {{0 1 2 0 0 -1} {} 0}

width 5 offset 0

CDS 5090..5983

/codon_start=1

/note="LLA Csm4"

/translation="MKIIKLYFESPVHFGEKRLSESKITFSADTLFSALMIEAVGLGKEDEFYQLASNNLVKFSDAFPFIDQYYYIPKPMFNLKLEKEDENPSKAFKKLLYVPIDSLEDYLSGGLDAYFERESFNLGKLALSEKVQQHDFKDSEPYNVGTFTFKENTGLYVLIEQTHPLLEELLENLQYSGIGGKRNSGYGKFKFEILEDSDIEDLFSAKGNRKILLSGALPKDAELEQALKNASYLLERRGGFVQSDTYATNLVKKQDLYVFKSGSTFENSFDGDIYQVGKKGNHPVYKYAKSFFLEVSV"

/label=LLA csm4

/ApEinfo_fwdcolor=#ff8000

/ApEinfo_revcolor=pink

/ApEinfo_graphicformat=arrow_data {{0 1 2 0 0 -1} {} 0}

width 5 offset 0

promoter 6067..6085

/note="T7 promoter"

/note="promoter for bacteriophage T7 RNA polymerase"

/label=T7 promoter(6)

/ApEinfo_label=T7 promoter

/ApEinfo_fwdcolor=#00ff00

/ApEinfo_revcolor=pink

/ApEinfo_graphicformat=arrow_data {{0 1 2 0 0 -1} {} 0}

width 5 offset 0

CDS 6112..7173

/codon_start=1

/note="LLA Csm5"

/translation="MKKTYRVTLTALGPIFIGGGEKLKKYEYIFDKQKKVAHMIDHTKFTKYLLEKNLLDDFTSRVNSHFDLYDYLVNKKGIVFMPLVKYSVPVAQFRTEVKNRFGKPISSPPMNDLNTFVKDAFGRPYIPGSSLKGALRTAILNDLKEDTKENEVFAHLQVSDSETIDLENLKVYQKVDYSKTAKPLPLYRECLKPNTEITFTVSFDDEYLTLKKIQNALHKTYQHYYIKWLKGGKVGETLIKGVYDSHADELKKNTFALDQPSQNQGEIIYIGGGAGFVSKTLHYKSKNRDQARNDSFDILKQLFRTTYSKMRSVPDNVPVALKLAVETKTFNGRVTGKHYLEMGKARIKLEELK"

/label=LLA csm5

/ApEinfo_fwdcolor=#ff8000

/ApEinfo_revcolor=pink

/ApEinfo_graphicformat=arrow_data {{0 1 2 0 0 -1} {} 0}

width 5 offset 0

promoter 7249..7267

/note="T7 promoter"

/note="promoter for bacteriophage T7 RNA polymerase"

/label=T7 promoter(7)

/ApEinfo_label=T7 promoter

/ApEinfo_fwdcolor=#00ff00

/ApEinfo_revcolor=pink

/ApEinfo_graphicformat=arrow_data {{0 1 2 0 0 -1} {} 0}

width 5 offset 0

CDS 7294..8523

/codon_start=1

/note="LLA Csm6"

/translation="MKILISAVGDTDPIRNFHDGPLLHIVRVYRPEKIVLVHSERSLTKHDKLVKALKSIKDYSPEIIQDGVVLPDAQVAIFDEMYDTVSSIVKKYISDDEIILNISSATPQIISAMFAVNRISDFNVTAVQVKTPQHKSNEGLRHDNQEDIDKLIETNLDNQSDYENRTLADTGMKFSQDLTKRNLKALIDNYDYQGALELLKKQKSFSNIKELRKKLTEISDTIKIQGMPDKIVKSKLSNQAKSALNSYLNIDRNHKQGNIAEVLIRVKSLVEFILEDYLNNHFLDVITYKDGKPFLNASKYPEILKKFQEDAEMRGKEYHSGYLSLPAYIGILKFFEPNHDLLKHIYKIQEINQDRNKVAHSLQAFDRKNLKKVSSAVFASKQILLASFDIDNHWFSFYEDLNQEIKKLL"

/label=LLA Csm6

/ApEinfo_fwdcolor=#ff8000

/ApEinfo_revcolor=pink

/ApEinfo_graphicformat=arrow_data {{0 1 2 0 0 -1} {} 0}

width 5 offset 0

terminator 8550..8596

/note="T7 terminator"

/note="transcription terminator for bacteriophage T7 RNA

polymerase"

/label=T7 terminator(1)

/ApEinfo_label=T7 terminator

/ApEinfo_fwdcolor=#ff0000

/ApEinfo_revcolor=pink

/ApEinfo_graphicformat=arrow_data {{0 1 2 0 0 -1} {} 0}

width 5 offset 0

CDS complement(8757..9416)

/codon_start=1

/gene="cat"

/product="chloramphenicol acetyltransferase"

/note="CmR"

/note="confers resistance to chloramphenicol"

/translation="MEKKITGYTTVDISQWHRKEHFEAFQSVAQCTYNQTVQLDITAFLKTVKKNKHKFYPAFIHILARLMNAHPEFRMAMKDGELVIWDSVHPCYTVFHEQTETFSSLWSEYHDDFRQFLHIYSQDVACYGENLAYFPKGFIENMFFVSANPWVSFTSFDLNVANMDNFFAPVFTMGKYYTQGDKVLMPLAIQVHHAVCDGFHVGRMLNELQQYCDEWQGGA"

/label=chloramphenicol acetyltransferase

/ApEinfo_fwdcolor=#ffcc66

/ApEinfo_revcolor=#ffcc66

/ApEinfo_graphicformat=arrow_data {{0 1 2 0 0 -1} {} 0}

width 5 offset 0

promoter complement(9417..9519)

/note="cat promoter"

/note="promoter of the E. coli cat gene encoding

chloramphenicol acetyltransferase"

/label=cat promoter

/ApEinfo_fwdcolor=pink

/ApEinfo_revcolor=#00ff00

/ApEinfo_graphicformat=arrow_data {{0 1 2 0 0 -1} {} 0}

width 5 offset 0

rep_origin complement(10045..10590)

/direction=LEFT

/note="p15A ori"

/note="Plasmids containing the medium-copy-number p15A

origin of replication can be propagated in E. coli cells

that contain a second plasmid with the ColE1 origin."

/label=p15A ori

/ApEinfo_fwdcolor=pink

/ApEinfo_revcolor=#ff00ff

/ApEinfo_graphicformat=arrow_data {{0 1 2 0 0 -1} {} 0}

width 5 offset 0

ORIGIN

1 cttcggatcc gatagactag ccgctggtaa taatacgact cactataggg agagaattct

61 attagtactt cataaggagg acagaatgat cgtaaaactg cgatacaaga ttaacctgcc

121 gaatagcctg cgtacccaga atattggtag caccctgcat ggtgttctga tggaactgct

181 gccgagcgaa ctggttgaac atctgcataa tctgagctat aatccgtttc gtcagcgcct

241 gatttttgaa aaagaactgg tgatttggga aatcgtgggt ctgcataaaa tggttagcga

301 agaactgctg aaactggaaa atctgcgtga aattaccatt aaacgtgccc agaaaaccgt

361 tagcctgagc ctgctgagca aagatgcaat tgccgttgat gatctggtga aaaaagaaat

421 gggtcgcgaa attgatagcc gcattattag cctgaaattt accagcccga cctcctttaa

481 agcaaacggt cattatgata tcttcccgga cattcgtaaa atctttcgta gcctgatgat

541 gaacttcgac ttttttagcg aaaccaccaa aatctacgat tatgaggtgc tgagctacat

601 cgaagaaaat gttcatatcg tgagctacaa actgatgacc aaaaacttcc acctggaaaa

661 aatcaaagtg aaaggttttc agggcgacat gaccctgaaa gttaccggtg cagaacagtt

721 tgttaaactg gttctgctga tgatcaaata tgcaaccttt gcaggtattg gcatgaaaac

781 cagcctgggt atgggtggtg ttagcattaa tgaacgtcat tatctgcgct aaaagctttc

841 ctgtgagcag cgaaagccta gcataacccc ttggggcctc taaacgggtc ttgaggggtt

901 ttttgttata cgcgagataa tcacttgcat agctgcgtat ggaggaagca actcttgagt

961 gttaatatgt tgacccctgt attagggatg cgggtagtag atgtgggcag agacacccac

1021 actgccagat cttaatacga ctcactatag ggagaccatg gaaatacaac cgctcctcga

1081 taaaagggga cgagaacata cgttctttga accaagcttc aactccctcg gaaaatacaa

1141 ccgctcctcg ataaaagggg acgagaacct cgaggctgtg gtctagacat tccatacata

1201 tcgggggggt aggggttttt tgtgtgcctc tagtggctgg ctaagaaaac ttccttccag

1261 gggtatgtgt aatacgactc actataggga gaggatccat aaaggaggta aataatggac

1321 aaaattaacc tggtttgtgg tagcctgctg catgatattg gcaaaattat ctatcgtggc

1381 accagcgaac gtgcaaaaca tagcaaactg ggtggtgatt tcatcaaatc ctttgaacag

1441 tttcgcaata ccgaactgac cgattgtatt cgttatcatc atgcccaaga aatcacctcc

1501 gtgaaaagca acaaagaaaa aaacagcctg ttctatatca cctatatcgc cgataacatt

1561 agcagcggta tggatcgtcg taaagacctg gaagaaggtg cagaaggttt taactgggat

1621 aaaaaagttg cactgggcag cgtttttaat gtgctgaacg aaaaagaaaa aggtcgccag

1681 aattatagct atccgtttgt tgcacgtacc cgcattaaag aagaaccgct gaattttccg

1741 accgcaaccc agaatcagta taccaccagt tattatgatg gcctgatcac cgatatgaaa

1801 acaattctgc agcgtctgaa accggataaa gaacatatta acagtctgct gcagatgatg

1861 gaaagcctgt ggtcttatgt tccgagcagc accgataaaa atcagctggt tgatattagc

1921 ctgtatgatc attcacgtac caccgcagca attgcaagcg caatttatga ttattttcag

1981 gccgaaaaca tcaccgacta tcagaaagaa ctgttcgatt ataacgccac cgagttctat

2041 gataaaaacg cctttctgat gatgaacttc gatatgagcg gtgtgcagaa cttcatttac

2101 aatattagcg gtagcaaagc cctgaaaagc ctgcgtgccc gtagctttta tctggatatg

2161 ctgctggaat atatcagcga taatctgctg gaaaaactgg aactgagccg tgcaaatatt

2221 ctgtatgttg gtggtggtca tgcatatctg ctgctggcaa ataccaataa aaccaaagca

2281 atcctgagcg attttgagca tgatctgaaa acatggttcc tggataaatt caaaatcgac

2341 ctgtatgtgg caatggccta taccgaagtt agcgcaaatg atctgatgaa tcataacggt

2401 cattatcgcg acatttatcg tcgtctgagc cagaaaacca gcgcaaaaaa agcaaatcgt

2461 tataccgcag aagaaatcct gaatctgaat catcagggca ccgaaaatgc acgtgaatgt

2521 cgtgaatgca aacgtagcga tctgctgatt gaagaagatg atatttgcga aatttgcgat

2581 agcctgcaga aagttagccg tgatctgacc cgtgaaaaca tttttgttat tgccaatgaa

2641 ggcgttctgg acatgccgtt tggcaaaaaa atgagcgcac tgagctatag ccaggcagat

2701 aaactgaaaa aaagtaatgc cgaggtgcag atttacgcca aaaacattag cgaaattggc

2761 cagaatctga tgacccgtat tgatatgggt gattatacct atcgtagcga ttttcacgaa

2821 atgctggaag aggttgaagt gggtattaat cgtctgggtg ttctgcgtgc cgatgttgat

2881 aatctgggtc aggcctttat taacggtatt ccggatgatt atctgagcat tagccgtacc

2941 gcaaccttta gccgtgccat gagccgtttt ttcaaaaatt acctgaatca gctgctggcc

3001 gagaaaagct ataaaatcaa tgttatttat gccggtggcg acgacctgtt tatgattggt

3061 gcatggcagg atattctgga ttttagcatt gtgctgaaac agaaatttgc cgatttcacc

3121 cagaacaaac tgagcatttc agcaggtatt ggtatgttcc gcgaaaaata tccggttgcc

3181 cgtatggcaa gcctgaccgg tgatctggaa gatgcagcaa aagattataa accggatgaa

3241 cgtgcagttc aggcaaccaa aaatgcagtt accctgtttg atgccaccaa tgtttttagt

3301 tgggataccc tggaaaacga tatcttcgtt aaactggatg ccatcaccaa aaacttcgag

3361 aaactggacg aaaccggcaa agcctttatt taccgtctga ttgatctgct gcgtggggtt

3421 aatgaaaatc agcaaattaa cattgcacgc ctggcatata ccctgagccg tatggaagaa

3481 aaaatcggta aaacctttgc ccaagagctg tataattggg caaatgcaga tcgtaaaacc

3541 ctgattatgg cactggaaat ctatatcctg aaaacccgtg aacgctaaca tatggctgcg

3601 tggtcaaatg tgcgtaccct aaccccttcc ccggtcaatc ggggcggatg gggttttttg

3661 tgcgtacttc attatgtata ttaatacgac tcactatagg gagaagatct ataaaggagg

3721 taaataatgg gtcaccacca tcatcaccat agcggtggaa ccgaactgaa aatcggcaac

3781 gaaaaagtga atagcaccaa ctttggtgat tttgccgaaa aagcaattcg cggtattaac

3841 cataaaccgt tcgttaatag caaaggtggc gaacagaaaa ttaccaccag taaaattcgt

3901 ggcattctgg aactggtgaa caaagtttat aatcgcgtga tcaataccaa cgatgttgaa

3961 ctgagcgaaa atattctggc agatatcgcc tacatcaaag tgaaaattgc ttatgaaagc

4021 ggtcgtgaac cggtggtgaa agattttatt cagcgtaccg catttaccgc agcaattacc

4081 gatgttatga atcagcgcac ccgtgaaagt tttctgctgt ttgcacgtta tgttgaaagc

4141 ctgatcgcct atttcaaatt ctatggtggc aaagattaaa cgcgtgctgc gtggtcaaat

4201 gtgcgtagac caaccccttg cggcctcaat cgggggggat ggggtttttt gtcaggcaag

4261 tctcagctgg tttaatacga ctcactatag ggagagaatt cataaaggag gtaaataatg

4321 aaactggtta ttgaaggcac cattgttctg aaaaccggta tgcatattgg tggtagcagc

4381 gattttagcg caattggtgc agttgatagt ccggttgttc gtgataccct gacccgtctg

4441 ccgctgattc cgggtagcag cctgaaaggt aaaatgcgtt atctgctggc aaaagaactg

4501 aataatggca ttctgctgaa tgagccgaat aacgatcagg atgaaattct gcgtctgttt

4561 ggtagctccg agaaagataa aattcgtcgt gcacgtctga aattcaacga tattaaactg

4621 agcaatctgg ccgaactgga aacctttaat gttagcagca ccgaagtgaa atttgagaat

4681 accattaatc gcaaaaccgc agttgcaaat ccgcgtcaga ttgaacgtgt tattgcaggt

4741 agcaaattcg acttcgaaat cttctataac ctggatgaca tcaaagaagt ggaaaaagac

4801 ttcgagaaca tcaaacaggg ttttgacctg ctggaatttg attatctggg tggtcatggc

4861 acccgtggta gcggtcgtat tgcatttgaa aatctgagcg ttattaccgc agtgggcaac

4921 tttgaaaaaa tcaataccct gaacgaaatc ctgggtgcct aaaagcttac ctggagatca

4981 aggagattac tctaacccca tcggccgtct taggggtttt ttgtcctgtg ttagctggag

5041 ggtataatac gactcactat agggagaccc gggataaagg aggtaaataa tgaaaatcat

5101 caaactgtac ttcgaaagtc cggtgcattt tggtgaaaaa cgtctgagcg aaagcaaaat

5161 tacctttagc gcagataccc tgtttagcgc actgatgatt gaagcagttg gtctgggtaa

5221 agaagatgag ttttatcagc tggcaagcaa caacctggtg aaatttagtg atgcctttcc

5281 gttcatcgat cagtattatt acattccgaa accgatgttt aacctgaaac tggaaaaaga

5341 ggatgagaat ccgagcaaag cattcaaaaa actgctgtat gttccgatcg atagcctgga

5401 agattatctg agcggtggtc tggatgcata ttttgaacgt gaatcattta acctgggtaa

5461 actggcactg agtgaaaaag ttcagcagca cgattttaaa gatagcgaac cgtataatgt

5521 tggcaccttt acctttaaag aaaacaccgg tctgtatgtg ctgattgaac agacccatcc

5581 gctgctggaa gaactgctgg aaaatctgca gtatagcggt attggtggta aacgtaatag

5641 cggttatggc aaattcaaat tcgagattct ggaagatagt gacatcgagg acctgtttag

5701 tgcaaaaggt aatcgtaaaa ttctgctgag tggtgcactg ccgaaagatg cagaactgga

5761 acaggcactg aaaaatgcaa gctatctgct ggaacgtcgt ggtggttttg ttcagagcga

5821 tacctatgca accaatctgg ttaaaaaaca ggatctgtac gtgtttaaaa gcggcagcac

5881 ctttgaaaat agctttgatg gtgatattta tcaggtgggc aaaaaaggca atcatccggt

5941 ttacaaatac gccaaatcct tttttctgga agtgagcgtg taatcatgat ttcttgtcga

6001 actggacagt agcagaaccg ctaacggggg cgaaggggtt ttttgtgaca tacgagctga

6061 ttgaactaat acgactcact atagggagag gtaccataaa ggaggtaaat aatgaaaaaa

6121 acctatcgtg ttaccctgac cgcactgggt ccgattttta tcggtggtgg tgaaaaactg

6181 aaaaaatacg agtatatctt tgataaacag aaaaaagtgg cccacatgat cgaccatacc

6241 aaatttacca aatatctgct ggaaaaaaat ctgctggatg attttaccag ccgtgtgaat

6301 agccatttcg atctgtatga ttacctggtg aacaaaaaag gcattgtgtt tatgccgctg

6361 gtgaaatata gcgttccggt tgcacagttt cgtaccgaag ttaaaaatcg ttttggcaaa

6421 ccgattagca gccctccgat gaatgatctg aatacctttg ttaaagatgc ctttggccgt

6481 ccgtatattc cgggtagcag cctgaaaggt gcactgcgta ccgcaattct gaacgatctg

6541 aaagaagata ccaaagaaaa cgaagtgttt gcccatctgc aggttagcga tagcgaaacc

6601 attgatctgg aaaacctgaa agtgtatcag aaagtggatt atagcaaaac cgccaaaccg

6661 ctgccgctgt atcgtgaatg tctgaaaccg aataccgaaa ttacctttac cgtgagcttt

6721 gatgatgagt atctgaccct gaaaaaaatc cagaatgcac tgcataaaac ctaccagcac

6781 tattacatca aatggctgaa aggcggtaaa gttggtgaaa ccctgattaa aggtgtgtat

6841 gatagccatg cagacgagct gaaaaaaaac acctttgcac tggatcagcc gagccagaat

6901 cagggtgaaa ttatctatat tggtggtggc gcaggttttg tgagcaaaac cctgcactat

6961 aaaagcaaaa atcgtgatca ggcacgcaac gatagctttg atattctgaa acaactgttc

7021 cgtaccacct atagtaaaat gcgtagcgtg ccggataatg ttccggtggc actgaaactg

7081 gcagttgaaa ccaaaacctt taatggtcgt gtgaccggta aacactatct ggaaatgggt

7141 aaagcacgca tcaaactgga agaactgaaa taacctaggc gcttcaacgg aacggatctt

7201 acatatcggg ggggtagggg ttttttgtct cggagaccaa gtagggcata atacgactca

7261 ctatagggag accatggata aaggaggtaa ataatgaaaa tcctgattag cgcagttggt

7321 gataccgatc cgattcgtaa ttttcacgat ggtccgctgc tgcatattgt tcgtgtttat

7381 cgtccggaaa aaattgttct ggttcatagc gaacgtagcc tgaccaaaca tgataaactg

7441 gttaaagccc tgaaaagcat caaagattat agcccagaaa ttatccagga tggtgttgtt

7501 ctgccggatg cacaggttgc aatttttgat gaaatgtatg ataccgtgag cagcattgtg

7561 aaaaaataca ttagcgacga tgagatcatc ctgaatatta gcagcgcaac accgcagatt

7621 attagcgcaa tgtttgcagt taatcgcatc agcgatttca atgttaccgc agttcaggtt

7681 aaaacccctc agcataaaag caatgaaggt ctgcgccatg ataaccaaga agatatcgat

7741 aaactgatcg aaaccaatct ggataaccag agcgattatg aaaatcgtac cctggcagat

7801 accggcatga aattttcaca ggatctgaca aaacgtaacc tgaaagcgct gattgataac

7861 tatgattatc agggtgcact ggaactgctg aaaaaacaga aatccttcag caacattaaa

7921 gaactgcgca aaaaactgac cgaaatcagc gataccatca aaattcaggg tatgccggat

7981 aaaatcgtta aaagcaaact gagcaatcag gcaaaaagcg cactgaatag ctatctgaat

8041 attgatcgca atcacaaaca gggcaacatt gccgaagttc tgattcgtgt taaaagcctg

8101 gttgagttta tcctggaaga ttatctgaac aaccacttcc tggatgtgat cacctataaa

8161 gatggtaaac cgtttctgaa cgcaagcaaa tatccggaaa tcctgaaaaa attccaagag

8221 gatgcagaaa tgcgtggcaa agaatatcat agcggttatc tgagcctgcc tgcatatatt

8281 ggtatcctga aatttttcga accgaaccac gatctgctga aacacatcta taaaatccaa

8341 gagattaacc aggaccgcaa taaagttgca catagtctgc aggcatttga ccgcaaaaat

8401 ctgaaaaaag tgagcagcgc agttttcgcc agcaaacaaa ttctgctggc gagctttgat

8461 attgataacc actggtttag cttctacgag gatctgaatc aagaaatcaa aaaactgctg

8521 taactcgaga ggttacagcc tgcataatgt agcataaccc cttggggcct ctaaacgggt

8581 cttgaggggt tttttgtgcc tatagtttga agcagaaaga atcgaatttc tgccattcat

8641 ccgcttatta tcacttattc aggcgtagca ccaggcgttt aagggcacca ataactgcct

8701 tacaaaaaac ccctagccgc ccgataagag cgggctaggg gttcgagtaa aaaaaattac

8761 gccccgccct gccactcatc gcagtactgt tgtaattcat taagcattct gccgacatgg

8821 aagccatcac agacggcatg atgaacctga atcgccagcg gcatcagcac cttgtcgcct

8881 tgcgtataat atttgcccat cgtgaaaacg ggggcgaaga agttgtccat attggccacg

8941 tttaaatcaa aactggtgaa actcacccag ggattggctg aaacgaaaaa catattctca

9001 ataaaccctt tagggaaata ggccaggttt tcaccgtaac acgccacatc ttgcgaatat

9061 atgtgtagaa actgccggaa atcgtcgtgg tattcactcc agagcgatga aaacgtttca

9121 gtttgctcat ggaaaacggt gtaacaaggg tgaacactat cccatatcac cagctcaccg

9181 tctttcattg ccatacggaa ctccgggtga gcattcatca ggcgggcaag aatgtgaata

9241 aaggccggat aaaacttgtg cttatttttc tttacggtct ttaaaaaggc cgtaatatcc

9301 agctgaacgg tctggttata ggtacattga gcaactgact gaaatgcctc aaaatgttct

9361 ttacgatgcc attgggatat atcaacggtg gtatatccag tgattttttt ctccatttta

9421 gcttccttag ctcctgaaaa tctcgataac tcaaaaaata cgcccggtag tgatcttatt

9481 tcattatggt gaaagttgga acctcttacg tgccgatcaa ggtctcattt tcgccaaaag

9541 ttggcccagg gcttcccggt atcaacaggg acaccaggat ttatttattc tgcgaagtga

9601 tcttccgtca caggtattta ttcggcgcaa agtgcgtcgg gtgatgctgc caacttactg

9661 atttagtgta tgatggtgtt tttgaggtgc tccagtggct tctgtttcta tcagctgtcc

9721 ctcctgttca gctactgacg gggtggtgcg taacggcaaa agcaccgccg gacatcagcg

9781 ctagcggagt gtatactggc ttactatgtt ggcactgatg agggtgtcag tgaagtgctt

9841 catgtggcag gagaaaaaag gctgcaccgg tgcgtcagca gaatatgtga tacaggatat

9901 attccgcttc ctcgctcact gactcgctac gctcggtcgt tcgactgcgg cgagcggaaa

9961 tggcttacga acggggcgga gatttcctgg aagatgccag gaagatactt aacagggaag

10021 tgagagggcc gcggcaaagc cgtttttcca taggctccgc ccccctgaca agcatcacga

10081 aatctgacgc tcaaatcagt ggtggcgaaa cccgacagga ctataaagat accaggcgtt

10141 tccccctggc ggctccctcg tgcgctctcc tgttcctgcc tttcggttta ccggtgtcat

10201 tccgctgtta tggccgcgtt tgtctcattc cacgcctgac actcagttcc gggtaggcag

10261 ttcgctccaa gctggactgt atgcacgaac cccccgttca gtccgaccgc tgcgccttat

10321 ccggtaacta tcgtcttgag tccaacccgg aaagacatgc aaaagcacca ctggcagcag

10381 ccactggtaa ttgatttaga ggagttagtc ttgaagtcat gcgccggtta aggctaaact

10441 gaaaggacaa gttttggtga ctgcgctcct ccaagccagt tacctcggtt caaagagttg

10501 gtagctcaga gaaccttcga aaaaccgccc tgcaaggcgg ttttttcgtt ttcagagcaa

10561 gagattacgc gcagaccaaa acgatctcaa gaagatcatc ttattaatca gataaaatat

10621 ttctagattt cagtgcaatt tatctcttca aatgtagcac ctgaagtcag ccccatacga

10681 tataagttgt aattctcatg tttgacagc

//

**Figure B in S1 file. *Staphylococcus epidermidis* (SEP) Csm module.** SEP system genes *csm1-6* and cas6 (orange arrows) cloned into a pACYC-based vector, along with minimal a CRISPR spacer (yellow), flanked by repeat sequences (black). Green arrows and red rectangles indicate transcriptional promoters or terminators, respectively. Chloramphenicol resistance cassette (CmR) and p15A origin of replication region (p15A) are indicated with blue or pink arrows, respectively. Plasmid map illustration made using the SnapGene program. Plasmid sequence information is in GenBank format, using ApE program.

**
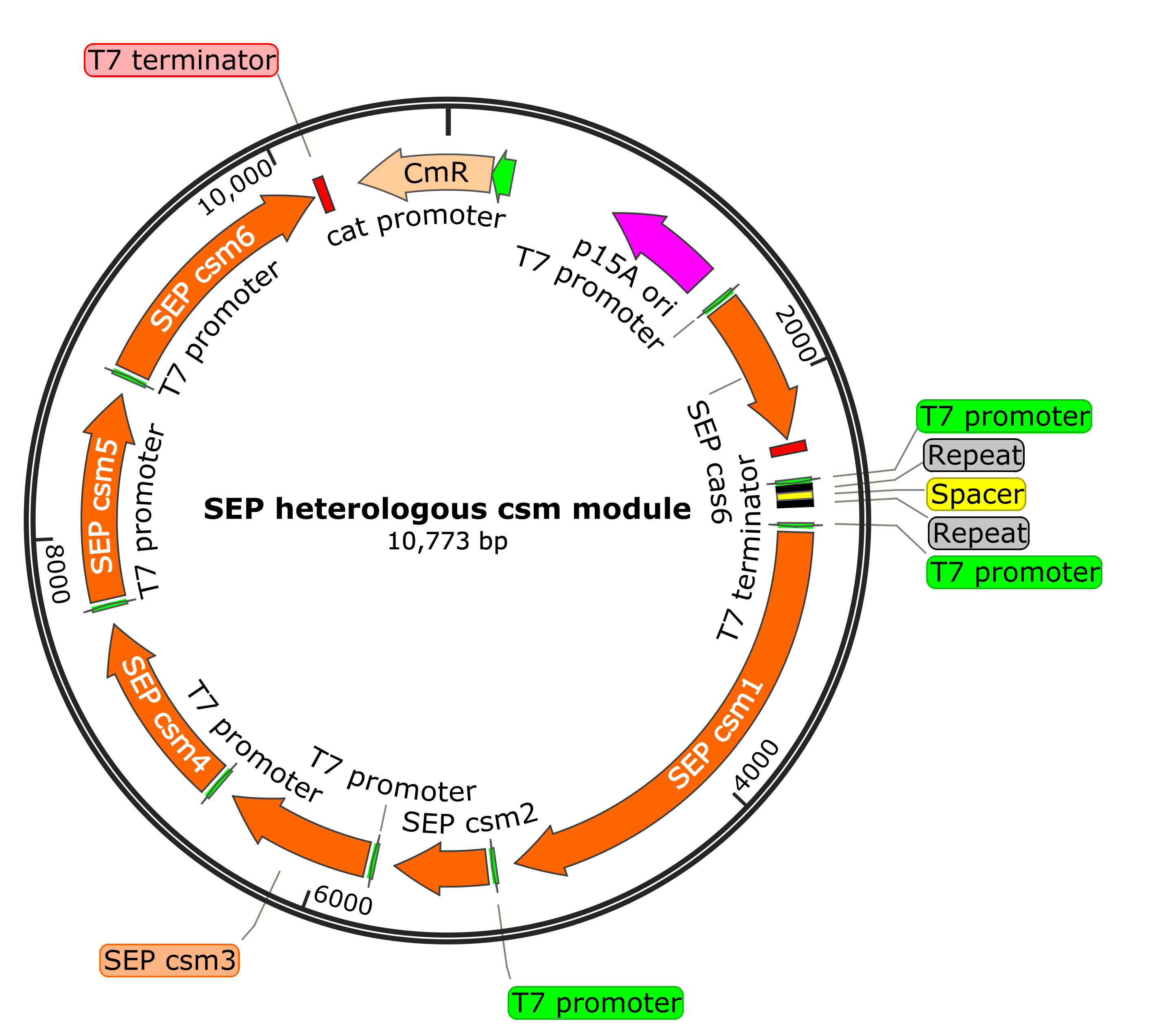
**

LOCUS SEP_heterologous 10773 bp ds-DNA circular 19-FEB-2017

DEFINITION synthetic circular DNA

ACCESSION .

VERSION .

KEYWORDS SEP heterologous csm module

SOURCE synthetic DNA construct

ORGANISM synthetic DNA construct

REFERENCE 1 (bases 1 to 10773)

AUTHORS .

TITLE Direct Submission

JOURNAL Exported Sunday, Feb 19, 2017 from SnapGene 3.3.3

http://www.snapgene.com

COMMENT

COMMENT

COMMENT ApEinfo:methylated:1

FEATURES Location/Qualifiers

promoter complement(220..322)

/note="cat promoter"

/note="promoter of the E. coli cat gene"

/label=cat promoter

/ApEinfo_fwdcolor=#00ff00

/ApEinfo_revcolor=#00ff00

/ApEinfo_graphicformat=arrow_data {{0 1 2 0 0 -1} {} 0}

width 5 offset 0

rep_origin complement(848..1393)

/direction=LEFT

/note="p15A ori"

/note="Plasmids containing the medium-copy-number p15A

origin of replication can be propagated in E. coli cells

that contain a second plasmid with the ColE1 origin."

/label=p15A ori

/ApEinfo_fwdcolor=#ff00ff

/ApEinfo_revcolor=#ff00ff

/ApEinfo_graphicformat=arrow_data {{0 1 2 0 0 -1} {} 0}

width 5 offset 0

promoter 1517..1535

/note="T7 promoter"

/note="promoter for bacteriophage T7 RNA polymerase"

/label=T7 promoter

/ApEinfo_fwdcolor=#00ff00

/ApEinfo_revcolor=#00ff00

/ApEinfo_graphicformat=arrow_data {{0 1 2 0 0 -1} {} 0}

width 5 offset 0

CDS 1556..2290

/codon_start=1

/note="SEP cas6"

/translation="MINKITVELDLPESIRFQYLGSVLHGVLMDYLSDDIADQLHHEFAYSPLKQRIYHKNKKIIWEIVCMSDNLFKEVVKLFSSKNSLLLKYYQTNIDIQSFQIEKINVQNMMNQLLQVEDLSRYVRLNIQTPMSFKYQNSYMIFPDVKRFFRSIMIQFDAFFEEYRMYDKETLNFLEKNVNIVDYKLKSTRFNLEKVKIPSFTGEIVFKIKGPLPFLQLTHFLLKFGEFSGSGIKTSLGMGKYSII"

/label=SEP cas6

/ApEinfo_fwdcolor=#ff8000

/ApEinfo_revcolor=pink

/ApEinfo_graphicformat=arrow_data {{0 1 2 0 0 -1} {} 0}

width 5 offset 0

terminator 2316..2363

/note="T7 terminator"

/note="transcription terminator for bacteriophage T7 RNA

polymerase"

/label=T7 terminator

/ApEinfo_fwdcolor=#ff0000

/ApEinfo_revcolor=pink

/ApEinfo_graphicformat=arrow_data {{0 1 2 0 0 -1} {} 0}

width 5 offset 0

promoter 2491..2509

/note="T7 promoter"

/note="promoter for bacteriophage T7 RNA polymerase"

/label=T7 promoter(1)

/ApEinfo_label=T7 promoter

/ApEinfo_fwdcolor=#80ff00

/ApEinfo_revcolor=#80ff00

/ApEinfo_graphicformat=arrow_data {{0 1 2 0 0 -1} {} 0}

width 5 offset 0

misc_feature 2520..2555

/note="Repeat"

/label=Repeat

/ApEinfo_fwdcolor=#000000

/ApEinfo_revcolor=#000000

/ApEinfo_graphicformat=arrow_data {{0 1 2 0 0 -1} {} 0}

width 5 offset 0

misc_feature 2556..2590

/note="Spacer"

/label=Spacer

/ApEinfo_fwdcolor=#ffff00

/ApEinfo_revcolor=#ffff00

/ApEinfo_graphicformat=arrow_data {{0 1 2 0 0 -1} {} 0}

width 5 offset 0

misc_feature 2591..2626

/note="Repeat"

/label=Repeat(1)

/ApEinfo_label=Repeat

/ApEinfo_fwdcolor=#000000

/ApEinfo_revcolor=#000000

/ApEinfo_graphicformat=arrow_data {{0 1 2 0 0 -1} {} 0}

width 5 offset 0

promoter 2706..2724

/note="T7 promoter"

/note="promoter for bacteriophage T7 RNA polymerase"

/label=T7 promoter(2)

/ApEinfo_label=T7 promoter

/ApEinfo_fwdcolor=#00ff00

/ApEinfo_revcolor=#00ff00

/ApEinfo_graphicformat=arrow_data {{0 1 2 0 0 -1} {} 0}

width 5 offset 0

CDS 2751..5054

/codon_start=1

/note="SEP csm1"

/translation="MGHHHHHHSGGNKKNILMYGSLLHDIGKIIYRSGDHTFSRGTHSKLGHQFLSQFSEFKDNEVLDNVAYHHYKELAKANLDNDNTAYITYIADNIASGIDRRDIIEEGDEEYEKQLFNFDKYTPLYSVFNIVNSEKLKQTNGKFKFSNESNIEYPKTENIQYSSGNYTTLMKDMSHDLEHKLSIKEGTFPSLLQWTESLWQYVPSSTNKNQLIDISLYDHSRITCAIASCIFDYLNENNIHNYKDELFSKYENTKSFYQKEAFLLLSMDMSGIQDFIYNISGSKALKSLRSRSFYLELMLEVIVDQLLERLELARANLLYTGGGHAYLLVSNTDKVKKKITQFNNELKKWFMSEFTTDLSLSMAFEKCSGDDLMNTSGNYRTIWRNVSSKLSDIKAHKYSAEDILKLNHFHSYGDRECKECLRSDIDINDDGLCSICEGIINISNDLRDKSFFVLSETGKLKMPFNKFISVIDYEEAEMLVQNNNQVRIYSKNKPYIGIGISTNLWMCDYDYASQNQDMREKGIGSYVDREEGVKRLGVVRADIDNLGATFISGIPEKYNSISRTATLSRQLSLFFKYELNHLLENYQITAIYSGGDDLFLIGAWDDIIEASIYINDKFKEFTLDKLTLSAGVGMFSGKYPVSKMAFETGRLEEAAKTGEKNQISLWLQEKVYNWDEFKKNILEEKLLVLQQGFSQTDEHGKAFIYKMLALLRNNEAINIARLAYLLARSKMNEDFTSKIFNWAQNDKDKNQLITALEYYIYQIREAD"

/label=SEP csm1

/ApEinfo_fwdcolor=#ff8000

/ApEinfo_revcolor=#ff8000

/ApEinfo_graphicformat=arrow_data {{0 1 2 0 0 -1} {} 0}

width 5 offset 0

promoter 5148..5166

/note="T7 promoter"

/note="promoter for bacteriophage T7 RNA polymerase"

/label=T7 promoter(3)

/ApEinfo_label=T7 promoter

/ApEinfo_fwdcolor=#00ff00

/ApEinfo_revcolor=#00ff00

/ApEinfo_graphicformat=arrow_data {{0 1 2 0 0 -1} {} 0}

width 5 offset 0

CDS 5193..5648

/codon_start=1

/note="SEP csm2"

/translation="MGHHHHHHSGGILAKTKSGKTIDLTFAHEVVKSNVKNVKDRKGKEKQVLFNGLTTSKLRNLMEQVNRLYTIAFNSNEDQLNEEFIDELEYLKIKFYYEAGREKSVDEFLKKTLMFPIIDRVIKKESKKFFLDYCKYFEALVAYAKYYQKED"

/label=SEP csm2

/ApEinfo_fwdcolor=#ff8000

/ApEinfo_revcolor=#ff8000

/ApEinfo_graphicformat=arrow_data {{0 1 2 0 0 -1} {} 0}

width 5 offset 0

promoter 5742..5760

/note="T7 promoter"

/note="promoter for bacteriophage T7 RNA polymerase"

/label=T7 promoter(4)

/ApEinfo_label=T7 promoter

/ApEinfo_fwdcolor=#00ff00

/ApEinfo_revcolor=#00ff00

/ApEinfo_graphicformat=arrow_data {{0 1 2 0 0 -1} {} 0}

width 5 offset 0

CDS 5787..6521

/codon_start=1

/note="SEP csm3"

/translation="MGFFVRQVSAGLIRLTIGREFPSSITGGHQMYSKIKISGTIEVVTGLHIGGGGESSMIGAIDSPVVRDLQTKLPIIPGSSIKGKMRNLLAKHFGLKMKQESHNQDDERVLRLFGSSEKGNIQRARLQISDAFFSEKTKEHFAQNDIAYTETKFENTINRLTAVANPRQIERVTRGSEFDFVFIYNVDEESQVEDDFENIEKAIHLLENDYLGGGGTRGNGRIQFKDTNIETVVGEYDSTNLKIK"

/label=SEP csm3

/ApEinfo_fwdcolor=#ff8000

/ApEinfo_revcolor=#ff8000

/ApEinfo_graphicformat=arrow_data {{0 1 2 0 0 -1} {} 0}

width 5 offset 0

promoter 6604..6622

/note="T7 promoter"

/note="promoter for bacteriophage T7 RNA polymerase"

/label=T7 promoter(5)

/ApEinfo_label=T7 promoter

/ApEinfo_fwdcolor=#00ff00

/ApEinfo_revcolor=#00ff00

/ApEinfo_graphicformat=arrow_data {{0 1 2 0 0 -1} {} 0}

width 5 offset 0

CDS 6649..7563

/codon_start=1

/note="SEP csm4"

/translation="MTLATKVFKLSFKTPVHFGKKRLSDGEMTITADTLFSALFIETLQLGKDTDWLLNDLIISDTFPYENELYYLPKPLIKIDSKEEDNHKAFKKLKYVPVHHYNQYLNGELSAEDATDLNDIFNIGYFSLQTKVSLIAQETDSSADSEPYSVGTFTFEPEAGLYFIAKGSEETLDHLNNIMTALQYSGLGGKRNAGYGQFEYEIINNQQLSKLLNQNGKHSILLSTAMAKKEEIESALKEARYILTKRSGFVQSTNYSEMLVKKSDFYSFSSGSVFKNIFNGDIFNVGHNGKHPVYRYAKPLWLEV"

/label=SEP csm4

/ApEinfo_fwdcolor=#ff8000

/ApEinfo_revcolor=#ff8000

/ApEinfo_graphicformat=arrow_data {{0 1 2 0 0 -1} {} 0}

width 5 offset 0

promoter 7647..7665

/note="T7 promoter"

/note="promoter for bacteriophage T7 RNA polymerase"

/label=T7 promoter(6)

/ApEinfo_label=T7 promoter

/ApEinfo_fwdcolor=#00ff00

/ApEinfo_revcolor=#00ff00

/ApEinfo_graphicformat=arrow_data {{0 1 2 0 0 -1} {} 0}

width 5 offset 0

CDS 7692..8714

/codon_start=1

/note="SEP csm5"

/translation="MTIKNYEVVIKTLGPIHIGSGQVMKKQDYIYDFYNSKVYMINGNKLVKFLKRKNLLYTYQNFLRYPPKNPRENGLKDYLDAQNVKQSEWEAFVSYSEKVNQGKKYGNTRPKPLNDLHLMVRDGQNKVYLPGSSIKGAIKTTLVSKYNNEKNKDIYSKIKVSDSKPIDESNLAIYQKIDINKSEKSMPLYRECIDVNTEIKFKLTIEDEIYSINEIEQSIQDFYKNYYDKWLVGFKETKGGRRFALEGGIPDVLNQNILFLGAGTGFVSKTTHYQLKNRKQAKQDSFEILTKKFRGTYGKMKEIPSNVPVALKGTTNQSRHTSYQQGMCKVSFQELNNEVL"

/label=SEP csm5

/ApEinfo_fwdcolor=#ff8000

/ApEinfo_revcolor=#ff8000

/ApEinfo_graphicformat=arrow_data {{0 1 2 0 0 -1} {} 0}

width 5 offset 0

promoter 8790..8808

/note="T7 promoter"

/note="promoter for bacteriophage T7 RNA polymerase"

/label=T7 promoter(7)

/ApEinfo_label=T7 promoter

/ApEinfo_fwdcolor=#00ff00

/ApEinfo_revcolor=#00ff00

/ApEinfo_graphicformat=arrow_data {{0 1 2 0 0 -1} {} 0}

width 5 offset 0

CDS 8835..10103

/codon_start=1

/note="SEP csm6"

/translation="MKILFSPIGNSDPWRNDRDGAMLHIVRHYNLDKVVLYFTRTIWEGNENRKGHKIYEWEKIIQTVSPNTEVEIIIENVDNAQDYDVFKEKFHKYLKIIEDSYEDCEIILNVTSGTPQMESTLCLEYIVYPENKKCVQVSTPTKDSNAGIEYSNPKDKVEEFEIVNEVEKKSEKRCKEINILSFREAMIRSQILGLIDNYDYEGALNLVSNQKSFRNGKLLRKKLLSLTKQIKTHEVFPEINEKYRDDALKKSLFHYLLLNMRYNRLDVAETLIRVKSIAEFILKTYIEIHWPTLIIEKDGKPYLNDEDNLSFVYKYNLLLEKRKQNFDVSRILGLPAFIDILTILEPNSQLLKEVNAVNDINGLRNSIAHNLDTLNLDKNKNYKKIMLSVEAIKNMLHISFPEIEEEDYNYFEEKNKEFKELL"

/label=SEP csm6

/ApEinfo_fwdcolor=#ff8000

/ApEinfo_revcolor=#ff8000

/ApEinfo_graphicformat=arrow_data {{0 1 2 0 0 -1} {} 0}

width 5 offset 0

terminator 10130..10176

/note="T7 terminator"

/note="transcription terminator for bacteriophage T7 RNA

polymerase"

/label=T7 terminator(1)

/ApEinfo_label=T7 terminator

/ApEinfo_fwdcolor=#ff0000

/ApEinfo_revcolor=#ff0000

/ApEinfo_graphicformat=arrow_data {{0 1 2 0 0 -1} {} 0}

width 5 offset 0

CDS complement(join(10333..10773,1..219))

/codon_start=1

/gene="cat"

/product="chloramphenicol acetyltransferase"

/note="CmR"

/note="confers resistance to chloramphenicol"

/translation="MEKKITGYTTVDISQWHRKEHFEAFQSVAQCTYNQTVQLDITAFLKTVKKNKHKFYPAFIHILARLMNAHPEFRMAMKDGELVIWDSVHPCYTVFHEQTETFSSLWSEYHDDFRQFLHIYSQDVACYGENLAYFPKGFIENMFFVSANPWVSFTSFDLNVANMDNFFAPVFTMGKYYTQGDKVLMPLAIQVHHAVCDGFHVGRMLNELQQYCDEWQGGA"

/label=chloramphenicol acetyltransferase

/ApEinfo_fwdcolor=#ffcc66

/ApEinfo_revcolor=#ffcc66

/ApEinfo_graphicformat=arrow_data {{0 1 2 0 0 -1} {} 0}

width 5 offset 0

ORIGIN

1 gaactccggg tgagcattca tcaggcgggc aagaatgtga ataaaggccg gataaaactt

61 gtgcttattt ttctttacgg tctttaaaaa ggccgtaata tccagctgaa cggtctggtt

121 ataggtacat tgagcaactg actgaaatgc ctcaaaatgt tctttacgat gccattggga

181 tatatcaacg gtggtatatc cagtgatttt tttctccatt ttagcttcct tagctcctga

241 aaatctcgat aactcaaaaa atacgcccgg tagtgatctt atttcattat ggtgaaagtt

301 ggaacctctt acgtgccgat caaggtctca ttttcgccaa aagttggccc agggcttccc

361 ggtatcaaca gggacaccag gatttattta ttctgcgaag tgatcttccg tcacaggtat

421 ttattcggcg caaagtgcgt cgggtgatgc tgccaactta ctgatttagt gtatgatggt

481 gtttttgagg tgctccagtg gcttctgttt ctatcagctg tccctcctgt tcagctactg

541 acggggtggt gcgtaacggc aaaagcaccg ccggacatca gcgctagcgg agtgtatact

601 ggcttactat gttggcactg atgagggtgt cagtgaagtg cttcatgtgg caggagaaaa

661 aaggctgcac cggtgcgtca gcagaatatg tgatacagga tatattccgc ttcctcgctc

721 actgactcgc tacgctcggt cgttcgactg cggcgagcgg aaatggctta cgaacggggc

781 ggagatttcc tggaagatgc caggaagata cttaacaggg aagtgagagg gccgcggcaa

841 agccgttttt ccataggctc cgcccccctg acaagcatca cgaaatctga cgctcaaatc

901 agtggtggcg aaacccgaca ggactataaa gataccaggc gtttccccct ggcggctccc

961 tcgtgcgctc tcctgttcct gcctttcggt ttaccggtgt cattccgctg ttatggccgc

1021 gtttgtctca ttccacgcct gacactcagt tccgggtagg cagttcgctc caagctggac

1081 tgtatgcacg aaccccccgt tcagtccgac cgctgcgcct tatccggtaa ctatcgtctt

1141 gagtccaacc cggaaagaca tgcaaaagca ccactggcag cagccactgg taattgattt

1201 agaggagtta gtcttgaagt catgcgccgg ttaaggctaa actgaaagga caagttttgg

1261 tgactgcgct cctccaagcc agttacctcg gttcaaagag ttggtagctc agagaacctt

1321 cgaaaaaccg ccctgcaagg cggttttttc gttttcagag caagagatta cgcgcagacc

1381 aaaacgatct caagaagatc atcttattaa tcagataaaa tatttctaga tttcagtgca

1441 atttatctct tcaaatgtag cacctgaagt cagccccata cgatataagt tgtaattctc

1501 atgtttgaca gccttctaat acgactcact atagggagaa taaaggaggt aaataatgat

1561 caacaaaatc accgtggaac tggatctgcc ggaaagcatt cgttttcagt atctgggtag

1621 cgttctgcat ggtgttctga tggattatct gagtgatgat attgcagatc agctgcatca

1681 cgaatttgca tatagtccgc tgaaacagcg catctaccac aaaaacaaaa aaatcatctg

1741 ggaaatcgtg tgcatgagcg ataacctgtt taaagaagtg gtgaaactgt ttagcagcaa

1801 aaatagcctg ctgctgaaat attaccagac caacattgat atccagagct tccagatcga

1861 aaaaatcaat gtgcagaaca tgatgaatca gctgctgcag gttgaggatc tgagccgtta

1921 tgttcgtctg aacattcaga ccccgatgag cttcaaatat cagaacagct atatgatctt

1981 cccggatgtg aaacgttttt tccgcagcat tatgattcag ttcgatgcct tttttgaaga

2041 ataccgcatg tacgataaag aaaccctgaa cttcctggaa aaaaacgtga acatcgtgga

2101 ttataaactg aaaagcaccc gctttaatct ggaaaaagtt aaaattccga gctttaccgg

2161 tgagatcgtg ttcaaaatca aaggtccgct gccgtttctg cagctgaccc attttctgct

2221 gaaatttggt gaatttagcg gcagcggtat taaaaccagc ctgggtatgg gtaaatatag

2281 catcatctaa aagctttcct gtgagcagcg aaagcctagc ataacccctt ggggcctcta

2341 aacgggtctt gaggggtttt ttgttatacg cgagataatc acttgcatag ctgcgtatgg

2401 aggaagcaac tcttgagtgt taatatgttg acccctgtat tagggatgcg ggtagtagat

2461 gtgggcagag acacccacac tgccagatct taatacgact cactataggg agaccatggg

2521 atcgataccc accccgaaga aaaggggacg agaacacgta tgccgaagta tataaatcat

2581 cagtacaaag gatcgatacc caccccgaag aaaaggggac gagaacctcg aggctgtggt

2641 ctagacattc catacatatc gggggggtag gggttttttg tgtgcctcta gtggctggct

2701 aagaataata cgactcacta tagggagagg atccataaag gaggtaaata atgggtcacc

2761 accatcatca ccatagcggt ggaaacaaaa aaaacatcct gatgtatggc agcctgctgc

2821 atgatattgg caaaattatc tatcgtagcg gtgatcatac ctttagccgt ggcacccata

2881 gcaaactggg tcatcagttt ctgagccagt ttagcgaatt taaagataac gaagtgctgg

2941 ataacgtggc ctatcatcat tataaagaac tggcaaaagc caacctggat aatgataata

3001 ccgcctacat tacctatatc gccgataata ttgcaagcgg tattgatcgt cgcgatatta

3061 ttgaagaggg tgatgaagaa tatgagaaac aactgttcaa cttcgataaa tacacaccgc

3121 tgtatagcgt gtttaacatt gtgaatagcg aaaaactgaa acagaccaac ggcaaattca

3181 aatttagcaa cgaaagcaac atcgaatacc cgaaaaccga aaacattcag tatagcagcg

3241 gtaattatac caccctgatg aaagatatga gccatgatct ggaacataaa ctgagcatta

3301 aagaaggcac ctttccgagt ctgctgcagt ggaccgaaag cctgtggcag tatgttccga

3361 gcagcaccaa taaaaaccag ctgattgata tcagcctgta tgaccatagc cgtattacct

3421 gtgcaattgc cagctgcatt tttgattatc tgaacgagaa caacatccac aactataaag

3481 atgaactgtt tagcaaatat gaaaacacca aatcctttta tcagaaagag gcatttctgc

3541 tgctgagcat ggatatgagc ggtattcagg atttcatcta taacattagc ggtagcaaag

3601 cactgaaaag cctgcgtagc cgtagctttt atctggaact gatgctggaa gttattgttg

3661 atcagctgct ggaacgcctg gaactggcac gtgcaaatct gctgtatacc ggtggtggtc

3721 atgcatatct gctggttagc aataccgaca aagtgaaaaa aaaaatcacc cagttcaaca

3781 acgaactgaa aaaatggttt atgagcgagt ttaccaccga tctgagcctg tcaatggcat

3841 ttgaaaaatg tagtggtgat gacctgatga ataccagcgg caattatcgt accatttggc

3901 gtaatgttag cagcaaactg agcgatatta aagcccacaa atatagcgca gaggacattc

3961 tgaaactgaa ccattttcat agttatggcg atcgcgaatg taaagaatgt ctgcgtagcg

4021 atattgacat taacgatgat ggtctgtgta gcatttgcga aggcattatt aacatcagca

4081 atgatctgcg cgacaaatcg ttttttgtgc tgagcgaaac cggtaaactg aaaatgccgt

4141 ttaacaaatt catcagcgtg atcgattatg aagaggccga aatgctggtt cagaataata

4201 accaggttcg catctatagc aaaaacaaac cgtatattgg cattggcatt agcaccaatc

4261 tgtggatgtg tgattatgat tatgcaagcc agaatcagga tatgcgcgaa aaaggtattg

4321 gtagctatgt tgatcgtgaa gaaggtgtta aacgtctggg tgttgttcgt gcagatattg

4381 ataatctggg tgcaaccttt attagcggca ttccggaaaa atacaatagc attagccgta

4441 ccgcaaccct gagccgtcag ctgagtctgt tctttaaata cgagctgaac catctgctgg

4501 aaaactatca gattaccgca atttatagtg gcggagatga cctgtttctg attggtgcat

4561 gggatgatat tatcgaagcg agcatttaca tcaacgataa attcaaagag tttaccctgg

4621 acaaactgac cctgagtgcc ggtgttggca tgtttagcgg taaatatccg gttagcaaaa

4681 tggcctttga gacaggtcgt ctggaagagg cagcaaaaac tggcgaaaaa aaccagatta

4741 gtctgtggct gcaagagaaa gtgtataact gggatgagtt caaaaaaaac attctggaag

4801 agaaactgct ggttctgcag cagggtttta gccagaccga tgaacatggt aaagccttca

4861 tttacaaaat gctggcactg ctgcgtaata acgaagcaat taacattgca cgtctggcat

4921 acctgctggc acgtagtaaa atgaatgaag atttcaccag caaaatcttt aactgggcac

4981 agaacgacaa agacaaaaat caactgatta cagccctgga atactatatc tatcagatcc

5041 gtgaagccga ctaacatatg gctgcgtggt caaatgtgcg taccctaacc ccttccccgg

5101 tcaatcgggg cggatggggt tttttgtgcg tacttcatta tgtatattaa tacgactcac

5161 tatagggaga agatctataa aggaggtaaa taatgggtca ccaccatcat caccatagcg

5221 gtggaattct ggccaaaacc aaaagcggca aaaccattga tctgaccttt gcacatgaag

5281 tggttaaaag caatgtgaaa aacgtgaaag accgcaaagg caaagaaaaa caggttctgt

5341 ttaatggtct gaccaccagt aaactgcgta atctgatgga acaggttaat cgcctgtata

5401 ccattgcctt taatagcaat gaagatcagc tgaacgaaga gtttatcgat gaactggaat

5461 atctgaaaat caaattctac tatgaagccg gtcgtgagaa aagcgttgat gagtttctga

5521 aaaaaaccct gatgttcccg attattgatc gcgtgatcaa aaaagaaagc aaaaaattct

5581 tcctggacta ctgcaaatat ttcgaagcac tggttgcata cgccaaatat taccagaaag

5641 aggactaaac gcgtgctgcg tggtcaaatg tgcgtagacc aaccccttgc ggcctcaatc

5701 gggggggatg gggttttttg tcaggcaagt ctcagctggt ttaatacgac tcactatagg

5761 gagagaattc ataaaggagg taaataatgg ggttttttgt caggcaagtc tcagctggtt

5821 taatacgact cactataggg agagaattcc ccagcagtat aacaggagga caccagatgt

5881 acagcaaaat caaaatcagc ggcaccattg aagttgttac cggtctgcat attggtggtg

5941 gtggcgaaag cagcatgatt ggtgcaattg atagtccggt tgttcgtgat ctgcagacca

6001 aactgccgat tattccgggt agcagcatta aaggtaaaat gcgtaatctg ctggccaaac

6061 actttggcct gaaaatgaaa caagaaagcc ataaccagga tgatgaacgt gttctgcgtc

6121 tgtttggtag cagcgaaaaa ggtaatattc agcgtgctcg cctgcagatt agtgatgcat

6181 tttttagcga aaaaaccaaa gaacacttcg cccagaatga tattgcatac accgaaacca

6241 aattcgagaa taccattaat cgtctgaccg cagttgcaaa tccgcgtcag attgaacgtg

6301 tgacccgtgg tagcgaattt gactttgtgt ttatctataa cgtggatgaa gagtcccagg

6361 tggaagatga ttttgaaaac attgagaaag cgatccatct gctggaaaat gattatctgg

6421 gtggcggtgg tacacgtggt aatggtcgta ttcagtttaa agacaccaac attgaaaccg

6481 tggtgggtga atatgatagc accaatctga aaatcaaata aaagcttacc tggagatcaa

6541 ggagattact ctaaccccat cggccgtctt aggggttttt tgtcctgtgt tagctggagg

6601 gtataatacg actcactata gggagacccg ggataaagga ggtaaataat gaccctggca

6661 accaaagttt ttaaactgag ctttaaaaca ccggtgcatt tcggtaaaaa acgtctgagt

6721 gatggtgaaa tgaccattac cgcagatacc ctgtttagcg cactgtttat tgaaaccctg

6781 cagctgggta aagataccga ttggctgctg aatgatctga ttattagcga tacctttccg

6841 tatgagaacg agctgtatta tctgccgaaa ccgctgatta aaatcgacag caaagaagag

6901 gataaccaca aagccttcaa aaaactgaaa tatgtgccgg tgcatcacta taaccagtat

6961 ctgaatggtg aactgagcgc agaagatgca accgatctga atgatatttt caacatcggc

7021 tatttcagcc tgcagaccaa agttagcctg attgcacaag aaaccgatag cagcgcagat

7081 agcgaaccgt atagcgttgg cacctttacc tttgaaccgg aagcaggtct gtattttatc

7141 gcaaaaggta gcgaagaaac cctggatcat ctgaataaca ttatgaccgc actgcagtat

7201 agcggtctgg gtggtaaacg taatgcaggt tatggtcagt ttgagtacga aatcattaat

7261 aaccagcagc tgagcaaact gctgaatcag aatggtaaac atagcattct gctgagcacc

7321 gcaatggcaa aaaaagaaga aattgaaagc gcactgaaag aggcacgtta tattctgacc

7381 aaacgtagcg gttttgttca gagcaccaat tatagcgaaa tgctggtgaa aaaaagcgac

7441 ttctatagct ttagcagcgg cagcgttttc aaaaacattt ttaacggcga tatcttcaac

7501 gtgggccata atggcaaaca tccggtttat cgttatgcta aaccgctgtg gctggaagtt

7561 taatcatgat ttcttgtcga actggacagt agcagaaccg ctaacggggg cgaaggggtt

7621 ttttgtgaca tacgagctga ttgaactaat acgactcact atagggagag gtaccataaa

7681 ggaggtaaat aatgaccatc aaaaactatg aggtggtgat taaaaccctg ggtccgattc

7741 atattggtag cggtcaggtt atgaaaaaac aggattatat ctacgacttt tataacagca

7801 aagtgtatat gatcaacggc aacaaactgg tgaaatttct gaaacgcaaa aacctgctgt

7861 atacctatca gaactttctg cgttatccgc ctaaaaatcc gcgtgaaaat ggtctgaaag

7921 attatctgga tgcccagaat gttaaacaga gcgaatggga agcatttgtg agctatagcg

7981 aaaaagtgaa ccagggcaaa aaatacggta atacccgtcc gaaaccgctg aatgatctgc

8041 atctgatggt tcgtgatggt cagaataaag tttatctgcc tggtagcagc attaaaggtg

8101 caattaaaac caccctggtg agcaaatata acaacgaaaa aaacaaagat atctatagca

8161 aaatcaaagt gagcgatagc aaaccgattg atgaaagcaa tctggccatc tatcagaaaa

8221 tcgacatcaa caaaagcgag aaaagcatgc cgctgtatcg tgaatgtatt gatgtgaaca

8281 ccgagatcaa attcaaactg accatcgagg atgaaatcta cagcatcaat gaaatcgaac

8341 agagcatcca ggacttctat aaaaactact atgataaatg gctggtcggc tttaaagaaa

8401 ccaaaggtgg tcgtcgtttt gcactggaag gtggtattcc ggatgttctg aatcaaaaca

8461 ttctgtttct gggtgcaggc accggttttg tgagcaaaac cacacattat cagctgaaaa

8521 atcgcaaaca ggccaaacag gatagctttg aaattctgac gaaaaaattc cgtggcacct

8581 acggcaaaat gaaagaaatt ccgagcaatg ttccggttgc actgaaaggc accaccaatc

8641 agagccgtca taccagctat cagcagggta tgtgtaaagt tagctttcaa gaactgaaca

8701 acgaggtgct gtaacctagg cgcttcaacg gaacggatct tacatatcgg gggggtaggg

8761 gttttttgtc tcggagacca agtagggcat aatacgactc actataggga gactatggat

8821 aaaggaggta aataatgaaa atcctgttta gcccgattgg taatagcgat ccgtggcgta

8881 atgatcgtga tggtgcaatg ctgcatattg tgcgtcatta taatctggat aaagtggtgc

8941 tgtatttcac ccgtaccatt tgggaaggta atgaaaatcg caaaggccac aaaatctatg

9001 aatgggagaa aattatccag accgttagcc cgaataccga agtggaaatt atcattgaaa

9061 atgtggataa cgcccaggat tacgatgtgt tcaaagagaa attccataaa tatctgaaaa

9121 tcatcgaaga tagctacgag gattgcgaaa ttattctgaa tgttaccagc ggtacaccgc

9181 agatggaaag caccctgtgt ctggaatata ttgtttaccc ggaaaacaaa aaatgcgttc

9241 aggttagcac cccgaccaaa gatagcaatg caggtattga atatagcaac ccgaaagaca

9301 aagtggaaga atttgaaatc gtgaacgaag tcgagaaaaa aagcgaaaaa cgctgcaaag

9361 aaatcaacat tctgagcttt cgtgaagcca tgattcgtag ccagattctg ggtctgattg

9421 ataactatga ttatgaaggt gccctgaatc tggtgagcaa tcagaaaagt tttcgcaatg

9481 gtaaactgct gcgtaaaaaa ctgctgagcc tgaccaaaca aatcaaaacc catgaagttt

9541 tcccggaaat caacgaaaaa tatcgtgatg acgccctgaa aaaatccctg tttcattatc

9601 tgctgctgaa catgcgttat aatcgtctgg atgttgcaga aaccctgatt cgtgttaaaa

9661 gcattgcaga gtttatcctg aaaacctaca tcgaaattca ttggccgacc ctgattattg

9721 agaaagatgg taaaccgtat ctgaacgatg aagataatct gtccttcgtg tacaaataca

9781 acctgctgct ggaaaaacgc aaacagaatt ttgatgttag ccgtattctg ggcctgcctg

9841 catttattga tattctgacc attctggaac cgaatagcca gctgctgaaa gaagttaacg

9901 cagttaacga tattaatggc ctgcgtaata gcattgccca taacctggat accctgaacc

9961 tggacaaaaa taaaaactac aaaaaaatca tgctgagcgt ggaagccatc aaaaatatgc

10021 tgcacattag cttccctgaa atcgaggaag aggattataa ctattttgaa gagaaaaaca

10081 aagaatttaa agaactgctg taactcgaga ggttacagcc tgcataatgt agcataaccc

10141 cttggggcct ctaaacgggt cttgaggggt tttttgtgcc tatagtttga agcagaatcg

10201 aatttctgcc attcatccgc ttattatcac ttattcaggc gtagcaccag gcgtttaagg

10261 gcaccaataa ctgccttaca aaaaacccct agccgcccga taagagcggg ctaggggttc

10321 gagtaaaaaa aattacgccc cgccctgcca ctcatcgcag tactgttgta attcattaag

10381 cattctgccg acatggaagc catcacagac ggcatgatga acctgaatcg ccagcggcat

10441 cagcaccttg tcgccttgcg tataatattt gcccatcgtg aaaacggggg cgaagaagtt

10501 gtccatattg gccacgttta aatcaaaact ggtgaaactc acccagggat tggctgaaac

10561 gaaaaacata ttctcaataa accctttagg gaaataggcc aggttttcac cgtaacacgc

10621 cacatcttgc gaatatatgt gtagaaactg ccggaaatcg tcgtggtatt cactccagag

10681 cgatgaaaac gtttcagttt gctcatggaa aacggtgtaa caagggtgaa cactatccca

10741 tatcaccagc tcaccgtctt tcattgccat acg

//

**Figure C in S1 file. *Streptococcus thermophilus* (STH) Csm module.** STH system genes *csm1-6* and cas6 (orange arrows) cloned into a pACYC-based vector, along with minimal a CRISPR spacer (yellow), flanked by repeat sequences (black). Green arrows and red rectangles indicate transcriptional promoters or terminators, respectively. Chloramphenicol resistance cassette (CmR) and p15A origin of replication region (p15A) are indicated with blue or pink arrows, respectively. Plasmid map illustration made using the SnapGene program. Plasmid sequence information is in GenBank format, using ApE program.


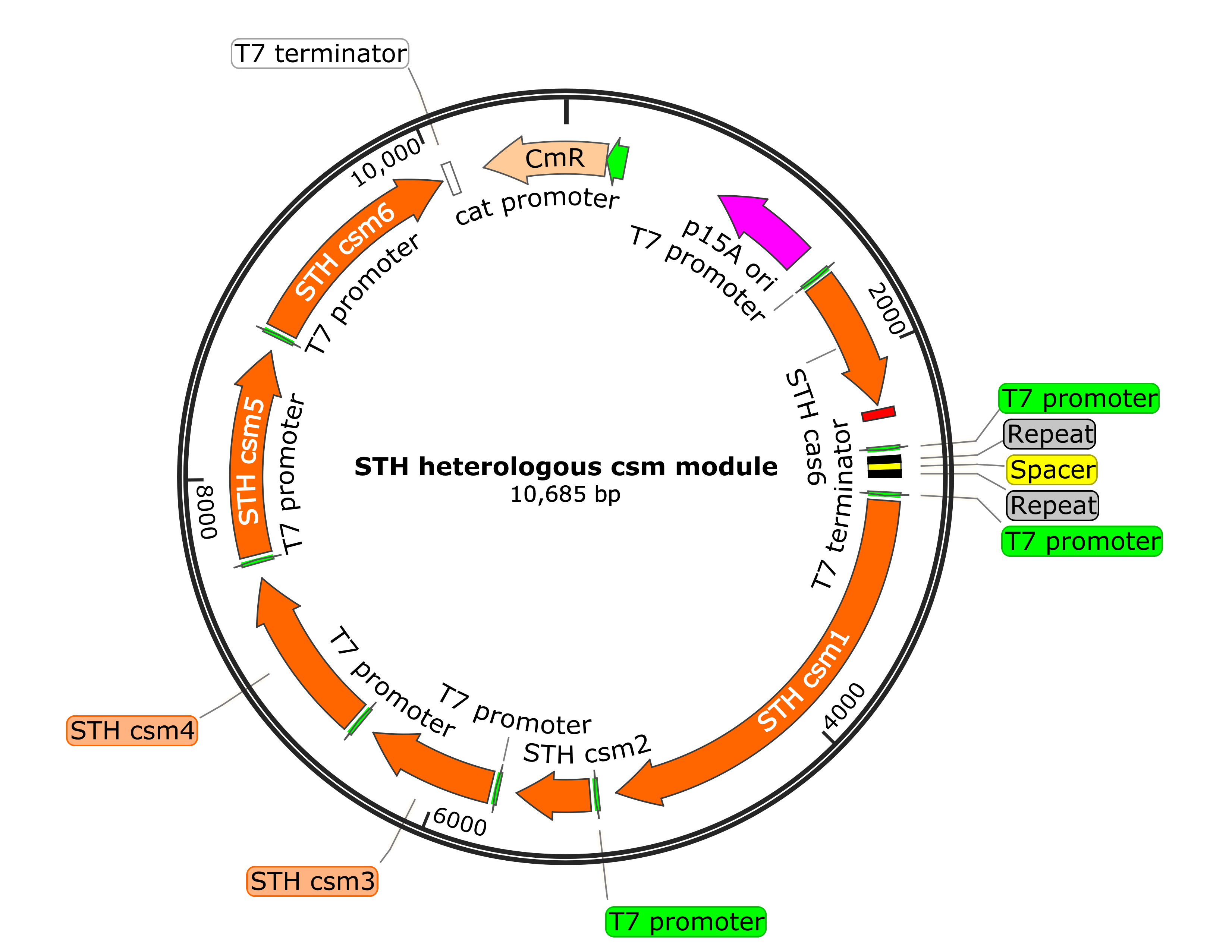


LOCUS STH_heterologous 10685 bp ds-DNA circular 19-FEB-2017

DEFINITION synthetic circular DNA

ACCESSION .

VERSION .

KEYWORDS STH heterologous csm module

SOURCE synthetic DNA construct

ORGANISM synthetic DNA construct

REFERENCE 1 (bases 1 to 10685)

AUTHORS .

TITLE Direct Submission

JOURNAL Exported Sunday, Feb 19, 2017 from SnapGene 3.3.3

http://www.snapgene.com

COMMENT

COMMENT ApEinfo:methylated:1

FEATURES Location/Qualifiers

promoter complement(220..322)

/note="cat promoter"

/note="promoter of the E. coli cat gene"

/label=cat promoter

/ApEinfo_fwdcolor=#00ff00

/ApEinfo_revcolor=#00ff00

/ApEinfo_graphicformat=arrow_data {{0 1 2 0 0 -1} {} 0}

width 5 offset 0

rep_origin complement(848..1393)

/direction=LEFT

/note="p15A ori"

/note="Plasmids containing the medium-copy-number p15A

origin of replication can be propagated in E. coli cells

that contain a second plasmid with the ColE1 origin."

/label=p15A ori

/ApEinfo_fwdcolor=#ff00ff

/ApEinfo_revcolor=#ff00ff

/ApEinfo_graphicformat=arrow_data {{0 1 2 0 0 -1} {} 0}

width 5 offset 0

promoter 1517..1535

/note="T7 promoter"

/note="promoter for bacteriophage T7 RNA polymerase"

/label=T7 promoter

/ApEinfo_fwdcolor=#00ff00

/ApEinfo_revcolor=#00ff00

/ApEinfo_graphicformat=arrow_data {{0 1 2 0 0 -1} {} 0}

width 5 offset 0

CDS 1556..2287

/codon_start=1

/note="STH cas6"

/translation="MKKLVFTFKRIDHPAQDLAVKFHGFLMEQLDSDYVDYLHQQQTNPYATKVIQGKENTQWVVHLLTDDHEDKVFMTLLQIKEVSLNDLPKLSVEKVEIQELGADKLLEIFNSEENQTYFSIIFETPTGFKSQGSYVIFPSMRLIFQSLMQKYGRLVENQPEIEEDTLDYLSEHSTITNYRLETSYFRVHRQRIPAFRGKLTFKVQGAKTLKAYVKMLLTFGEYSGLGMKTSLGMGGIKLEERKD"

/label=STH cas6

/ApEinfo_fwdcolor=#ff8000

/ApEinfo_revcolor=#ff8000

/ApEinfo_graphicformat=arrow_data {{0 1 2 0 0 -1} {} 0}

width 5 offset 0

terminator 2313..2360

/note="T7 terminator"

/note="transcription terminator for bacteriophage T7 RNA

polymerase"

/label=T7 terminator

/ApEinfo_fwdcolor=#ff0000

/ApEinfo_revcolor=#ff0000

/ApEinfo_graphicformat=arrow_data {{0 1 2 0 0 -1} {} 0}

width 5 offset 0

promoter 2511..2529

/note="T7 promoter"

/note="promoter for bacteriophage T7 RNA polymerase"

/label=T7 promoter(1)

/ApEinfo_label=T7 promoter

/ApEinfo_fwdcolor=#00ff00

/ApEinfo_revcolor=#00ff00

/ApEinfo_graphicformat=arrow_data {{0 1 2 0 0 -1} {} 0}

width 5 offset 0

misc_feature 2563..2598

/note="Repeat"

/label=Repeat

/ApEinfo_fwdcolor=#000000

/ApEinfo_revcolor=#000000

/ApEinfo_graphicformat=arrow_data {{0 1 2 0 0 -1} {} 0}

width 5 offset 0

misc_feature 2599..2637

/note="Spacer"

/label=Spacer

/ApEinfo_fwdcolor=#ffff00

/ApEinfo_revcolor=#ffff00

/ApEinfo_graphicformat=arrow_data {{0 1 2 0 0 -1} {} 0}

width 5 offset 0

misc_feature 2638..2673

/note="Repeat"

/label=Repeat(1)

/ApEinfo_label=Repeat

/ApEinfo_fwdcolor=#000000

/ApEinfo_revcolor=#000000

/ApEinfo_graphicformat=arrow_data {{0 1 2 0 0 -1} {} 0}

width 5 offset 0

promoter 2753..2771

/note="T7 promoter"

/note="promoter for bacteriophage T7 RNA polymerase"

/label=T7 promoter(2)

/ApEinfo_label=T7 promoter

/ApEinfo_fwdcolor=#00ff00

/ApEinfo_revcolor=#00ff00

/ApEinfo_graphicformat=arrow_data {{0 1 2 0 0 -1} {} 0}

width 5 offset 0

CDS 2798..5074

/codon_start=1

/note="STH csm1"

/translation="MKKEKIDLFYGALLHDIGKVIQRATGERKKHALVGADWFDEIADNQVISDQIRYHMANYQSDKLGNDHLAYITYIADNIASGVDRRQSNEESDEDASAKIWDTYTNQADIFNVFGAQTDKRYFKPTVLNLKSKPNFASATYEPFSKGDYAAIATRIKNELAEFEFNQAQIDSLLNLFEAILSFVPSSTNSKEIADISLAEHSRLTAAFALAIYDYLEDKGRHNYKEDLFTKASAFYEEEAFLLASFDLSGIQDFIYNIATSGAAKQLKARSLYLDFMSEYIADSLLDKLGLNRANLLYVGGGHAYFVLANTEKTVETLVQFEKDFNQFLLANFQTRLYVAFGWGSFAAKDIMSELNSPESYRQIYQKASRMISEKKISRYDYRTLMLLNRGGKSSERECEICHSVENLVSYHDQKVCDICRGLYQFSKEIAHDHFIITENEGLPIGPNACLKGVAFEKLSQESFSRVYVKNDYKAGTIKATHVFVGDYQCDEIHKYAALSKNEDGLGIKRLAVVRLDVDDLGAAFMAGFSRQGNGQYSTLSRSATFSRSMSLFFKVYINQFASDKKLSIIYAGGDDVFAIGSWQDIIAFTVELRQNFIKWTNGKLTLSAGIGLFADKTPISLMAHQTGELEEAAKGNEKDSISLFSSDYTFKFDRFITNVYDDKLEQIRYFFNHQDERGKNFIYKLIELLRNYESEEKMNVARLAYYLTRLEELTDKDERDKFKQFKKLFFKWYTNNESDRKEAELALLLYVYEIRKD"

/label=STH csm1

/ApEinfo_fwdcolor=#ff8000

/ApEinfo_revcolor=#ff8000

/ApEinfo_graphicformat=arrow_data {{0 1 2 0 0 -1} {} 0}

width 5 offset 0

promoter 5168..5186

/note="T7 promoter"

/note="promoter for bacteriophage T7 RNA polymerase"

/label=T7 promoter(3)

/ApEinfo_label=T7 promoter

/ApEinfo_fwdcolor=#00ff00

/ApEinfo_revcolor=#00ff00

/ApEinfo_graphicformat=arrow_data {{0 1 2 0 0 -1} {} 0}

width 5 offset 0

CDS 5213..5605

/codon_start=1

/note="STH csm2"

/translation="MAILTDENYVDKAERAISLLEKDNKGNYLLTTSQIRKLLSLCSSLYDRSKERKFDELINDVSYLRVQFVYQSGRNSVRVNRQTFFPVKDLVEKGQILEALKEIKDRETLQRFCRYMEALVAYFKFYGGKD"

/label=STH csm2

/ApEinfo_fwdcolor=#ff8000

/ApEinfo_revcolor=#ff8000

/ApEinfo_graphicformat=arrow_data {{0 1 2 0 0 -1} {} 0}

width 5 offset 0

promoter 5699..5717

/note="T7 promoter"

/note="promoter for bacteriophage T7 RNA polymerase"

/label=T7 promoter(4)

/ApEinfo_label=T7 promoter

/ApEinfo_fwdcolor=#00ff00

/ApEinfo_revcolor=#00ff00

/ApEinfo_graphicformat=arrow_data {{0 1 2 0 0 -1} {} 0}

width 5 offset 0

CDS 5744..6439

/codon_start=1

/note="STH csm3"

/translation="MGHHHHHHSGGLTFAKIKFSAQIRLETGLHIGGSDAFAAIGAIDSPVIKDPITNLPIIPGSSLKGKMRTLLAKVYNEKVAEKPSDDSDILSRLFGNSKDKRFKMGRLIFRDAFLSNADELDSLGVRSYTEVKFENTIDRITAEANPRQIERAIRNSTFDFELIYEITDENENQVEEDFKVIRDGLKLLELDYLGGSGSRGYGKVAFENLKATTVFGNYDVKTLNELLTAEV"

/label=STH csm3

/ApEinfo_fwdcolor=#ff8000

/ApEinfo_revcolor=#ff8000

/ApEinfo_graphicformat=arrow_data {{0 1 2 0 0 -1} {} 0}

width 5 offset 0

promoter 6522..6540

/note="T7 promoter"

/note="promoter for bacteriophage T7 RNA polymerase"

/label=T7 promoter(5)

/ApEinfo_label=T7 promoter

/ApEinfo_fwdcolor=#00ff00

/ApEinfo_revcolor=#00ff00

/ApEinfo_graphicformat=arrow_data {{0 1 2 0 0 -1} {} 0}

width 5 offset 0

CDS 6567..7466

/codon_start=1

/note="STH csm4"

/translation="MTYKLYIMTFQNAHFGSGTLDSSKLTFSADRIFSALVLEALKMGKLDAFLAEANQDKFTLTDAFPFQFGPFLPKPIGYPKHDQIDQSVDVKEVRRQAKLSKKLQFLALENVDDYLNGELFENEDHAVIDTVTKNQPHKDGNLYQVATTRFSNDTSLYVIANESDLLNELMSSLQYSGLGGKRSSGFGRFELDIQNIPLELSDRLTKNHSDKVMSLTTALPVDADLEEAMEDGHYLLTKSSGFAFSHATNENYRKQDLYKFASGSTFSKTFEGQIVDVRPLDFPHAVLNYAKPLFFKLEV"

/label=STH csm4

/ApEinfo_fwdcolor=#ff8000

/ApEinfo_revcolor=#ff8000

/ApEinfo_graphicformat=arrow_data {{0 1 2 0 0 -1} {} 0}

width 5 offset 0

promoter 7550..7568

/note="T7 promoter"

/note="promoter for bacteriophage T7 RNA polymerase"

/label=T7 promoter(6)

/ApEinfo_label=T7 promoter

/ApEinfo_fwdcolor=#00ff00

/ApEinfo_revcolor=#00ff00

/ApEinfo_graphicformat=arrow_data {{0 1 2 0 0 -1} {} 0}

width 5 offset 0

CDS 7595..8701

/codon_start=1

/note="STH csm5"

/translation="MGHHHHHHSGGLKNDYRTFKLSLLTLAPIHIGNGEKYTSREFIYENKKFYFPDMGKFYNKMVEKRLAEKFEAFLIQTRPNARNNRLISFLNDNRIAERSFGGYSISETGLESDRNPNSAGAINEVNKFIRDAFGNPYIPGSSLKGAIRTILMNTTPKWNNENAVNDFGRFPKENKNLIPWGPKKGKEYDDLFNAIRVSDSKPFDNKRLILVQKWDYSAKTNKAKPLPLYRESISPLTKIEFEITTTTDEAGRLIEELGKRAQAFYKDYKAFFLSEFPDDKIQANLQYPIYLGAGSGAWTKTLFKQADGILQRRYSRMKTKMVKKGVLKLTKAPLKIVKIPSGNHSLIKNHESFYEMGKANFMIKEIDK"

/label=STH csm5

/ApEinfo_fwdcolor=#ff8000

/ApEinfo_revcolor=#ff8000

/ApEinfo_graphicformat=arrow_data {{0 1 2 0 0 -1} {} 0}

width 5 offset 0

promoter 8777..8795

/note="T7 promoter"

/note="promoter for bacteriophage T7 RNA polymerase"

/label=T7 promoter(7)

/ApEinfo_label=T7 promoter

/ApEinfo_fwdcolor=#00ff00

/ApEinfo_revcolor=#00ff00

/ApEinfo_graphicformat=arrow_data {{0 1 2 0 0 -1} {} 0}

width 5 offset 0

CDS 8822..10015

/codon_start=1

/note="STH csm6"

/translation="MEVQVNLRRKNMRVLISAVGDTDPFRNFHDGALIHIARKYRPEKVILIFSEHTAKKQGNIEKALFSIAPNYEPELIIHDPIISDNEVHIFDVMFQRFSDILQEYYTKEDEFILNLSSATPQIKSALFVINRLNGINVKAVQVSSPEHASNENIGHDNDENIDELIEVNKDNKVNFIDRTIEDNAEKFSQALLKKTARDFIEKFDYKAALDILDQLSDFPNLKSVREEIRDVVNCLSKQDVPKGLRHKKLKEEEQKILSAYLTIELQRERGNVSESFIRIKNLTEFILEDYIEKRYPGLIDEYCEDIQKYYLSLFDYSKLLKATKEFKLKRTIAPIIDMNSSRNKVAHSLSPLDSDAVKQLGIAMKTLKTLVREQYHFSQSDFNFYHDLNKILLTKLN"

/label=STH csm6

/ApEinfo_fwdcolor=#ff8000

/ApEinfo_revcolor=#ff8000

/ApEinfo_graphicformat=arrow_data {{0 1 2 0 0 -1} {} 0}

width 5 offset 0

terminator 10042..10088

/note="T7 terminator"

/note="transcription terminator for bacteriophage T7 RNA

polymerase"

/label=T7 terminator(1)

/ApEinfo_label=T7 terminator

/ApEinfo_fwdcolor=#ff0000

/ApEinfo_revcolor=#ff0000

/ApEinfo_graphicformat=arrow_data {{0 1 2 0 0 -1} {} 0}

width 5 offset 0

CDS complement(join(10245..10685,1..219))

/codon_start=1

/gene="cat"

/product="chloramphenicol acetyltransferase"

/note="CmR"

/note="confers resistance to chloramphenicol"

/translation="MEKKITGYTTVDISQWHRKEHFEAFQSVAQCTYNQTVQLDITAFLKTVKKNKHKFYPAFIHILARLMNAHPEFRMAMKDGELVIWDSVHPCYTVFHEQTETFSSLWSEYHDDFRQFLHIYSQDVACYGENLAYFPKGFIENMFFVSANPWVSFTSFDLNVANMDNFFAPVFTMGKYYTQGDKVLMPLAIQVHHAVCDGFHVGRMLNELQQYCDEWQGGA"

/label=chloramphenicol acetyltransferase

/ApEinfo_fwdcolor=#ffcc66

/ApEinfo_revcolor=#ffcc66

/ApEinfo_graphicformat=arrow_data {{0 1 2 0 0 -1} {} 0}

width 5 offset 0

ORIGIN

1 gaactccggg tgagcattca tcaggcgggc aagaatgtga ataaaggccg gataaaactt

61 gtgcttattt ttctttacgg tctttaaaaa ggccgtaata tccagctgaa cggtctggtt

121 ataggtacat tgagcaactg actgaaatgc ctcaaaatgt tctttacgat gccattggga

181 tatatcaacg gtggtatatc cagtgatttt tttctccatt ttagcttcct tagctcctga

241 aaatctcgat aactcaaaaa atacgcccgg tagtgatctt atttcattat ggtgaaagtt

301 ggaacctctt acgtgccgat caaggtctca ttttcgccaa aagttggccc agggcttccc

361 ggtatcaaca gggacaccag gatttattta ttctgcgaag tgatcttccg tcacaggtat

421 ttattcggcg caaagtgcgt cgggtgatgc tgccaactta ctgatttagt gtatgatggt

481 gtttttgagg tgctccagtg gcttctgttt ctatcagctg tccctcctgt tcagctactg

541 acggggtggt gcgtaacggc aaaagcaccg ccggacatca gcgctagcgg agtgtatact

601 ggcttactat gttggcactg atgagggtgt cagtgaagtg cttcatgtgg caggagaaaa

661 aaggctgcac cggtgcgtca gcagaatatg tgatacagga tatattccgc ttcctcgctc

721 actgactcgc tacgctcggt cgttcgactg cggcgagcgg aaatggctta cgaacggggc

781 ggagatttcc tggaagatgc caggaagata cttaacaggg aagtgagagg gccgcggcaa

841 agccgttttt ccataggctc cgcccccctg acaagcatca cgaaatctga cgctcaaatc

901 agtggtggcg aaacccgaca ggactataaa gataccaggc gtttccccct ggcggctccc

961 tcgtgcgctc tcctgttcct gcctttcggt ttaccggtgt cattccgctg ttatggccgc

1021 gtttgtctca ttccacgcct gacactcagt tccgggtagg cagttcgctc caagctggac

1081 tgtatgcacg aaccccccgt tcagtccgac cgctgcgcct tatccggtaa ctatcgtctt

1141 gagtccaacc cggaaagaca tgcaaaagca ccactggcag cagccactgg taattgattt

1201 agaggagtta gtcttgaagt catgcgccgg ttaaggctaa actgaaagga caagttttgg

1261 tgactgcgct cctccaagcc agttacctcg gttcaaagag ttggtagctc agagaacctt

1321 cgaaaaaccg ccctgcaagg cggttttttc gttttcagag caagagatta cgcgcagacc

1381 aaaacgatct caagaagatc atcttattaa tcagataaaa tatttctaga tttcagtgca

1441 atttatctct tcaaatgtag cacctgaagt cagccccata cgatataagt tgtaattctc

1501 atgtttgaca gccttctaat acgactcact atagggagaa taaaggaggt aaataatgaa

1561 aaaactggtg ttcaccttca aacgcattga tcatccggca caggatctgg cagttaaatt

1621 tcatggtttt ctgatggaac agctggatag cgattatgtg gattatctgc atcagcagca

1681 gaccaatccg tatgcaacca aagttattca gggcaaagaa aatacccagt gggttgttca

1741 tctgctgacc gatgatcacg aagataaagt ttttatgacc ctgctgcaga tcaaagaagt

1801 tagcctgaac gatctgccga aactgagcgt tgaaaaagtg gaaattcaag aactgggtgc

1861 agataaactg ctggaaatct ttaatagcga agagaaccag acctacttca gcattatctt

1921 tgaaaccccg accggtttta aaagccaggg tagctatgtt atttttccga gcatgcgtct

1981 gatttttcag agcctgatgc aaaaatatgg tcgcctggtt gaaaatcagc cggaaattga

2041 agaagatacc ctggattacc tgagcgaaca tagcaccatt accaattatc gtctggaaac

2101 cagctatttt cgtgtgcatc gtcagcgtat tccggcattt cgtggtaaac tgacctttaa

2161 agtgcagggt gcaaaaaccc tgaaagccta tgttaaaatg ctgctgacct ttggtgaata

2221 tagcggtctg ggtatgaaaa ccagcctggg tatgggtggt attaaactgg aagaacgcaa

2281 agactaaaag ctttcctgtg agcagcgaaa gcctagcata accccttggg gcctctaaac

2341 gggtcttgag gggttttttg ttatacgcga gatacactga atcacttgca tagctgcgta

2401 tggaggaagc aactcttgag tgttaatatg ttgacccctg tattagggat gcgggtagta

2461 gatgtgggca gagacacccc cagatcttaa tacgactcac tatagggaga taatacgact

2521 cactataggg aatacaagct acttgttctt tttgcaccat gggatataaa cctaattacc

2581 tcgagagggg acggaaacct tgattctaac gctacttcta aataagcgtt agcaatggat

2641 ataaacctaa ttacctcgag aggggacgga aacctcgagg ctgtggtcta gacattccat

2701 acatatcggg ggggtagggg ttttttgtgt gcctctagtg gctggctaag aataatacga

2761 ctcactatag ggagaggatc cataaaggag gtaaataatg aaaaaagaga aaatcgacct

2821 gttctatggt gcactgctgc atgatattgg taaagttatt cagcgtgcaa ccggtgaacg

2881 taaaaaacat gcactggttg gtgcagattg gtttgatgaa attgcagata atcaggtgat

2941 cagcgatcag attcgttatc acatggcaaa ctatcagagc gataaactgg gtaatgatca

3001 tctggcctat attacctata tcgccgataa tattgcaagc ggtgttgatc gtcgtcagag

3061 caatgaagaa agtgatgaag atgcaagcgc caaaatttgg gatacctata ccaatcaggc

3121 cgatatcttt aatgtttttg gtgcccagac cgataaacgc tatttcaaac cgaccgttct

3181 gaacctgaaa agcaaaccga attttgcaag cgcaacctat gaaccgttta gcaaaggtga

3241 ttatgcagca attgcaaccc gcattaaaaa cgaactggcc gaatttgaat ttaaccaggc

3301 acagattgat agcctgctga acctgtttga agcaattctg agctttgttc cgagcagcac

3361 caatagcaaa gaaattgccg atattagcct ggcagaacat agccgtctga ccgcagcatt

3421 tgcactggca atttatgatt acctggaaga taaaggtcgc cacaactata aagaggacct

3481 gtttaccaaa gcaagcgcct tttatgaaga agaagcattt ctgctggcat cctttgatct

3541 gagcggtatt caggatttca tctataacat tgcaaccagc ggtgcagcaa aacagctgaa

3601 agcacgtagc ctgtatctgg attttatgag cgaatatatt gccgacagcc tgctggacaa

3661 actgggtctg aatcgtgcaa atctgctgta tgttggtggt ggtcatgcat attttgttct

3721 ggcaaatacc gaaaaaaccg ttgaaaccct ggtgcagttt gagaaagatt ttaatcagtt

3781 cctgctggcc aattttcaga cccgtctgta tgtggcattt ggttggggta gctttgcagc

3841 caaagatatt atgagtgaac tgaatagtcc ggaaagctat cgtcagattt atcagaaagc

3901 aagccgtatg attagcgaga aaaaaatctc ccgttatgat tatcgtaccc tgatgctgct

3961 gaatcgcggt ggtaaaagca gcgaacgtga atgtgaaatt tgtcatagcg ttgaaaatct

4021 ggtgagctat cacgatcaga aagtttgtga tatttgccgt ggtctgtatc agtttagtaa

4081 agaaatcgcc cacgatcact ttatcatcac cgaaaatgaa ggtctgccga ttggtccgaa

4141 tgcatgtctg aaaggtgttg catttgaaaa actgagccaa gaaagtttca gccgtgtgta

4201 tgtgaaaaac gattataaag caggcaccat caaagccacc catgtttttg ttggtgatta

4261 tcagtgtgac gagatccata aatatgcagc cctgagcaaa aatgaagatg gcctgggtat

4321 taaacgtctg gcagttgttc gtctggatgt tgatgatctg ggtgcagcat ttatggcagg

4381 ttttagccgt cagggtaatg gtcagtatag caccctgagc cgtagcgcca cctttagccg

4441 tagcatgagc ctgttcttta aagtgtatat taaccagttc gccagcgata aaaaactgtc

4501 cattatctat gccggtggtg atgatgtttt tgcaattggt agctggcagg atattattgc

4561 ctttaccgtt gaactgcgcc agaactttat caaatggacc aatggtaaac tgaccctgag

4621 cgcaggtatt ggtctgtttg ccgataaaac cccgattagc ctgatggcac atcagacagg

4681 tgaactggaa gaggcagcaa aaggtaatga aaaagattcc attagcctgt tcagcagcga

4741 ttacaccttt aaattcgatc gctttattac caacgtgtat gacgacaaac tggaacaaat

4801 ccgctatttt ttcaaccatc aggatgaacg cggtaaaaac ttcatctaca aactgattga

4861 actgctgcgc aattatgaga gcgaagaaaa aatgaatgtt gcccgtctgg catattatct

4921 gacacgtctg gaagaactga cggataaaga tgaacgtgac aaattcaaac aattcaaaaa

4981 actgttcttc aaatggtaca ccaacaatga atccgatcgt aaagaagcag aactggccct

5041 gctgctgtac gtttatgaaa ttcgtaaaga ttaacatatg gctgcgtggt caaatgtgcg

5101 taccctaacc ccttccccgg tcaatcgggg cggatggggt tttttgtgcg tacttcatta

5161 tgtatattaa tacgactcac tatagggaga agatctataa aggaggtaaa taatggcaat

5221 tttaactgat gagaattatg tagataaagc agaacgtgct atttcattac ttgaaaagga

5281 taataaaggg aattatttac taacaacgtc acaaattaga aagttattat ccttatgtag

5341 ttctttatat gatagaagta aagaaagaaa gtttgatgaa ttaataaatg atgtttccta

5401 tttaagagta caattcgtct atcagtctgg aagaaactct gtaagagtta atagacagac

5461 gttcttccct gttaaagatt tggtagaaaa aggtcaaatt cttgaagcgc ttaaggaaat

5521 caaagataga gagacacttc aaagattttg tagatatatg gaagcattag tagcctattt

5581 caagttttat ggaggtaaag attaaacgcg tgctgcgtgg tcaaatgtgc gtagaccaac

5641 cccttgcggc ctcaatcggg ggggatgggg ttttttgtca ggcaagtctc agctggttta

5701 atacgactca ctatagggag agaattcata aaggaggtaa ataatgggtc accaccatca

5761 tcaccatagc ggtggattga ccttcgccaa aatcaaattt agcgcacaga ttcgtctgga

5821 aaccggtctg catattggtg gtagtgatgc atttgcagca attggtgcca ttgatagtcc

5881 ggttattaaa gatccgatta ccaacctgcc gattattccg ggtagcagcc tgaaaggtaa

5941 aatgcgtacc ctgctggcaa aagtgtataa tgaaaaagtt gccgaaaaac cgtccgatga

6001 tagcgatatt ctgagccgtc tgtttggtaa tagcaaagac aaacgcttta aaatgggtcg

6061 cctgattttt cgtgatgcct ttctgagcaa tgcagatgaa ctggatagcc tgggtgttcg

6121 tagctatacc gaagtgaaat ttgaaaacac cattgatcgc attaccgcag aagcaaatcc

6181 gcgtcagatt gaacgtgcaa ttcgtaatag cacctttgat ttcgaactga tctatgagat

6241 caccgatgaa aatgaaaacc aggtggaaga ggatttcaaa gtgattcgtg atggtctgaa

6301 actgctggaa ctggattatc tgggtggtag cggtagccgt ggttatggta aagttgcatt

6361 tgaaaatctg aaagccacca ccgtgtttgg caattatgat gttaaaaccc tgaatgaact

6421 gctgacagcc gaagtttaaa agcttacctg gagatcaagg agattactct aaccccatcg

6481 gccgtcttag gggttttttg tcctgtgtta gctggagggt ataatacgac tcactatagg

6541 gagacccggg ataaaggagg taaataatga cctacaaact gtacattatg acctttcaga

6601 atgcccattt tggtagcggc accctggata gcagcaaact gacctttagc gcagatcgca

6661 tttttagcgc actggttctg gaagcactga aaatgggtaa actggatgca tttctggcag

6721 aagcaaatca ggataaattc accctgaccg atgcctttcc gtttcagttt ggtccgtttc

6781 tgccgaaacc gattggttat ccgaaacatg atcagattga tcagagcgtg gatgttaaag

6841 aagttcgtcg tcaggcaaaa ctgagcaaaa aactgcaatt tctggccctg gaaaacgtgg

6901 atgattatct gaatggtgaa ctgtttgaaa acgaagatca tgccgttatt gataccgtga

6961 ccaaaaatca gccgcataaa gatggtaatc tgtatcaggt tgccaccacc cgttttagca

7021 atgataccag cctgtatgtt attgccaatg aaagcgatct gctgaatgaa ctgatgagca

7081 gcctgcagta tagcggtctg ggtggtaaac gtagcagcgg ttttggtcgt tttgaactgg

7141 atattcagaa tattccgctg gaactgagcg atcgtctgac aaaaaatcat agcgataaag

7201 ttatgagcct gaccaccgca ctgccggttg atgccgatct ggaagaggca atggaagatg

7261 gtcattatct gctgaccaaa agcagtggtt ttgcatttag ccatgccacc aatgaaaatt

7321 atcgtaaaca ggatctgtat aaattcgcaa gcggtagcac ctttagcaaa acctttgaag

7381 gtcagattgt tgatgttcgt ccgctggatt ttccgcatgc agttctgaat tatgccaaac

7441 cgctgttttt caaactggaa gtgtaatcat gatttcttgt cgaactggac agtagcagaa

7501 ccgctaacgg gggcgaaggg gttttttgtg acatacgagc tgattgaact aatacgactc

7561 actataggga gaggtaccat aaaggaggta aataatgggt caccaccatc atcaccatag

7621 cggtggattg aaaaacgatt accgcacctt taaactgagc ctgctgaccc tggcaccgat

7681 tcatattggt aatggtgaga aatataccag ccgtgagttt atctatgaga acaaaaaatt

7741 ctattttccg gatatgggca aattctataa caaaatggtg gaaaaacgcc tggccgaaaa

7801 atttgaagca tttctgattc agacccgtcc gaatgcacgt aataatcgtc tgattagctt

7861 tctgaacgat aaccgtattg cagaacgtag ctttggtggt tatagcatta gcgaaaccgg

7921 tctggaaagc gatcgtaatc cgaatagtgc cggtgcaatt aatgaagtga acaaattcat

7981 tcgtgatgcc tttggcaatc cgtatattcc gggtagcagc ctgaaaggtg caattcgtac

8041 cattctgatg aataccaccc cgaaatggaa taatgaaaac gccgttaatg attttggtcg

8101 ctttccgaaa gaaaacaaaa atctgattcc gtggggtccg aaaaaaggca aagaatatga

8161 tgacctgttt aacgccattc gtgttagcga tagcaaaccg tttgataaca aacgtctgat

8221 tctggtgcag aaatgggatt atagcgccaa aaccaataaa gcaaaaccgc tgccgctgta

8281 tcgtgaaagc attagtccgc tgaccaaaat tgaatttgaa attaccacca ccaccgatga

8341 agcaggtcgc ctgattgaag aactgggtaa acgtgcacag gcattctata aagattacaa

8401 agcgtttttt ctgagcgagt tcccggatga taaaattcag gcaaatctgc agtatccgat

8461 ttatctgggt gcaggtagcg gtgcatggac caaaaccctg ttcaaacagg cagatggtat

8521 tctgcagcgt cgttatagcc gtatgaaaac gaaaatggtt aaaaaaggcg tgctgaaact

8581 gaccaaagca ccgctgaaaa ttgtgaaaat tccgagcggt aatcacagcc tgattaaaaa

8641 ccatgagagc ttttatgaaa tgggcaaagc caactttatg atcaaagaaa tcgataaata

8701 acctaggcgc ttcaacggaa cggatcttac atatcggggg ggtaggggtt ttttgtctcg

8761 gagaccaagt agggcataat acgactcact atagggagac tatggataaa ggaggtaaat

8821 aatggaagtc caagtaaatt taaggagaaa aaatatgcgt gttctgatta gcgcagttgg

8881 tgataccgat ccgtttcgta attttcacga tggtgcactg attcatatcg cacgtaaata

8941 tcgtccggaa aaagtgattc tgatctttag cgaacacacc gcaaaaaaac agggcaatat

9001 tgaaaaagcc ctgtttagca ttgcaccgaa ttatgaaccg gaactgatta tccatgatcc

9061 gattattagc gataacgagg tgcatatctt tgacgtgatg tttcagcgtt ttagcgatat

9121 cctgcaagag tattatacca aagaggatga gtttatcctg aatctgagca gcgcaacacc

9181 gcagattaaa agcgcactgt ttgttattaa tcgcctgaat ggcattaatg tgaaagcagt

9241 tcaggttagc agtccggaac atgcaagcaa tgaaaatatt ggccatgata acgatgagaa

9301 catcgatgaa ctgatcgagg tgaacaaaga caacaaagtg aactttattg atcgcaccat

9361 tgaagataac gccgaaaaat tcagccaggc actgctgaaa aaaaccgcac gtgattttat

9421 cgagaaattt gactataaag cagccctgga tattctggat cagctgagcg attttccgaa

9481 tctgaaaagc gttcgtgaag aaattcgtga tgttgttaat tgcctgagca aacaggatgt

9541 tccgaaaggt ctgcgtcata aaaaactgaa agaagaagaa cagaaaatcc tgagcgcata

9601 tctgaccatt gaactgcagc gtgaacgtgg taatgttagc gaaagtttta tccgcattaa

9661 aaacctgacc gagttcatcc tggaagatta tatcgaaaaa cgttatccgg gtctgatcga

9721 tgaatattgc gaagatatcc agaaatacta cctgagcctg ttcgattata gcaaactgct

9781 gaaagccacc aaagaattta aactgaaacg tacgatcgca ccgatcattg atatgaatag

9841 cagccgtaat aaagttgccc atagcctgag tccgctggat agtgatgcag ttaaacagct

9901 gggtattgcc atgaaaaccc tgaaaacact ggttcgtgaa cagtatcatt ttagccagag

9961 cgatttcaac ttctatcacg acctgaacaa aatcctgctg accaaactga actaactcga

10021 gaggttacag cctgcataat gtagcataac cccttggggc ctctaaacgg gtcttgaggg

10081 gttttttgtg cctatagttt gaagcagaat cgaatttctg ccattcatcc gcttattatc

10141 acttattcag gcgtagcacc aggcgtttaa gggcaccaat aactgcctta caaaaaaccc

10201 ctagccgccc gataagagcg ggctaggggt tcgagtaaaa aaaattacgc cccgccctgc

10261 cactcatcgc agtactgttg taattcatta agcattctgc cgacatggaa gccatcacag

10321 acggcatgat gaacctgaat cgccagcggc atcagcacct tgtcgccttg cgtataatat

10381 ttgcccatcg tgaaaacggg ggcgaagaag ttgtccatat tggccacgtt taaatcaaaa

10441 ctggtgaaac tcacccaggg attggctgaa acgaaaaaca tattctcaat aaacccttta

10501 gggaaatagg ccaggttttc accgtaacac gccacatctt gcgaatatat gtgtagaaac

10561 tgccggaaat cgtcgtggta ttcactccag agcgatgaaa acgtttcagt ttgctcatgg

10621 aaaacggtgt aacaagggtg aacactatcc catatcacca gctcaccgtc tttcattgcc

10681 atacg

//

**Figure D in S1 file. *Lactococcus lactis* forward orientation target (LLA-f) plasmid.** LLA crRNA target sequence is in the forward orientation (yellow, LLA-f target), relative to the trc promoter (blue). The target sequence is inserted between the IPTG-inducible lac operator (teal) and rrnB T1/T2 terminators (red) of the pTrcHis-TOPO plasmid, which carries the colE1 origin of replication (colE1 ori, olive color) and ampicillin resistance marker (purple). Green arrows represent AmpR and lacIq transcriptional promoters. Plasmid map illustration made using the SnapGene program. Plasmid sequence information is in GenBank format, using ApE program.


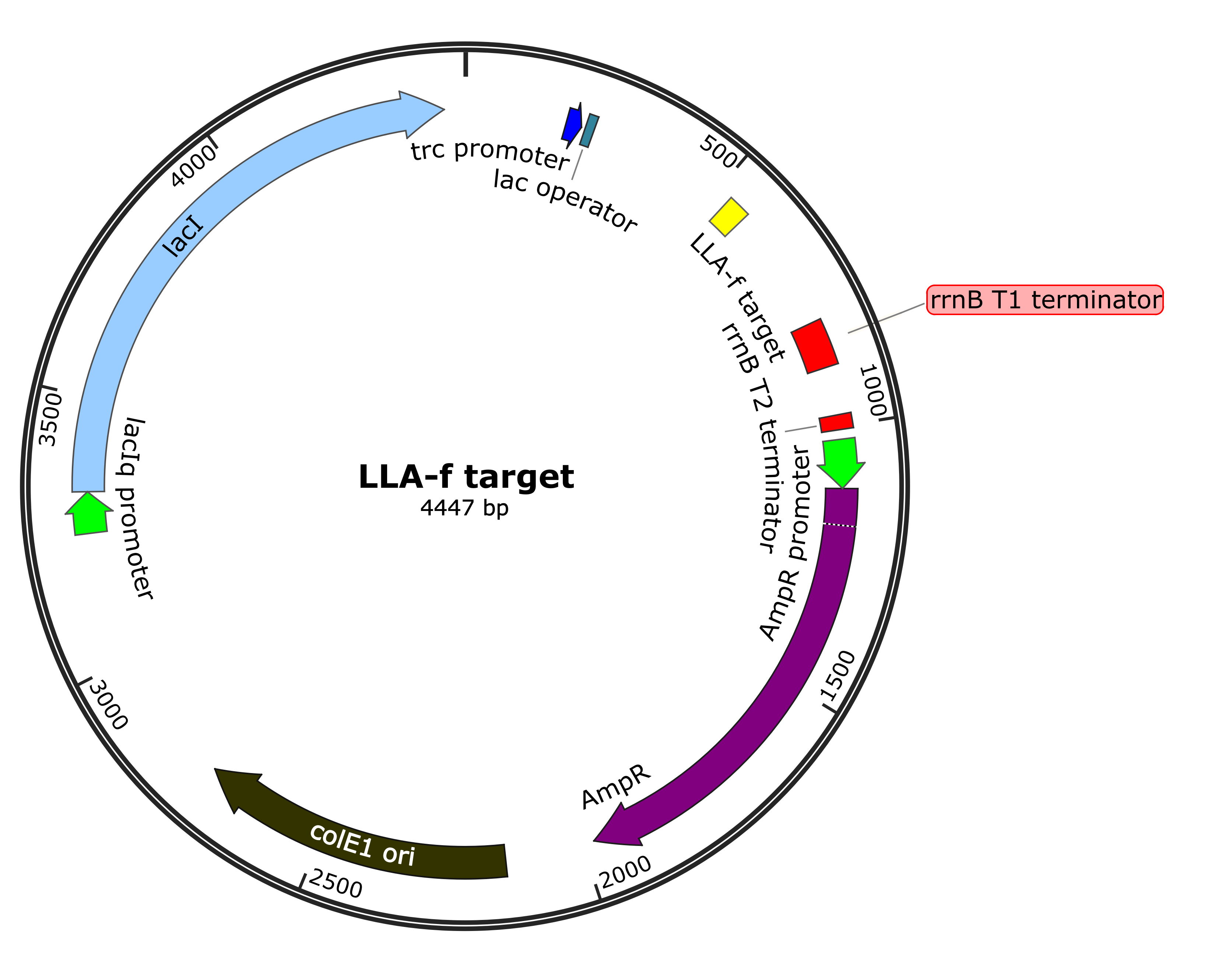


LOCUS LLA_f_target 4447 bp ds-DNA circular 22-FEB-2017

DEFINITION synthetic circular DNA

ACCESSION .

VERSION .

KEYWORDS LLA-f target

SOURCE synthetic DNA construct

ORGANISM synthetic DNA construct

REFERENCE 1 (bases 1 to 4447)

AUTHORS .

TITLE Direct Submission

JOURNAL Exported Sunday, Feb 19, 2017 from SnapGene 3.3.3

http://www.snapgene.com

COMMENT

COMMENT

COMMENT ApEinfo:methylated:1

FEATURES Location/Qualifiers

promoter 193..222

/note="trc promoter"

/note="strong E. coli promoter; hybrid between the trp and

lac UV5 promoters"

/label=trc promoter

/ApEinfo_fwdcolor=#0000ff

/ApEinfo_revcolor=#0000ff

/ApEinfo_graphicformat=arrow_data {{0 1 2 0 0 -1} {} 0}

width 5 offset 0

protein_bind 230..246

/bound_moiety="lac repressor encoded by lacI"

/note="lac operator"

/note="The lac repressor binds to the lac operator to

inhibit transcription in E. coli. This inhibition can be

relieved by adding lactose or

isopropyl-beta-D-thiogalactopyranoside (IPTG)."

/label=lac operator

/ApEinfo_fwdcolor=#008080

/ApEinfo_revcolor=#008080

/ApEinfo_graphicformat=arrow_data {{0 1 2 0 0 -1} {} 0}

width 5 offset 0

misc_feature 528..570

/note="LLA-f target"

/label=LLA-f target

/ApEinfo_fwdcolor=#ffff00

/ApEinfo_revcolor=#ffff00

/ApEinfo_graphicformat=arrow_data {{0 1 2 0 0 -1} {} 0}

width 5 offset 0

terminator 801..887

/gene="Escherichia coli rrnB"

/note="rrnB T1 terminator"

/note="transcription terminator T1 from the E. coli rrnB

gene"

/label=rrnB T1 terminator

/ApEinfo_fwdcolor=#ff0000

/ApEinfo_revcolor=#ff0000

/ApEinfo_graphicformat=arrow_data {{0 1 2 0 0 -1} {} 0}

width 5 offset 0

terminator 979..1006

/note="rrnB T2 terminator"

/note="transcription terminator T2 from the E. coli rrnB

gene"

/label=rrnB T2 terminator

/ApEinfo_fwdcolor=#ff0000

/ApEinfo_revcolor=#ff0000

/ApEinfo_graphicformat=arrow_data {{0 1 2 0 0 -1} {} 0}

width 5 offset 0

promoter 1025..1116

/gene="bla"

/note="AmpR promoter"

/label=AmpR promoter

/ApEinfo_fwdcolor=#00ff00

/ApEinfo_revcolor=#00ff00

/ApEinfo_graphicformat=arrow_data {{0 1 2 0 0 -1} {} 0}

width 5 offset 0

CDS 1117..1977

/codon_start=1

/gene="bla"

/product="beta-lactamase"

/note="AmpR"

/note="confers resistance to ampicillin, carbenicillin,

and related antibiotics"

/translation="MSIQHFRVALIPFFAAFCLPVFAHPETLVKVKDAEDQLGARVGYIELDLNSGKILESFRPEERFPMMSTFKVLLCGAVLSRVDAGQEQLGRRIHYSQNDLVEYSPVTEKHLTDGMTVRELCSAAITMSDNTAANLLLTTIGGPKELTAFLHNMGDHVTRLDRWEPELNEAIPNDERDTTMPVAMATTLRKLLTGELLTLASRQQLIDWMEADKVAGPLLRSALPAGWFIADKSGAGERGSRGIIAALGPDGKPSRIVVIYTTGSQATMDERNRQIAEIGASLIKHW"

/label=AmpR

/ApEinfo_fwdcolor=#800080

/ApEinfo_revcolor=#800080

/ApEinfo_graphicformat=arrow_data {{0 1 2 0 0 -1} {} 0}

width 5 offset 0

rep_origin 2148..2736

/direction=RIGHT

/note="colE1 ori"

/note="high-copy-number ColE1/pMB1/pBR322/pUC origin of

replication"

/label=colE1 ori

/ApEinfo_fwdcolor=#808000

/ApEinfo_revcolor=#808000

/ApEinfo_graphicformat=arrow_data {{0 1 2 0 0 -1} {} 0}

width 5 offset 0

promoter 3248..3325

/gene="lacI (mutant)"

/note="lacIq promoter"

/note="In the lacIq allele, a single base change in the

promoter boosts expression of the lacI gene about

10-fold."

/label=lacIq promoter

/ApEinfo_fwdcolor=#00ff00

/ApEinfo_revcolor=#00ff00

/ApEinfo_graphicformat=arrow_data {{0 1 2 0 0 -1} {} 0}

width 5 offset 0

CDS 3326..4408

/codon_start=1

/gene="lacI"

/product="lac repressor"

/note="lacI"

/note="The lac repressor binds to the lac operator to

inhibit transcription in E. coli. This inhibition can be

relieved by adding lactose or

isopropyl-beta-D-thiogalactopyranoside (IPTG)."

/translation="MKPVTLYDVAEYAGVSYQTVSRVVNQASHVSAKTREKVEAAMAELNYIPNRVAQQLAGKQSLLIGVATSSLALHAPSQIVAAIKSRADQLGASVVVSMVERSGVEACKAAVHNLLAQRVSGLIINYPLDDQDAIAVEAACTNVPALFLDVSDQTPINSIIFSHEDGTRLGVEHLVALGHQQIALLAGPLSSVSARLRLAGWHKYLTRNQIQPIAEREGDWSAMSGFQQTMQMLNEGIVPTAMLVANDQMALGAMRAITESGLRVGADISVVGYDDTEDSSCYIPPLTTIKQDFRLLGQTSVDRLLQLSQGQAVKGNQLLPVSLVKRKTTLAPNTQTASPRALADSLMQLARQVSRLESGQ"

/label=lacI

/ApEinfo_fwdcolor=#66ccff

/ApEinfo_revcolor=#66ccff

/ApEinfo_graphicformat=arrow_data {{0 1 2 0 0 -1} {} 0}

width 5 offset 0

ORIGIN

1 gtttgacagc ttatcatcga ctgcacggtg caccaatgct tctggcgtca ggcagccatc

61 ggaagctgtg gtatggctgt gcaggtcgta aatcactgca taattcgtgt cgctcaaggc

121 gcactcccgt tctggataat gttttttgcg ccgacatcat aacggttctg gcaaatattc

181 tgaaatgagc tgttgacaat taatcatccg gctcgtataa tgtgtggaat tgtgagcgga

241 taacaatttc acacaggaaa cagcgccgct gagaaaaagc gaagcggcac tgctctttaa

301 caatttatca gacaatctgt gtgggcactc gaccggaatt atcgattaac tttattatta

361 aaaattaaag aggtatatat taatgtatcg attaaataag gaggaataaa ccatgggggg

421 ttctcatcat catcatcatc atggtatggc tagcatgact ggtggacagc aaatgggtcg

481 ggatctgtac gacgatgacg ataaggatcc aacccttttc caagctttcc gagggagttg

541 aagcttggtt caaagaacgt atcaagagca gcatgcttcc aaggcgaatt cgaagcttgg

601 ctgttttggc ggatgagaga agattttcag cctgatacag attaaatcag aacgcagaag

661 cggtctgata aaacagaatt tgcctggcgg cagtagcgcg gtggtcccac ctgaccccat

721 gccgaactca gaagtgaaac gccgtagcgc cgatggtagt gtggggtctc cccatgcgag

781 agtagggaac tgccaggcat caaataaaac gaaaggctca gtcgaaagac tgggcctttc

841 gttttatctg ttgtttgtcg gtgaacgctc tcctgagtag gacaaatccg ccgggagcgg

901 atttgaacgt tgcgaagcaa cggcccggag ggtggcgggc aggacgcccg ccataaactg

961 ccaggcatca aattaagcag aaggccatcc tgacggatgg cctttttgcg tttctacaaa

1021 ctcttttgtt tatttttcta aatacattca aatatgtatc cgctcatgag acaataaccc

1081 tgataaatgc ttcaataata ttgaaaaagg aagagtatga gtattcaaca tttccgtgtc

1141 gcccttattc ccttttttgc ggcattttgc cttcctgttt ttgctcaccc agaaacgctg

1201 gtgaaagtaa aagatgctga agatcagttg ggtgcacgag tgggttacat cgaactggat

1261 ctcaacagcg gtaagatcct tgagagtttt cgccccgaag aacgttttcc aatgatgagc

1321 acttttaaag ttctgctatg tggcgcggta ttatcccgtg ttgacgccgg gcaagagcaa

1381 ctcggtcgcc gcatacacta ttctcagaat gacttggttg agtactcacc agtcacagaa

1441 aagcatctta cggatggcat gacagtaaga gaattatgca gtgctgccat aaccatgagt

1501 gataacactg cggccaactt acttctgaca acgatcggag gaccgaagga gctaaccgct

1561 tttttgcaca acatggggga tcatgtaact cgccttgatc gttgggaacc ggagctgaat

1621 gaagccatac caaacgacga gcgtgacacc acgatgcctg tagcaatggc aacaacgttg

1681 cgcaaactat taactggcga actacttact ctagcttccc ggcaacaatt aatagactgg

1741 atggaggcgg ataaagttgc aggaccactt ctgcgctcgg cccttccggc tggctggttt

1801 attgctgata aatctggagc cggtgagcgt gggtctcgcg gtatcattgc agcactgggg

1861 ccagatggta agccctcccg tatcgtagtt atctacacga cggggagtca ggcaactatg

1921 gatgaacgaa atagacagat cgctgagata ggtgcctcac tgattaagca ttggtaactg

1981 tcagaccaag tttactcata tatactttag attgatttaa aacttcattt ttaatttaaa

2041 aggatctagg tgaagatcct ttttgataat ctcatgacca aaatccctta acgtgagttt

2101 tcgttccact gagcgtcaga ccccgtagaa aagatcaaag gatcttcttg agatcctttt

2161 tttctgcgcg taatctgctg cttgcaaaca aaaaaaccac cgctaccagc ggtggtttgt

2221 ttgccggatc aagagctacc aactcttttt ccgaaggtaa ctggcttcag cagagcgcag

2281 ataccaaata ctgtccttct agtgtagccg tagttaggcc accacttcaa gaactctgta

2341 gcaccgccta catacctcgc tctgctaatc ctgttaccag tggctgctgc cagtggcgat

2401 aagtcgtgtc ttaccgggtt ggactcaaga cgatagttac cggataaggc gcagcggtcg

2461 ggctgaacgg ggggttcgtg cacacagccc agcttggagc gaacgaccta caccgaactg

2521 agatacctac agcgtgagct atgagaaagc gccacgcttc ccgaagggag aaaggcggac

2581 aggtatccgg taagcggcag ggtcggaaca ggagagcgca cgagggagct tccaggggga

2641 aacgcctggt atctttatag tcctgtcggg tttcgccacc tctgacttga gcgtcgattt

2701 ttgtgatgct cgtcaggggg gcggagccta tggaaaaacg ccagcaacgc ggccttttta

2761 cggttcctgg ccttttgctg gccttttgct cacatgttct ttcctgcgtt atcccctgat

2821 tctgtggata accgtattac cgcctttgag tgagctgata ccgctcgccg cagccgaacg

2881 accgagcgca gcgagtcagt gagcgaggaa gcggaagagc gcctgatgcg gtattttctc

2941 cttacgcatc tgtgcggtat ttcacaccgc atatggtgca ctctcagtac aatctgctct

3001 gatgccgcat agttaagcca gtatacactc cgctatcgct acgtgactgg gtcatggctg

3061 cgccccgaca cccgccaaca cccgctgacg cgccctgacg ggcttgtctg ctcccggcat

3121 ccgcttacag acaagctgtg accgtctccg ggagctgcat gtgtcagagg ttttcaccgt

3181 catcaccgaa acgcgcgagg cagcagatca attcgcgcgc gaaggcgaag cggcatgcat

3241 ttacgttgac accatcgaat ggtgcaaaac ctttcgcggt atggcatgat agcgcccgga

3301 agagagtcaa ttcagggtgg tgaatgtgaa accagtaacg ttatacgatg tcgcagagta

3361 tgccggtgtc tcttatcaga ccgtttcccg cgtggtgaac caggccagcc acgtttctgc

3421 gaaaacgcgg gaaaaagtgg aagcggcgat ggcggagctg aattacattc ccaaccgcgt

3481 ggcacaacaa ctggcgggca aacagtcgtt gctgattggc gttgccacct ccagtctggc

3541 cctgcacgcg ccgtcgcaaa ttgtcgcggc gattaaatct cgcgccgatc aactgggtgc

3601 cagcgtggtg gtgtcgatgg tagaacgaag cggcgtcgaa gcctgtaaag cggcggtgca

3661 caatcttctc gcgcaacgcg tcagtgggct gatcattaac tatccgctgg atgaccagga

3721 tgccattgct gtggaagctg cctgcactaa tgttccggcg ttatttcttg atgtctctga

3781 ccagacaccc atcaacagta ttattttctc ccatgaagac ggtacgcgac tgggcgtgga

3841 gcatctggtc gcattgggtc accagcaaat cgcgctgtta gcgggcccat taagttctgt

3901 ctcggcgcgt ctgcgtctgg ctggctggca taaatatctc actcgcaatc aaattcagcc

3961 gatagcggaa cgggaaggcg actggagtgc catgtccggt tttcaacaaa ccatgcaaat

4021 gctgaatgag ggcatcgttc ccactgcgat gctggttgcc aacgatcaga tggcgctggg

4081 cgcaatgcgc gccattaccg agtccgggct gcgcgttggt gcggatatct cggtagtggg

4141 atacgacgat accgaagaca gctcatgtta tatcccgccg ttaaccacca tcaaacagga

4201 ttttcgcctg ctggggcaaa ccagcgtgga ccgcttgctg caactctctc agggccaggc

4261 ggtgaagggc aatcagctgt tgcccgtctc actggtgaaa agaaaaacca ccctggcgcc

4321 caatacgcaa accgcctctc cccgcgcgtt ggccgattca ttaatgcagc tggcacgaca

4381 ggtttcccga ctggaaagcg ggcagtgagc gcaacgcaat taatgtaagt tagcgcgaat

4441 tgatctg

//

**Figure E in S1 file. *Lactococcus lactis* reverse orientation target (LLA-r) plasmid.** LLA crRNA target sequence is in the reverse orientation (yellow, LLA-r target), relative to the trc promoter (blue). The target sequence is inserted between the IPTG-inducible lac operator (teal) and rrnB T1/T2 terminators (red) of the pTrcHis-TOPO plasmid, which carries the colE1 origin of replication (colE1 ori, olive color) and ampicillin resistance marker (purple). Green arrows represent AmpR and lacIq transcriptional promoters. Plasmid map illustration made using the SnapGene program. Plasmid sequence information is in GenBank format, using ApE program.


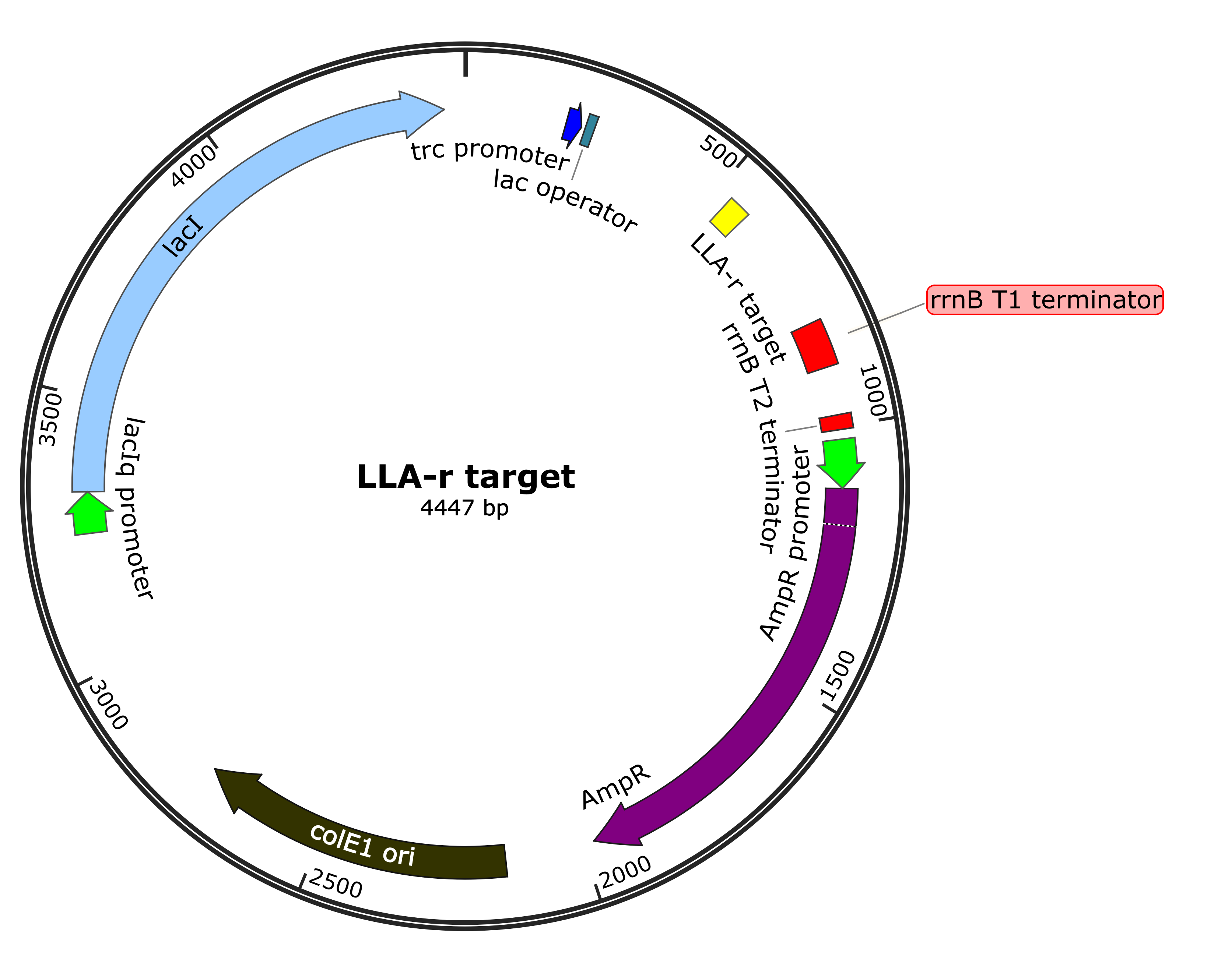


LOCUS LLA_r_target 4447 bp ds-DNA circular 19-FEB-2017

DEFINITION synthetic circular DNA

ACCESSION .

VERSION .

KEYWORDS LLA-r target

SOURCE synthetic DNA construct

ORGANISM synthetic DNA construct

REFERENCE 1 (bases 1 to 4447)

AUTHORS Chris Cooper

TITLE Direct Submission

JOURNAL Exported Sunday, Feb 19, 2017 from SnapGene 3.3.3

http://www.snapgene.com

COMMENT

COMMENT ApEinfo:methylated:1

FEATURES Location/Qualifiers

promoter 193..222

/note="trc promoter"

/note="strong E. coli promoter; hybrid between the trp and

lac UV5 promoters"

/label=trc promoter

/ApEinfo_fwdcolor=#0000ff

/ApEinfo_revcolor=#0000ff

/ApEinfo_graphicformat=arrow_data {{0 1 2 0 0 -1} {} 0}

width 5 offset 0

protein_bind 230..246

/bound_moiety="lac repressor encoded by lacI"

/note="lac operator"

/note="The lac repressor binds to the lac operator to

inhibit transcription in E. coli. This inhibition can be

relieved by adding lactose or

isopropyl-beta-D-thiogalactopyranoside (IPTG)."

/label=lac operator

/ApEinfo_fwdcolor=#008080

/ApEinfo_revcolor=#008080

/ApEinfo_graphicformat=arrow_data {{0 1 2 0 0 -1} {} 0}

width 5 offset 0

misc_feature 529..571

/note="LLA-r target"

/label=LLA-r target

/ApEinfo_fwdcolor=#ffff00

/ApEinfo_revcolor=#ffff00

/ApEinfo_graphicformat=arrow_data {{0 1 2 0 0 -1} {} 0}

width 5 offset 0

terminator 801..887

/gene="Escherichia coli rrnB"

/note="rrnB T1 terminator"

/note="transcription terminator T1 from the E. coli rrnB

gene"

/label=rrnB T1 terminator

/ApEinfo_fwdcolor=#ff0000

/ApEinfo_revcolor=#ff0000

/ApEinfo_graphicformat=arrow_data {{0 1 2 0 0 -1} {} 0}

width 5 offset 0

terminator 979..1006

/note="rrnB T2 terminator"

/note="transcription terminator T2 from the E. coli rrnB

gene"

/label=rrnB T2 terminator

/ApEinfo_fwdcolor=#ff0000

/ApEinfo_revcolor=#ff0000

/ApEinfo_graphicformat=arrow_data {{0 1 2 0 0 -1} {} 0}

width 5 offset 0

promoter 1025..1116

/gene="bla"

/note="AmpR promoter"

/label=AmpR promoter

/ApEinfo_fwdcolor=#00ff00

/ApEinfo_revcolor=#00ff00

/ApEinfo_graphicformat=arrow_data {{0 1 2 0 0 -1} {} 0}

width 5 offset 0

CDS 1117..1977

/codon_start=1

/gene="bla"

/product="beta-lactamase"

/note="AmpR"

/note="confers resistance to ampicillin, carbenicillin,

and related antibiotics"

/translation="MSIQHFRVALIPFFAAFCLPVFAHPETLVKVKDAEDQLGARVGYIELDLNSGKILESFRPEERFPMMSTFKVLLCGAVLSRVDAGQEQLGRRIHYSQNDLVEYSPVTEKHLTDGMTVRELCSAAITMSDNTAANLLLTTIGGPKELTAFLHNMGDHVTRLDRWEPELNEAIPNDERDTTMPVAMATTLRKLLTGELLTLASRQQLIDWMEADKVAGPLLRSALPAGWFIADKSGAGERGSRGIIAALGPDGKPSRIVVIYTTGSQATMDERNRQIAEIGASLIKHW"

/label=AmpR

/ApEinfo_fwdcolor=#800080

/ApEinfo_revcolor=#800080

/ApEinfo_graphicformat=arrow_data {{0 1 2 0 0 -1} {} 0}

width 5 offset 0

rep_origin 2148..2736

/direction=RIGHT

/note="colE1 ori"

/note="high-copy-number ColE1/pMB1/pBR322/pUC origin of

replication"

/label=colE1 ori

/ApEinfo_fwdcolor=#808000

/ApEinfo_revcolor=#808000

/ApEinfo_graphicformat=arrow_data {{0 1 2 0 0 -1} {} 0}

width 5 offset 0

promoter 3248..3325

/gene="lacI (mutant)"

/note="lacIq promoter"

/note="In the lacIq allele, a single base change in the

promoter boosts expression of the lacI gene about

10-fold."

/label=lacIq promoter

/ApEinfo_fwdcolor=#00ff00

/ApEinfo_revcolor=#00ff00

/ApEinfo_graphicformat=arrow_data {{0 1 2 0 0 -1} {} 0}

width 5 offset 0

CDS 3326..4408

/codon_start=1

/gene="lacI"

/product="lac repressor"

/note="lacI"

/note="The lac repressor binds to the lac operator to

inhibit transcription in E. coli. This inhibition can be

relieved by adding lactose or

isopropyl-beta-D-thiogalactopyranoside (IPTG)."

/translation="MKPVTLYDVAEYAGVSYQTVSRVVNQASHVSAKTREKVEAAMAELNYIPNRVAQQLAGKQSLLIGVATSSLALHAPSQIVAAIKSRADQLGASVVVSMVERSGVEACKAAVHNLLAQRVSGLIINYPLDDQDAIAVEAACTNVPALFLDVSDQTPINSIIFSHEDGTRLGVEHLVALGHQQIALLAGPLSSVSARLRLAGWHKYLTRNQIQPIAEREGDWSAMSGFQQTMQMLNEGIVPTAMLVANDQMALGAMRAITESGLRVGADISVVGYDDTEDSSCYIPPLTTIKQDFRLLGQTSVDRLLQLSQGQAVKGNQLLPVSLVKRKTTLAPNTQTASPRALADSLMQLARQVSRLESGQ"

/label=lacI

/ApEinfo_fwdcolor=#66ccff

/ApEinfo_revcolor=#66ccff

/ApEinfo_graphicformat=arrow_data {{0 1 2 0 0 -1} {} 0}

width 5 offset 0

ORIGIN

1 gtttgacagc ttatcatcga ctgcacggtg caccaatgct tctggcgtca ggcagccatc

61 ggaagctgtg gtatggctgt gcaggtcgta aatcactgca taattcgtgt cgctcaaggc

121 gcactcccgt tctggataat gttttttgcg ccgacatcat aacggttctg gcaaatattc

181 tgaaatgagc tgttgacaat taatcatccg gctcgtataa tgtgtggaat tgtgagcgga

241 taacaatttc acacaggaaa cagcgccgct gagaaaaagc gaagcggcac tgctctttaa

301 caatttatca gacaatctgt gtgggcactc gaccggaatt atcgattaac tttattatta

361 aaaattaaag aggtatatat taatgtatcg attaaataag gaggaataaa ccatgggggg

421 ttctcatcat catcatcatc atggtatggc tagcatgact ggtggacagc aaatgggtcg

481 ggatctgtac gacgatgacg ataaggatcc aacccttgga agctatgctg ctcttgatac

541 gttctttgaa ccaagcttca actccctcgg aaagcttgga aaggcgaatt cgaagcttgg

601 ctgttttggc ggatgagaga agattttcag cctgatacag attaaatcag aacgcagaag

661 cggtctgata aaacagaatt tgcctggcgg cagtagcgcg gtggtcccac ctgaccccat

721 gccgaactca gaagtgaaac gccgtagcgc cgatggtagt gtggggtctc cccatgcgag

781 agtagggaac tgccaggcat caaataaaac gaaaggctca gtcgaaagac tgggcctttc

841 gttttatctg ttgtttgtcg gtgaacgctc tcctgagtag gacaaatccg ccgggagcgg

901 atttgaacgt tgcgaagcaa cggcccggag ggtggcgggc aggacgcccg ccataaactg

961 ccaggcatca aattaagcag aaggccatcc tgacggatgg cctttttgcg tttctacaaa

1021 ctcttttgtt tatttttcta aatacattca aatatgtatc cgctcatgag acaataaccc

1081 tgataaatgc ttcaataata ttgaaaaagg aagagtatga gtattcaaca tttccgtgtc

1141 gcccttattc ccttttttgc ggcattttgc cttcctgttt ttgctcaccc agaaacgctg

1201 gtgaaagtaa aagatgctga agatcagttg ggtgcacgag tgggttacat cgaactggat

1261 ctcaacagcg gtaagatcct tgagagtttt cgccccgaag aacgttttcc aatgatgagc

1321 acttttaaag ttctgctatg tggcgcggta ttatcccgtg ttgacgccgg gcaagagcaa

1381 ctcggtcgcc gcatacacta ttctcagaat gacttggttg agtactcacc agtcacagaa

1441 aagcatctta cggatggcat gacagtaaga gaattatgca gtgctgccat aaccatgagt

1501 gataacactg cggccaactt acttctgaca acgatcggag gaccgaagga gctaaccgct

1561 tttttgcaca acatggggga tcatgtaact cgccttgatc gttgggaacc ggagctgaat

1621 gaagccatac caaacgacga gcgtgacacc acgatgcctg tagcaatggc aacaacgttg

1681 cgcaaactat taactggcga actacttact ctagcttccc ggcaacaatt aatagactgg

1741 atggaggcgg ataaagttgc aggaccactt ctgcgctcgg cccttccggc tggctggttt

1801 attgctgata aatctggagc cggtgagcgt gggtctcgcg gtatcattgc agcactgggg

1861 ccagatggta agccctcccg tatcgtagtt atctacacga cggggagtca ggcaactatg

1921 gatgaacgaa atagacagat cgctgagata ggtgcctcac tgattaagca ttggtaactg

1981 tcagaccaag tttactcata tatactttag attgatttaa aacttcattt ttaatttaaa

2041 aggatctagg tgaagatcct ttttgataat ctcatgacca aaatccctta acgtgagttt

2101 tcgttccact gagcgtcaga ccccgtagaa aagatcaaag gatcttcttg agatcctttt

2161 tttctgcgcg taatctgctg cttgcaaaca aaaaaaccac cgctaccagc ggtggtttgt

2221 ttgccggatc aagagctacc aactcttttt ccgaaggtaa ctggcttcag cagagcgcag

2281 ataccaaata ctgtccttct agtgtagccg tagttaggcc accacttcaa gaactctgta

2341 gcaccgccta catacctcgc tctgctaatc ctgttaccag tggctgctgc cagtggcgat

2401 aagtcgtgtc ttaccgggtt ggactcaaga cgatagttac cggataaggc gcagcggtcg

2461 ggctgaacgg ggggttcgtg cacacagccc agcttggagc gaacgaccta caccgaactg

2521 agatacctac agcgtgagct atgagaaagc gccacgcttc ccgaagggag aaaggcggac

2581 aggtatccgg taagcggcag ggtcggaaca ggagagcgca cgagggagct tccaggggga

2641 aacgcctggt atctttatag tcctgtcggg tttcgccacc tctgacttga gcgtcgattt

2701 ttgtgatgct cgtcaggggg gcggagccta tggaaaaacg ccagcaacgc ggccttttta

2761 cggttcctgg ccttttgctg gccttttgct cacatgttct ttcctgcgtt atcccctgat

2821 tctgtggata accgtattac cgcctttgag tgagctgata ccgctcgccg cagccgaacg

2881 accgagcgca gcgagtcagt gagcgaggaa gcggaagagc gcctgatgcg gtattttctc

2941 cttacgcatc tgtgcggtat ttcacaccgc atatggtgca ctctcagtac aatctgctct

3001 gatgccgcat agttaagcca gtatacactc cgctatcgct acgtgactgg gtcatggctg

3061 cgccccgaca cccgccaaca cccgctgacg cgccctgacg ggcttgtctg ctcccggcat

3121 ccgcttacag acaagctgtg accgtctccg ggagctgcat gtgtcagagg ttttcaccgt

3181 catcaccgaa acgcgcgagg cagcagatca attcgcgcgc gaaggcgaag cggcatgcat

3241 ttacgttgac accatcgaat ggtgcaaaac ctttcgcggt atggcatgat agcgcccgga

3301 agagagtcaa ttcagggtgg tgaatgtgaa accagtaacg ttatacgatg tcgcagagta

3361 tgccggtgtc tcttatcaga ccgtttcccg cgtggtgaac caggccagcc acgtttctgc

3421 gaaaacgcgg gaaaaagtgg aagcggcgat ggcggagctg aattacattc ccaaccgcgt

3481 ggcacaacaa ctggcgggca aacagtcgtt gctgattggc gttgccacct ccagtctggc

3541 cctgcacgcg ccgtcgcaaa ttgtcgcggc gattaaatct cgcgccgatc aactgggtgc

3601 cagcgtggtg gtgtcgatgg tagaacgaag cggcgtcgaa gcctgtaaag cggcggtgca

3661 caatcttctc gcgcaacgcg tcagtgggct gatcattaac tatccgctgg atgaccagga

3721 tgccattgct gtggaagctg cctgcactaa tgttccggcg ttatttcttg atgtctctga

3781 ccagacaccc atcaacagta ttattttctc ccatgaagac ggtacgcgac tgggcgtgga

3841 gcatctggtc gcattgggtc accagcaaat cgcgctgtta gcgggcccat taagttctgt

3901 ctcggcgcgt ctgcgtctgg ctggctggca taaatatctc actcgcaatc aaattcagcc

3961 gatagcggaa cgggaaggcg actggagtgc catgtccggt tttcaacaaa ccatgcaaat

4021 gctgaatgag ggcatcgttc ccactgcgat gctggttgcc aacgatcaga tggcgctggg

4081 cgcaatgcgc gccattaccg agtccgggct gcgcgttggt gcggatatct cggtagtggg

4141 atacgacgat accgaagaca gctcatgtta tatcccgccg ttaaccacca tcaaacagga

4201 ttttcgcctg ctggggcaaa ccagcgtgga ccgcttgctg caactctctc agggccaggc

4261 ggtgaagggc aatcagctgt tgcccgtctc actggtgaaa agaaaaacca ccctggcgcc

4321 caatacgcaa accgcctctc cccgcgcgtt ggccgattca ttaatgcagc tggcacgaca

4381 ggtttcccga ctggaaagcg ggcagtgagc gcaacgcaat taatgtaagt tagcgcgaat

4441 tgatctg

//

**Figure F in S1 file. *Lactococcus lactis* forward orientation target with flanking terminators and no promoter (t-LLA-f-t) plasmid.** LLA crRNA target sequence is in the forward orientation (yellow, LLA-f target) inserted between the T1 and T2 terminators (red), upstream of the rrnB T1/T2 terminators (red) of the pTrcHis-TOPO plasmid, which carries the colE1 origin of replication (colE1 ori, olive color) and ampicillin resistance marker (purple). Green arrows represent AmpR and lacIq transcriptional promoters. Plasmid map illustration made using the SnapGene program. Plasmid sequence information is in GenBank format, using ApE program.


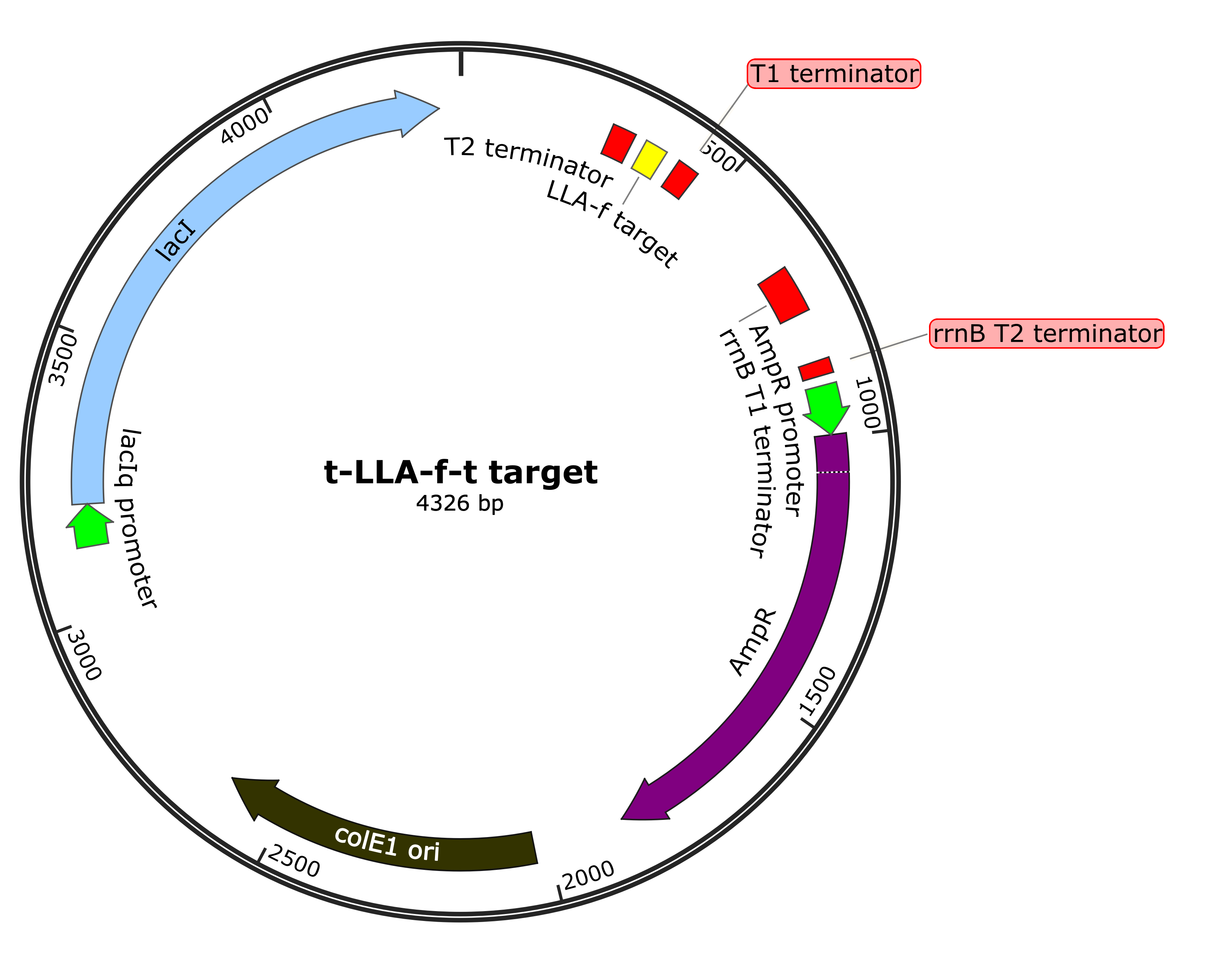


LOCUS t_LLA_f_t_target 4326 bp ds-DNA circular 19-FEB-2017

DEFINITION synthetic circular DNA

ACCESSION .

VERSION .

KEYWORDS t-LLA-f-t target

SOURCE synthetic DNA construct

ORGANISM synthetic DNA construct

REFERENCE 1 (bases 1 to 4326)

AUTHORS .

TITLE Direct Submission

JOURNAL Exported Sunday, Feb 19, 2017 from SnapGene 3.3.3

http://www.snapgene.com

COMMENT

COMMENT ApEinfo:methylated:1

FEATURES Location/Qualifiers

terminator 280..323

/note="T2 terminator"

/label=T2 terminator

/ApEinfo_fwdcolor=#ff0000

/ApEinfo_revcolor=#ff0000

/ApEinfo_graphicformat=arrow_data {{0 1 2 0 0 -1} {} 0}

width 5 offset 0

misc_feature 345..386

/note="LLA-f target"

/label=LLA-f target

/ApEinfo_fwdcolor=#ffff00

/ApEinfo_revcolor=#ffff00

/ApEinfo_graphicformat=arrow_data {{0 1 2 0 0 -1} {} 0}

width 5 offset 0

terminator 413..453

/note="T1 terminator"

/label=T1 terminator

/ApEinfo_fwdcolor=#ff0000

/ApEinfo_revcolor=#ff0000

/ApEinfo_graphicformat=arrow_data {{0 1 2 0 0 -1} {} 0}

width 5 offset 0

terminator 680..766

/gene="Escherichia coli rrnB"

/note="rrnB T1 terminator"

/note="transcription terminator T1 from the E. coli rrnB

gene"

/label=rrnB T1 terminator

/ApEinfo_fwdcolor=#ff0000

/ApEinfo_revcolor=#ff0000

/ApEinfo_graphicformat=arrow_data {{0 1 2 0 0 -1} {} 0}

width 5 offset 0

terminator 858..885

/note="rrnB T2 terminator"

/note="transcription terminator T2 from the E. coli rrnB

gene"

/label=rrnB T2 terminator

/ApEinfo_fwdcolor=#ff0000

/ApEinfo_revcolor=#ff0000

/ApEinfo_graphicformat=arrow_data {{0 1 2 0 0 -1} {} 0}

width 5 offset 0

promoter 904..995

/gene="bla"

/note="AmpR promoter"

/label=AmpR promoter

/ApEinfo_fwdcolor=#00ff00

/ApEinfo_revcolor=#00ff00

/ApEinfo_graphicformat=arrow_data {{0 1 2 0 0 -1} {} 0}

width 5 offset 0

CDS 996..1856

/codon_start=1

/gene="bla"

/product="beta-lactamase"

/note="AmpR"

/note="confers resistance to ampicillin, carbenicillin,

and related antibiotics"

/translation="MSIQHFRVALIPFFAAFCLPVFAHPETLVKVKDAEDQLGARVGYIELDLNSGKILESFRPEERFPMMSTFKVLLCGAVLSRVDAGQEQLGRRIHYSQNDLVEYSPVTEKHLTDGMTVRELCSAAITMSDNTAANLLLTTIGGPKELTAFLHNMGDHVTRLDRWEPELNEAIPNDERDTTMPVAMATTLRKLLTGELLTLASRQQLIDWMEADKVAGPLLRSALPAGWFIADKSGAGERGSRGIIAALGPDGKPSRIVVIYTTGSQATMDERNRQIAEIGASLIKHW"

/label=AmpR

/ApEinfo_fwdcolor=#800080

/ApEinfo_revcolor=#800080

/ApEinfo_graphicformat=arrow_data {{0 1 2 0 0 -1} {} 0}

width 5 offset 0

rep_origin 2027..2615

/direction=RIGHT

/note="colE1 ori"

/note="high-copy-number ColE1/pMB1/pBR322/pUC origin of

replication"

/label=colE1 ori

/ApEinfo_fwdcolor=#808000

/ApEinfo_revcolor=#808000

/ApEinfo_graphicformat=arrow_data {{0 1 2 0 0 -1} {} 0}

width 5 offset 0

promoter 3127..3204

/gene="lacI (mutant)"

/note="lacIq promoter"

/note="In the lacIq allele, a single base change in the

promoter boosts expression of the lacI gene about

10-fold."

/label=lacIq promoter

/ApEinfo_fwdcolor=#00ff00

/ApEinfo_revcolor=#00ff00

/ApEinfo_graphicformat=arrow_data {{0 1 2 0 0 -1} {} 0}

width 5 offset 0

CDS 3205..4287

/codon_start=1

/gene="lacI"

/product="lac repressor"

/note="lacI"

/note="The lac repressor binds to the lac operator to

inhibit transcription in E. coli. This inhibition can be

relieved by adding lactose or

isopropyl-beta-D-thiogalactopyranoside (IPTG)."

/translation="MKPVTLYDVAEYAGVSYQTVSRVVNQASHVSAKTREKVEAAMAELNYIPNRVAQQLAGKQSLLIGVATSSLALHAPSQIVAAIKSRADQLGASVVVSMVERSGVEACKAAVHNLLAQRVSGLIINYPLDDQDAIAVEAACTNVPALFLDVSDQTPINSIIFSHEDGTRLGVEHLVALGHQQIALLAGPLSSVSARLRLAGWHKYLTRNQIQPIAEREGDWSAMSGFQQTMQMLNEGIVPTAMLVANDQMALGAMRAITESGLRVGADISVVGYDDTEDSSCYIPPLTTIKQDFRLLGQTSVDRLLQLSQGQAVKGNQLLPVSLVKRKTTLAPNTQTASPRALADSLMQLARQVSRLESGQ"

/label=lacI

/ApEinfo_fwdcolor=#66ccff

/ApEinfo_revcolor=#66ccff

/ApEinfo_graphicformat=arrow_data {{0 1 2 0 0 -1} {} 0}

width 5 offset 0

ORIGIN

1 gtttgacagc ttatcatcga ctgcacggtg caccaatgct tctggcgtca ggcagccatc

61 ggaagctgtg gtatggctgt gcaggtcgta aatcactgca taattcgtgt cgctcaaggc

121 gcactcccgt tctggataat gttttttgcg ccgacatcat aacggttctg gcccatgggg

181 ggttctcatc atcatcatca tcatggtatg gctagcatga ctggtggaca gcaaatgggt

241 cgggatctgt acgacgatga cgataaggat ccaacccttc aaacaaaaaa accaccgcta

301 ccagcgtggt ttgtttgccg gatgaggtaa ctggcttctg cagtgctctt gatacgttct

361 ttgaaccaag cttcaactcc ctcggaggta ccttggaacc tcttacgtgc cgatcaagtc

421 aaaagcctcc ggtcggaggc ttttgacttt ctgcatatga aggcgaattc gaagcttggc

481 tgttttggcg gatgagagaa gattttcagc ctgatacaga ttaaatcaga acgcagaagc

541 ggtctgataa aacagaattt gcctggcggc agtagcgcgg tggtcccacc tgaccccatg

601 ccgaactcag aagtgaaacg ccgtagcgcc gatggtagtg tggggtctcc ccatgcgaga

661 gtagggaact gccaggcatc aaataaaacg aaaggctcag tcgaaagact gggcctttcg

721 ttttatctgt tgtttgtcgg tgaacgctct cctgagtagg acaaatccgc cgggagcgga

781 tttgaacgtt gcgaagcaac ggcccggagg gtggcgggca ggacgcccgc cataaactgc

841 caggcatcaa attaagcaga aggccatcct gacggatggc ctttttgcgt ttctacaaac

901 tcttttgttt atttttctaa atacattcaa atatgtatcc gctcatgaga caataaccct

961 gataaatgct tcaataatat tgaaaaagga agagtatgag tattcaacat ttccgtgtcg

1021 cccttattcc cttttttgcg gcattttgcc ttcctgtttt tgctcaccca gaaacgctgg

1081 tgaaagtaaa agatgctgaa gatcagttgg gtgcacgagt gggttacatc gaactggatc

1141 tcaacagcgg taagatcctt gagagttttc gccccgaaga acgttttcca atgatgagca

1201 cttttaaagt tctgctatgt ggcgcggtat tatcccgtgt tgacgccggg caagagcaac

1261 tcggtcgccg catacactat tctcagaatg acttggttga gtactcacca gtcacagaaa

1321 agcatcttac ggatggcatg acagtaagag aattatgcag tgctgccata accatgagtg

1381 ataacactgc ggccaactta cttctgacaa cgatcggagg accgaaggag ctaaccgctt

1441 ttttgcacaa catgggggat catgtaactc gccttgatcg ttgggaaccg gagctgaatg

1501 aagccatacc aaacgacgag cgtgacacca cgatgcctgt agcaatggca acaacgttgc

1561 gcaaactatt aactggcgaa ctacttactc tagcttcccg gcaacaatta atagactgga

1621 tggaggcgga taaagttgca ggaccacttc tgcgctcggc ccttccggct ggctggttta

1681 ttgctgataa atctggagcc ggtgagcgtg ggtctcgcgg tatcattgca gcactggggc

1741 cagatggtaa gccctcccgt atcgtagtta tctacacgac ggggagtcag gcaactatgg

1801 atgaacgaaa tagacagatc gctgagatag gtgcctcact gattaagcat tggtaactgt

1861 cagaccaagt ttactcatat atactttaga ttgatttaaa acttcatttt taatttaaaa

1921 ggatctaggt gaagatcctt tttgataatc tcatgaccaa aatcccttaa cgtgagtttt

1981 cgttccactg agcgtcagac cccgtagaaa agatcaaagg atcttcttga gatccttttt

2041 ttctgcgcgt aatctgctgc ttgcaaacaa aaaaaccacc gctaccagcg gtggtttgtt

2101 tgccggatca agagctacca actctttttc cgaaggtaac tggcttcagc agagcgcaga

2161 taccaaatac tgtccttcta gtgtagccgt agttaggcca ccacttcaag aactctgtag

2221 caccgcctac atacctcgct ctgctaatcc tgttaccagt ggctgctgcc agtggcgata

2281 agtcgtgtct taccgggttg gactcaagac gatagttacc ggataaggcg cagcggtcgg

2341 gctgaacggg gggttcgtgc acacagccca gcttggagcg aacgacctac accgaactga

2401 gatacctaca gcgtgagcta tgagaaagcg ccacgcttcc cgaagggaga aaggcggaca

2461 ggtatccggt aagcggcagg gtcggaacag gagagcgcac gagggagctt ccagggggaa

2521 acgcctggta tctttatagt cctgtcgggt ttcgccacct ctgacttgag cgtcgatttt

2581 tgtgatgctc gtcagggggg cggagcctat ggaaaaacgc cagcaacgcg gcctttttac

2641 ggttcctggc cttttgctgg ccttttgctc acatgttctt tcctgcgtta tcccctgatt

2701 ctgtggataa ccgtattacc gcctttgagt gagctgatac cgctcgccgc agccgaacga

2761 ccgagcgcag cgagtcagtg agcgaggaag cggaagagcg cctgatgcgg tattttctcc

2821 ttacgcatct gtgcggtatt tcacaccgca tatggtgcac tctcagtaca atctgctctg

2881 atgccgcata gttaagccag tatacactcc gctatcgcta cgtgactggg tcatggctgc

2941 gccccgacac ccgccaacac ccgctgacgc gccctgacgg gcttgtctgc tcccggcatc

3001 cgcttacaga caagctgtga ccgtctccgg gagctgcatg tgtcagaggt tttcaccgtc

3061 atcaccgaaa cgcgcgaggc agcagatcaa ttcgcgcgcg aaggcgaagc ggcatgcatt

3121 tacgttgaca ccatcgaatg gtgcaaaacc tttcgcggta tggcatgata gcgcccggaa

3181 gagagtcaat tcagggtggt gaatgtgaaa ccagtaacgt tatacgatgt cgcagagtat

3241 gccggtgtct cttatcagac cgtttcccgc gtggtgaacc aggccagcca cgtttctgcg

3301 aaaacgcggg aaaaagtgga agcggcgatg gcggagctga attacattcc caaccgcgtg

3361 gcacaacaac tggcgggcaa acagtcgttg ctgattggcg ttgccacctc cagtctggcc

3421 ctgcacgcgc cgtcgcaaat tgtcgcggcg attaaatctc gcgccgatca actgggtgcc

3481 agcgtggtgg tgtcgatggt agaacgaagc ggcgtcgaag cctgtaaagc ggcggtgcac

3541 aatcttctcg cgcaacgcgt cagtgggctg atcattaact atccgctgga tgaccaggat

3601 gccattgctg tggaagctgc ctgcactaat gttccggcgt tatttcttga tgtctctgac

3661 cagacaccca tcaacagtat tattttctcc catgaagacg gtacgcgact gggcgtggag

3721 catctggtcg cattgggtca ccagcaaatc gcgctgttag cgggcccatt aagttctgtc

3781 tcggcgcgtc tgcgtctggc tggctggcat aaatatctca ctcgcaatca aattcagccg

3841 atagcggaac gggaaggcga ctggagtgcc atgtccggtt ttcaacaaac catgcaaatg

3901 ctgaatgagg gcatcgttcc cactgcgatg ctggttgcca acgatcagat ggcgctgggc

3961 gcaatgcgcg ccattaccga gtccgggctg cgcgttggtg cggatatctc ggtagtggga

4021 tacgacgata ccgaagacag ctcatgttat atcccgccgt taaccaccat caaacaggat

4081 tttcgcctgc tggggcaaac cagcgtggac cgcttgctgc aactctctca gggccaggcg

4141 gtgaagggca atcagctgtt gcccgtctca ctggtgaaaa gaaaaaccac cctggcgccc

4201 aatacgcaaa ccgcctctcc ccgcgcgttg gccgattcat taatgcagct ggcacgacag

4261 gtttcccgac tggaaagcgg gcagtgagcg caacgcaatt aatgtaagtt agcgcgaatt

4321 gatctg

//

**Figure G in S1 file. Heterologous forward orientation target (HET-f) plasmid.** A crRNA target sequence with no homology to LLA crRNA is in the forward orientation (yellow, HET-f target), relative to the trc promoter (blue). The target sequence is inserted between the IPTG-inducible lac operator (teal) and rrnB T1/T2 terminators (red) of the pTrcHis-TOPO plasmid, which carries the colE1 origin of replication (colE1 ori, olive color) and ampicillin resistance marker (purple). Green arrows represent AmpR and lacIq transcriptional promoters. Plasmid map illustration made using the SnapGene program. Plasmid sequence information is in GenBank format, using ApE program.


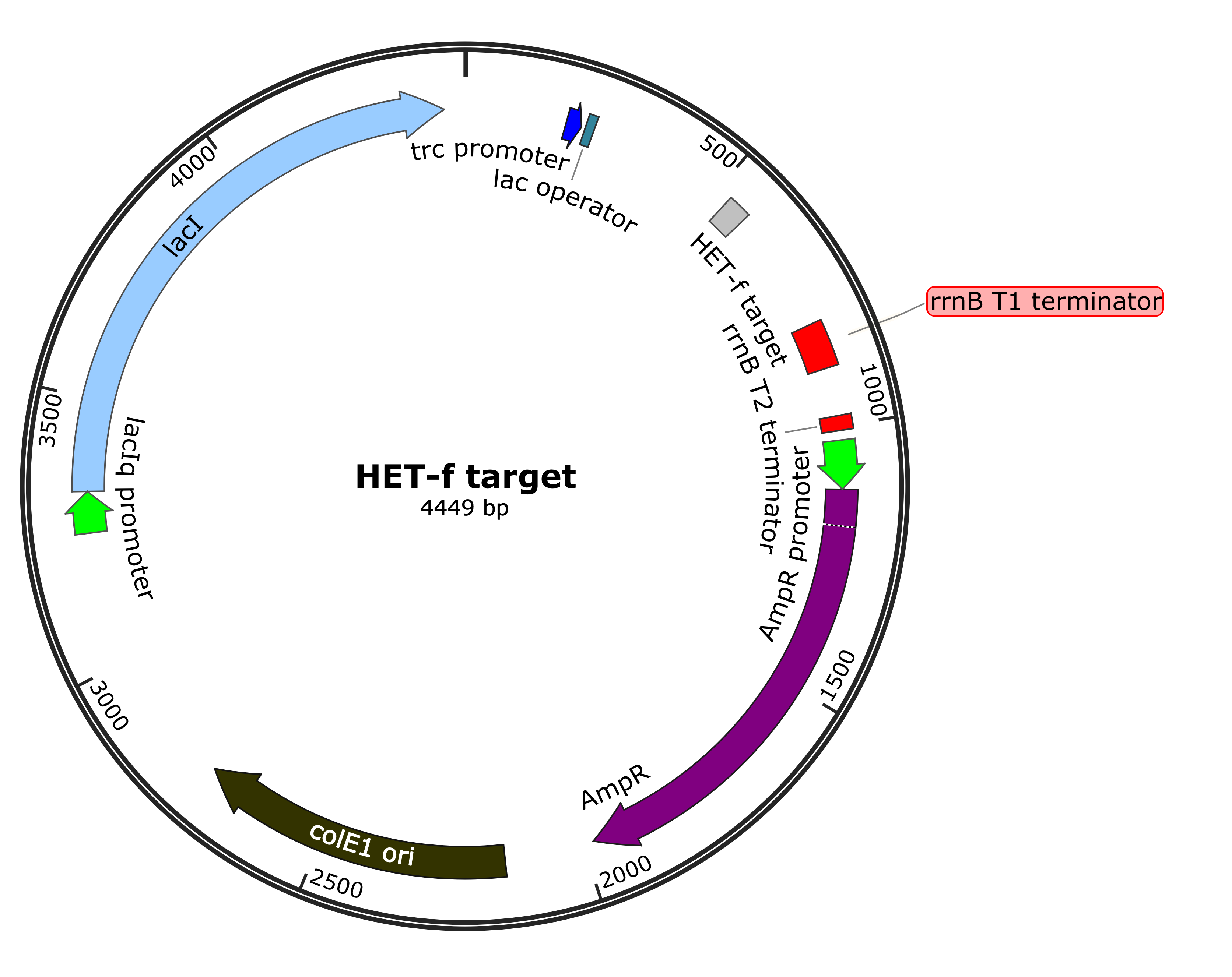


LOCUS HET_f_target 4449 bp ds-DNA circular 19-FEB-2017

DEFINITION synthetic circular DNA

ACCESSION .

VERSION .

KEYWORDS HET-f target

SOURCE synthetic DNA construct

ORGANISM synthetic DNA construct

REFERENCE 1 (bases 1 to 4449)

AUTHORS .

TITLE Direct Submission

JOURNAL Exported Sunday, Feb 19, 2017 from SnapGene 3.3.3

http://www.snapgene.com

COMMENT

COMMENT ApEinfo:methylated:1

FEATURES Location/Qualifiers

promoter 193..222

/note="trc promoter"

/note="strong E. coli promoter; hybrid between the trp and

lac UV5 promoters"

/label=trc promoter

/ApEinfo_fwdcolor=#0000ff

/ApEinfo_revcolor=#0000ff

/ApEinfo_graphicformat=arrow_data {{0 1 2 0 0 -1} {} 0}

width 5 offset 0

protein_bind 230..246

/bound_moiety="lac repressor encoded by lacI"

/note="lac operator"

/note="The lac repressor binds to the lac operator to

inhibit transcription in E. coli. This inhibition can be

relieved by adding lactose or

isopropyl-beta-D-thiogalactopyranoside (IPTG)."

/label=lac operator

/ApEinfo_fwdcolor=#008080

/ApEinfo_revcolor=#008080

/ApEinfo_graphicformat=arrow_data {{0 1 2 0 0 -1} {} 0}

width 5 offset 0

misc_feature 528..572

/note="HET-f target"

/label=HET-f target

/ApEinfo_fwdcolor=#b3b3b3

/ApEinfo_revcolor=#b3b3b3

/ApEinfo_graphicformat=arrow_data {{0 1 2 0 0 -1} {} 0}

width 5 offset 0

terminator 803..889

/gene="Escherichia coli rrnB"

/note="rrnB T1 terminator"

/note="transcription terminator T1 from the E. coli rrnB

gene"

/label=rrnB T1 terminator

/ApEinfo_fwdcolor=#ff0000

/ApEinfo_revcolor=#ff0000

/ApEinfo_graphicformat=arrow_data {{0 1 2 0 0 -1} {} 0}

width 5 offset 0

terminator 981..1008

/note="rrnB T2 terminator"

/note="transcription terminator T2 from the E. coli rrnB

gene"

/label=rrnB T2 terminator

/ApEinfo_fwdcolor=#ff0000

/ApEinfo_revcolor=#ff0000

/ApEinfo_graphicformat=arrow_data {{0 1 2 0 0 -1} {} 0}

width 5 offset 0

promoter 1027..1118

/gene="bla"

/note="AmpR promoter"

/label=AmpR promoter

/ApEinfo_fwdcolor=#00ff00

/ApEinfo_revcolor=#00ff00

/ApEinfo_graphicformat=arrow_data {{0 1 2 0 0 -1} {} 0}

width 5 offset 0

CDS 1119..1979

/codon_start=1

/gene="bla"

/product="beta-lactamase"

/note="AmpR"

/note="confers resistance to ampicillin, carbenicillin,

and related antibiotics"

/translation="MSIQHFRVALIPFFAAFCLPVFAHPETLVKVKDAEDQLGARVGYIELDLNSGKILESFRPEERFPMMSTFKVLLCGAVLSRVDAGQEQLGRRIHYSQNDLVEYSPVTEKHLTDGMTVRELCSAAITMSDNTAANLLLTTIGGPKELTAFLHNMGDHVTRLDRWEPELNEAIPNDERDTTMPVAMATTLRKLLTGELLTLASRQQLIDWMEADKVAGPLLRSALPAGWFIADKSGAGERGSRGIIAALGPDGKPSRIVVIYTTGSQATMDERNRQIAEIGASLIKHW"

/label=AmpR

/ApEinfo_fwdcolor=#800080

/ApEinfo_revcolor=#800080

/ApEinfo_graphicformat=arrow_data {{0 1 2 0 0 -1} {} 0}

width 5 offset 0

rep_origin 2150..2738

/direction=RIGHT

/note="colE1 ori"

/note="high-copy-number ColE1/pMB1/pBR322/pUC origin of

replication"

/label=colE1 ori

/ApEinfo_fwdcolor=#808000

/ApEinfo_revcolor=#808000

/ApEinfo_graphicformat=arrow_data {{0 1 2 0 0 -1} {} 0}

width 5 offset 0

promoter 3250..3327

/gene="lacI (mutant)"

/note="lacIq promoter"

/note="In the lacIq allele, a single base change in the

promoter boosts expression of the lacI gene about

10-fold."

/label=lacIq promoter

/ApEinfo_fwdcolor=#00ff00

/ApEinfo_revcolor=#00ff00

/ApEinfo_graphicformat=arrow_data {{0 1 2 0 0 -1} {} 0}

width 5 offset 0

CDS 3328..4410

/codon_start=1

/gene="lacI"

/product="lac repressor"

/note="lacI"

/note="The lac repressor binds to the lac operator to

inhibit transcription in E. coli. This inhibition can be

relieved by adding lactose or

isopropyl-beta-D-thiogalactopyranoside (IPTG)."

/translation="MKPVTLYDVAEYAGVSYQTVSRVVNQASHVSAKTREKVEAAMAELNYIPNRVAQQLAGKQSLLIGVATSSLALHAPSQIVAAIKSRADQLGASVVVSMVERSGVEACKAAVHNLLAQRVSGLIINYPLDDQDAIAVEAACTNVPALFLDVSDQTPINSIIFSHEDGTRLGVEHLVALGHQQIALLAGPLSSVSARLRLAGWHKYLTRNQIQPIAEREGDWSAMSGFQQTMQMLNEGIVPTAMLVANDQMALGAMRAITESGLRVGADISVVGYDDTEDSSCYIPPLTTIKQDFRLLGQTSVDRLLQLSQGQAVKGNQLLPVSLVKRKTTLAPNTQTASPRALADSLMQLARQVSRLESGQ"

/label=lacI

/ApEinfo_fwdcolor=#66ccff

/ApEinfo_revcolor=#66ccff

/ApEinfo_graphicformat=arrow_data {{0 1 2 0 0 -1} {} 0}

width 5 offset 0

ORIGIN

1 gtttgacagc ttatcatcga ctgcacggtg caccaatgct tctggcgtca ggcagccatc

61 ggaagctgtg gtatggctgt gcaggtcgta aatcactgca taattcgtgt cgctcaaggc

121 gcactcccgt tctggataat gttttttgcg ccgacatcat aacggttctg gcaaatattc

181 tgaaatgagc tgttgacaat taatcatccg gctcgtataa tgtgtggaat tgtgagcgga

241 taacaatttc acacaggaaa cagcgccgct gagaaaaagc gaagcggcac tgctctttaa

301 caatttatca gacaatctgt gtgggcactc gaccggaatt atcgattaac tttattatta

361 aaaattaaag aggtatatat taatgtatcg attaaataag gaggaataaa ccatgggggg

421 ttctcatcat catcatcatc atggtatggc tagcatgact ggtggacagc aaatgggtcg

481 ggatctgtac gacgatgacg ataaggatcc aacccttttc caagcttctg aagtgctctc

541 agccgcaagg accgcatact acaacaagag cagcatgctt ccaaggcgaa ttcgaagctt

601 ggctgttttg gcggatgaga gaagattttc agcctgatac agattaaatc agaacgcaga

661 agcggtctga taaaacagaa tttgcctggc ggcagtagcg cggtggtccc acctgacccc

721 atgccgaact cagaagtgaa acgccgtagc gccgatggta gtgtggggtc tccccatgcg

781 agagtaggga actgccaggc atcaaataaa acgaaaggct cagtcgaaag actgggcctt

841 tcgttttatc tgttgtttgt cggtgaacgc tctcctgagt aggacaaatc cgccgggagc

901 ggatttgaac gttgcgaagc aacggcccgg agggtggcgg gcaggacgcc cgccataaac

961 tgccaggcat caaattaagc agaaggccat cctgacggat ggcctttttg cgtttctaca

1021 aactcttttg tttatttttc taaatacatt caaatatgta tccgctcatg agacaataac

1081 cctgataaat gcttcaataa tattgaaaaa ggaagagtat gagtattcaa catttccgtg

1141 tcgcccttat tccctttttt gcggcatttt gccttcctgt ttttgctcac ccagaaacgc

1201 tggtgaaagt aaaagatgct gaagatcagt tgggtgcacg agtgggttac atcgaactgg

1261 atctcaacag cggtaagatc cttgagagtt ttcgccccga agaacgtttt ccaatgatga

1321 gcacttttaa agttctgcta tgtggcgcgg tattatcccg tgttgacgcc gggcaagagc

1381 aactcggtcg ccgcatacac tattctcaga atgacttggt tgagtactca ccagtcacag

1441 aaaagcatct tacggatggc atgacagtaa gagaattatg cagtgctgcc ataaccatga

1501 gtgataacac tgcggccaac ttacttctga caacgatcgg aggaccgaag gagctaaccg

1561 cttttttgca caacatgggg gatcatgtaa ctcgccttga tcgttgggaa ccggagctga

1621 atgaagccat accaaacgac gagcgtgaca ccacgatgcc tgtagcaatg gcaacaacgt

1681 tgcgcaaact attaactggc gaactactta ctctagcttc ccggcaacaa ttaatagact

1741 ggatggaggc ggataaagtt gcaggaccac ttctgcgctc ggcccttccg gctggctggt

1801 ttattgctga taaatctgga gccggtgagc gtgggtctcg cggtatcatt gcagcactgg

1861 ggccagatgg taagccctcc cgtatcgtag ttatctacac gacggggagt caggcaacta

1921 tggatgaacg aaatagacag atcgctgaga taggtgcctc actgattaag cattggtaac

1981 tgtcagacca agtttactca tatatacttt agattgattt aaaacttcat ttttaattta

2041 aaaggatcta ggtgaagatc ctttttgata atctcatgac caaaatccct taacgtgagt

2101 tttcgttcca ctgagcgtca gaccccgtag aaaagatcaa aggatcttct tgagatcctt

2161 tttttctgcg cgtaatctgc tgcttgcaaa caaaaaaacc accgctacca gcggtggttt

2221 gtttgccgga tcaagagcta ccaactcttt ttccgaaggt aactggcttc agcagagcgc

2281 agataccaaa tactgtcctt ctagtgtagc cgtagttagg ccaccacttc aagaactctg

2341 tagcaccgcc tacatacctc gctctgctaa tcctgttacc agtggctgct gccagtggcg

2401 ataagtcgtg tcttaccggg ttggactcaa gacgatagtt accggataag gcgcagcggt

2461 cgggctgaac ggggggttcg tgcacacagc ccagcttgga gcgaacgacc tacaccgaac

2521 tgagatacct acagcgtgag ctatgagaaa gcgccacgct tcccgaaggg agaaaggcgg

2581 acaggtatcc ggtaagcggc agggtcggaa caggagagcg cacgagggag cttccagggg

2641 gaaacgcctg gtatctttat agtcctgtcg ggtttcgcca cctctgactt gagcgtcgat

2701 ttttgtgatg ctcgtcaggg gggcggagcc tatggaaaaa cgccagcaac gcggcctttt

2761 tacggttcct ggccttttgc tggccttttg ctcacatgtt ctttcctgcg ttatcccctg

2821 attctgtgga taaccgtatt accgcctttg agtgagctga taccgctcgc cgcagccgaa

2881 cgaccgagcg cagcgagtca gtgagcgagg aagcggaaga gcgcctgatg cggtattttc

2941 tccttacgca tctgtgcggt atttcacacc gcatatggtg cactctcagt acaatctgct

3001 ctgatgccgc atagttaagc cagtatacac tccgctatcg ctacgtgact gggtcatggc

3061 tgcgccccga cacccgccaa cacccgctga cgcgccctga cgggcttgtc tgctcccggc

3121 atccgcttac agacaagctg tgaccgtctc cgggagctgc atgtgtcaga ggttttcacc

3181 gtcatcaccg aaacgcgcga ggcagcagat caattcgcgc gcgaaggcga agcggcatgc

3241 atttacgttg acaccatcga atggtgcaaa acctttcgcg gtatggcatg atagcgcccg

3301 gaagagagtc aattcagggt ggtgaatgtg aaaccagtaa cgttatacga tgtcgcagag

3361 tatgccggtg tctcttatca gaccgtttcc cgcgtggtga accaggccag ccacgtttct

3421 gcgaaaacgc gggaaaaagt ggaagcggcg atggcggagc tgaattacat tcccaaccgc

3481 gtggcacaac aactggcggg caaacagtcg ttgctgattg gcgttgccac ctccagtctg

3541 gccctgcacg cgccgtcgca aattgtcgcg gcgattaaat ctcgcgccga tcaactgggt

3601 gccagcgtgg tggtgtcgat ggtagaacga agcggcgtcg aagcctgtaa agcggcggtg

3661 cacaatcttc tcgcgcaacg cgtcagtggg ctgatcatta actatccgct ggatgaccag

3721 gatgccattg ctgtggaagc tgcctgcact aatgttccgg cgttatttct tgatgtctct

3781 gaccagacac ccatcaacag tattattttc tcccatgaag acggtacgcg actgggcgtg

3841 gagcatctgg tcgcattggg tcaccagcaa atcgcgctgt tagcgggccc attaagttct

3901 gtctcggcgc gtctgcgtct ggctggctgg cataaatatc tcactcgcaa tcaaattcag

3961 ccgatagcgg aacgggaagg cgactggagt gccatgtccg gttttcaaca aaccatgcaa

4021 atgctgaatg agggcatcgt tcccactgcg atgctggttg ccaacgatca gatggcgctg

4081 ggcgcaatgc gcgccattac cgagtccggg ctgcgcgttg gtgcggatat ctcggtagtg

4141 ggatacgacg ataccgaaga cagctcatgt tatatcccgc cgttaaccac catcaaacag

4201 gattttcgcc tgctggggca aaccagcgtg gaccgcttgc tgcaactctc tcagggccag

4261 gcggtgaagg gcaatcagct gttgcccgtc tcactggtga aaagaaaaac caccctggcg

4321 cccaatacgc aaaccgcctc tccccgcgcg ttggccgatt cattaatgca gctggcacga

4381 caggtttccc gactggaaag cgggcagtga gcgcaacgca attaatgtaa gttagcgcga

4441 attgatctg

//

**Figure H in S1 file. *Staphylococcus epidermidis* forward orientation target (SEP-f) plasmid.** SEP crRNA target sequence is in the forward orientation (yellow, SEP-f target), relative to the trc promoter (blue). The target sequence is inserted between the IPTG-inducible lac operator (teal) and rrnB T1/T2 terminators (red) of the pTrcHis-TOPO plasmid, which carries the colE1 origin of replication (colE1 ori, olive color) and ampicillin resistance marker (purple). Green arrows represent AmpR and lacIq transcriptional promoters. Plasmid map illustration made using the SnapGene program. Plasmid sequence information is in GenBank format, using ApE program.


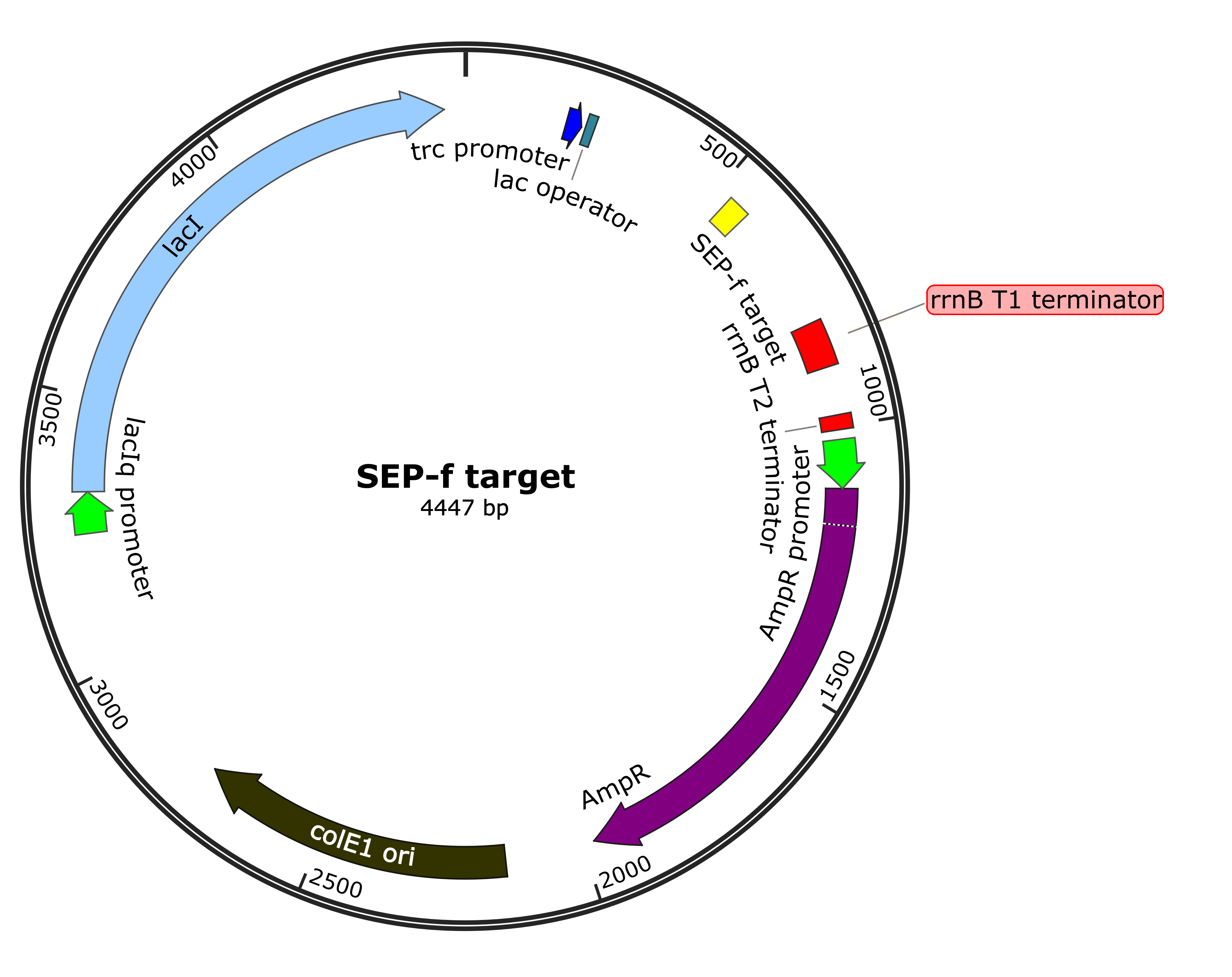


LOCUS SEP_f_target 4447 bp ds-DNA circular 19-FEB-2017

DEFINITION synthetic circular DNA

ACCESSION .

VERSION .

KEYWORDS SEP-f target

SOURCE synthetic DNA construct

ORGANISM synthetic DNA construct

REFERENCE 1 (bases 1 to 4447)

AUTHORS .

TITLE Direct Submission

JOURNAL Exported Sunday, Feb 19, 2017 from SnapGene 3.3.3

http://www.snapgene.com

COMMENT

COMMENT ApEinfo:methylated:1

FEATURES Location/Qualifiers

promoter 193..222

/note="trc promoter"

/note="strong E. coli promoter; hybrid between the trp and

lac UV5 promoters"

/label=trc promoter

/ApEinfo_fwdcolor=#0000ff

/ApEinfo_revcolor=#0000ff

/ApEinfo_graphicformat=arrow_data {{0 1 2 0 0 -1} {} 0}

width 5 offset 0

protein_bind 230..246

/bound_moiety="lac repressor encoded by lacI"

/note="lac operator"

/note="The lac repressor binds to the lac operator to

inhibit transcription in E. coli. This inhibition can be

relieved by adding lactose or

isopropyl-beta-D-thiogalactopyranoside (IPTG)."

/label=lac operator

/ApEinfo_fwdcolor=#008080

/ApEinfo_revcolor=#008080

/ApEinfo_graphicformat=arrow_data {{0 1 2 0 0 -1} {} 0}

width 5 offset 0

misc_feature 528..570

/note="SEP-f target"

/label=SEP-f target

/ApEinfo_fwdcolor=#ffff00

/ApEinfo_revcolor=#ffff00

/ApEinfo_graphicformat=arrow_data {{0 1 2 0 0 -1} {} 0}

width 5 offset 0

terminator 801..887

/gene="Escherichia coli rrnB"

/note="rrnB T1 terminator"

/note="transcription terminator T1 from the E. coli rrnB

gene"

/label=rrnB T1 terminator

/ApEinfo_fwdcolor=#ff0000

/ApEinfo_revcolor=#ff0000

/ApEinfo_graphicformat=arrow_data {{0 1 2 0 0 -1} {} 0}

width 5 offset 0

terminator 979..1006

/note="rrnB T2 terminator"

/note="transcription terminator T2 from the E. coli rrnB

gene"

/label=rrnB T2 terminator

/ApEinfo_fwdcolor=#ff0000

/ApEinfo_revcolor=#ff0000

/ApEinfo_graphicformat=arrow_data {{0 1 2 0 0 -1} {} 0}

width 5 offset 0

promoter 1025..1116

/gene="bla"

/note="AmpR promoter"

/label=AmpR promoter

/ApEinfo_fwdcolor=#00ff00

/ApEinfo_revcolor=#00ff00

/ApEinfo_graphicformat=arrow_data {{0 1 2 0 0 -1} {} 0}

width 5 offset 0

CDS 1117..1977

/codon_start=1

/gene="bla"

/product="beta-lactamase"

/note="AmpR"

/note="confers resistance to ampicillin, carbenicillin,

and related antibiotics"

/translation="MSIQHFRVALIPFFAAFCLPVFAHPETLVKVKDAEDQLGARVGYIELDLNSGKILESFRPEERFPMMSTFKVLLCGAVLSRVDAGQEQLGRRIHYSQNDLVEYSPVTEKHLTDGMTVRELCSAAITMSDNTAANLLLTTIGGPKELTAFLHNMGDHVTRLDRWEPELNEAIPNDERDTTMPVAMATTLRKLLTGELLTLASRQQLIDWMEADKVAGPLLRSALPAGWFIADKSGAGERGSRGIIAALGPDGKPSRIVVIYTTGSQATMDERNRQIAEIGASLIKHW"

/label=AmpR

/ApEinfo_fwdcolor=#800080

/ApEinfo_revcolor=#800080

/ApEinfo_graphicformat=arrow_data {{0 1 2 0 0 -1} {} 0}

width 5 offset 0

rep_origin 2148..2736

/direction=RIGHT

/note="colE1 ori"

/note="high-copy-number ColE1/pMB1/pBR322/pUC origin of

replication"

/label=colE1 ori

/ApEinfo_fwdcolor=#808000

/ApEinfo_revcolor=#808000

/ApEinfo_graphicformat=arrow_data {{0 1 2 0 0 -1} {} 0}

width 5 offset 0

promoter 3248..3325

/gene="lacI (mutant)"

/note="lacIq promoter"

/note="In the lacIq allele, a single base change in the

promoter boosts expression of the lacI gene about

10-fold."

/label=lacIq promoter

/ApEinfo_fwdcolor=#00ff00

/ApEinfo_revcolor=#00ff00

/ApEinfo_graphicformat=arrow_data {{0 1 2 0 0 -1} {} 0}

width 5 offset 0

CDS 3326..4408

/codon_start=1

/gene="lacI"

/product="lac repressor"

/note="lacI"

/note="The lac repressor binds to the lac operator to

inhibit transcription in E. coli. This inhibition can be

relieved by adding lactose or

isopropyl-beta-D-thiogalactopyranoside (IPTG)."

/translation="MKPVTLYDVAEYAGVSYQTVSRVVNQASHVSAKTREKVEAAMAELNYIPNRVAQQLAGKQSLLIGVATSSLALHAPSQIVAAIKSRADQLGASVVVSMVERSGVEACKAAVHNLLAQRVSGLIINYPLDDQDAIAVEAACTNVPALFLDVSDQTPINSIIFSHEDGTRLGVEHLVALGHQQIALLAGPLSSVSARLRLAGWHKYLTRNQIQPIAEREGDWSAMSGFQQTMQMLNEGIVPTAMLVANDQMALGAMRAITESGLRVGADISVVGYDDTEDSSCYIPPLTTIKQDFRLLGQTSVDRLLQLSQGQAVKGNQLLPVSLVKRKTTLAPNTQTASPRALADSLMQLARQVSRLESGQ"

/label=lacI

/ApEinfo_fwdcolor=#66ccff

/ApEinfo_revcolor=#66ccff

/ApEinfo_graphicformat=arrow_data {{0 1 2 0 0 -1} {} 0}

width 5 offset 0

ORIGIN

1 gtttgacagc ttatcatcga ctgcacggtg caccaatgct tctggcgtca ggcagccatc

61 ggaagctgtg gtatggctgt gcaggtcgta aatcactgca taattcgtgt cgctcaaggc

121 gcactcccgt tctggataat gttttttgcg ccgacatcat aacggttctg gcaaatattc

181 tgaaatgagc tgttgacaat taatcatccg gctcgtataa tgtgtggaat tgtgagcgga

241 taacaatttc acacaggaaa cagcgccgct gagaaaaagc gaagcggcac tgctctttaa

301 caatttatca gacaatctgt gtgggcactc gaccggaatt atcgattaac tttattatta

361 aaaattaaag aggtatatat taatgtatcg attaaataag gaggaataaa ccatgggggg

421 ttctcatcat catcatcatc atggtatggc tagcatgact ggtggacagc aaatgggtcg

481 ggatctgtac gacgatgacg ataaggatcc aacccttttc caagcttctt tgtactgatg

541 atttatatac ttcggcatac gtcaagagca gcatgcttcc aaggcgaatt cgaagcttgg

601 ctgttttggc ggatgagaga agattttcag cctgatacag attaaatcag aacgcagaag

661 cggtctgata aaacagaatt tgcctggcgg cagtagcgcg gtggtcccac ctgaccccat

721 gccgaactca gaagtgaaac gccgtagcgc cgatggtagt gtggggtctc cccatgcgag

781 agtagggaac tgccaggcat caaataaaac gaaaggctca gtcgaaagac tgggcctttc

841 gttttatctg ttgtttgtcg gtgaacgctc tcctgagtag gacaaatccg ccgggagcgg

901 atttgaacgt tgcgaagcaa cggcccggag ggtggcgggc aggacgcccg ccataaactg

961 ccaggcatca aattaagcag aaggccatcc tgacggatgg cctttttgcg tttctacaaa

1021 ctcttttgtt tatttttcta aatacattca aatatgtatc cgctcatgag acaataaccc

1081 tgataaatgc ttcaataata ttgaaaaagg aagagtatga gtattcaaca tttccgtgtc

1141 gcccttattc ccttttttgc ggcattttgc cttcctgttt ttgctcaccc agaaacgctg

1201 gtgaaagtaa aagatgctga agatcagttg ggtgcacgag tgggttacat cgaactggat

1261 ctcaacagcg gtaagatcct tgagagtttt cgccccgaag aacgttttcc aatgatgagc

1321 acttttaaag ttctgctatg tggcgcggta ttatcccgtg ttgacgccgg gcaagagcaa

1381 ctcggtcgcc gcatacacta ttctcagaat gacttggttg agtactcacc agtcacagaa

1441 aagcatctta cggatggcat gacagtaaga gaattatgca gtgctgccat aaccatgagt

1501 gataacactg cggccaactt acttctgaca acgatcggag gaccgaagga gctaaccgct

1561 tttttgcaca acatggggga tcatgtaact cgccttgatc gttgggaacc ggagctgaat

1621 gaagccatac caaacgacga gcgtgacacc acgatgcctg tagcaatggc aacaacgttg

1681 cgcaaactat taactggcga actacttact ctagcttccc ggcaacaatt aatagactgg

1741 atggaggcgg ataaagttgc aggaccactt ctgcgctcgg cccttccggc tggctggttt

1801 attgctgata aatctggagc cggtgagcgt gggtctcgcg gtatcattgc agcactgggg

1861 ccagatggta agccctcccg tatcgtagtt atctacacga cggggagtca ggcaactatg

1921 gatgaacgaa atagacagat cgctgagata ggtgcctcac tgattaagca ttggtaactg

1981 tcagaccaag tttactcata tatactttag attgatttaa aacttcattt ttaatttaaa

2041 aggatctagg tgaagatcct ttttgataat ctcatgacca aaatccctta acgtgagttt

2101 tcgttccact gagcgtcaga ccccgtagaa aagatcaaag gatcttcttg agatcctttt

2161 tttctgcgcg taatctgctg cttgcaaaca aaaaaaccac cgctaccagc ggtggtttgt

2221 ttgccggatc aagagctacc aactcttttt ccgaaggtaa ctggcttcag cagagcgcag

2281 ataccaaata ctgtccttct agtgtagccg tagttaggcc accacttcaa gaactctgta

2341 gcaccgccta catacctcgc tctgctaatc ctgttaccag tggctgctgc cagtggcgat

2401 aagtcgtgtc ttaccgggtt ggactcaaga cgatagttac cggataaggc gcagcggtcg

2461 ggctgaacgg ggggttcgtg cacacagccc agcttggagc gaacgaccta caccgaactg

2521 agatacctac agcgtgagct atgagaaagc gccacgcttc ccgaagggag aaaggcggac

2581 aggtatccgg taagcggcag ggtcggaaca ggagagcgca cgagggagct tccaggggga

2641 aacgcctggt atctttatag tcctgtcggg tttcgccacc tctgacttga gcgtcgattt

2701 ttgtgatgct cgtcaggggg gcggagccta tggaaaaacg ccagcaacgc ggccttttta

2761 cggttcctgg ccttttgctg gccttttgct cacatgttct ttcctgcgtt atcccctgat

2821 tctgtggata accgtattac cgcctttgag tgagctgata ccgctcgccg cagccgaacg

2881 accgagcgca gcgagtcagt gagcgaggaa gcggaagagc gcctgatgcg gtattttctc

2941 cttacgcatc tgtgcggtat ttcacaccgc atatggtgca ctctcagtac aatctgctct

3001 gatgccgcat agttaagcca gtatacactc cgctatcgct acgtgactgg gtcatggctg

3061 cgccccgaca cccgccaaca cccgctgacg cgccctgacg ggcttgtctg ctcccggcat

3121 ccgcttacag acaagctgtg accgtctccg ggagctgcat gtgtcagagg ttttcaccgt

3181 catcaccgaa acgcgcgagg cagcagatca attcgcgcgc gaaggcgaag cggcatgcat

3241 ttacgttgac accatcgaat ggtgcaaaac ctttcgcggt atggcatgat agcgcccgga

3301 agagagtcaa ttcagggtgg tgaatgtgaa accagtaacg ttatacgatg tcgcagagta

3361 tgccggtgtc tcttatcaga ccgtttcccg cgtggtgaac caggccagcc acgtttctgc

3421 gaaaacgcgg gaaaaagtgg aagcggcgat ggcggagctg aattacattc ccaaccgcgt

3481 ggcacaacaa ctggcgggca aacagtcgtt gctgattggc gttgccacct ccagtctggc

3541 cctgcacgcg ccgtcgcaaa ttgtcgcggc gattaaatct cgcgccgatc aactgggtgc

3601 cagcgtggtg gtgtcgatgg tagaacgaag cggcgtcgaa gcctgtaaag cggcggtgca

3661 caatcttctc gcgcaacgcg tcagtgggct gatcattaac tatccgctgg atgaccagga

3721 tgccattgct gtggaagctg cctgcactaa tgttccggcg ttatttcttg atgtctctga

3781 ccagacaccc atcaacagta ttattttctc ccatgaagac ggtacgcgac tgggcgtgga

3841 gcatctggtc gcattgggtc accagcaaat cgcgctgtta gcgggcccat taagttctgt

3901 ctcggcgcgt ctgcgtctgg ctggctggca taaatatctc actcgcaatc aaattcagcc

3961 gatagcggaa cgggaaggcg actggagtgc catgtccggt tttcaacaaa ccatgcaaat

4021 gctgaatgag ggcatcgttc ccactgcgat gctggttgcc aacgatcaga tggcgctggg

4081 cgcaatgcgc gccattaccg agtccgggct gcgcgttggt gcggatatct cggtagtggg

4141 atacgacgat accgaagaca gctcatgtta tatcccgccg ttaaccacca tcaaacagga

4201 ttttcgcctg ctggggcaaa ccagcgtgga ccgcttgctg caactctctc agggccaggc

4261 ggtgaagggc aatcagctgt tgcccgtctc actggtgaaa agaaaaacca ccctggcgcc

4321 caatacgcaa accgcctctc cccgcgcgtt ggccgattca ttaatgcagc tggcacgaca

4381 ggtttcccga ctggaaagcg ggcagtgagc gcaacgcaat taatgtaagt tagcgcgaat

4441 tgatctg

//

**Figure I in S1 file. *Streptococcus thermophilus* forward orientation target (STH-f) plasmid.** STH crRNA target sequence is in the forward orientation (yellow, STH-f target), relative to the trc promoter (blue). The target sequence is inserted between the IPTG-inducible lac operator (teal) and rrnB T1/T2 terminators (red) of the pTrcHis-TOPO plasmid, which carries the colE1 origin of replication (colE1 ori, olive color) and ampicillin resistance marker (purple). Green arrows represent AmpR and lacIq transcriptional promoters. Plasmid map illustration made using the SnapGene program. Plasmid sequence information is in GenBank format, using ApE program.


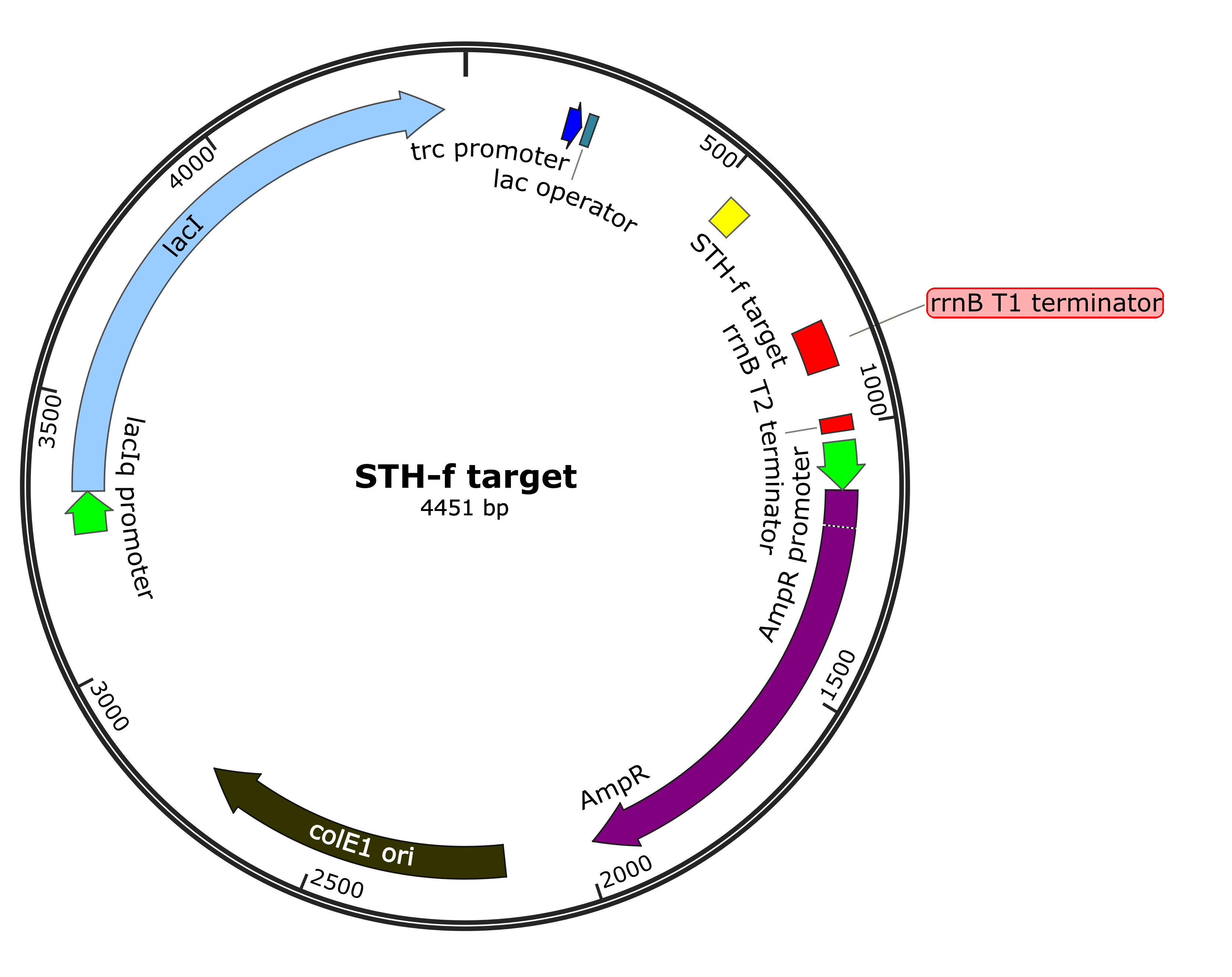


LOCUS STH_f_target 4451 bp ds-DNA circular 19-FEB-2017

DEFINITION synthetic circular DNA

ACCESSION .

VERSION .

KEYWORDS STH-f target

SOURCE synthetic DNA construct

ORGANISM synthetic DNA construct

REFERENCE 1 (bases 1 to 4451)

AUTHORS .

TITLE Direct Submission

JOURNAL Exported Sunday, Feb 19, 2017 from SnapGene 3.3.3

http://www.snapgene.com

COMMENT

COMMENT ApEinfo:methylated:1

FEATURES Location/Qualifiers

promoter 193..222

/note="trc promoter"

/note="strong E. coli promoter; hybrid between the trp and

lac UV5 promoters"

/label=trc promoter

/ApEinfo_fwdcolor=#0000ff

/ApEinfo_revcolor=#0000ff

/ApEinfo_graphicformat=arrow_data {{0 1 2 0 0 -1} {} 0}

width 5 offset 0

protein_bind 230..246

/bound_moiety="lac repressor encoded by lacI"

/note="lac operator"

/note="The lac repressor binds to the lac operator to

inhibit transcription in E. coli. This inhibition can be

relieved by adding lactose or

isopropyl-beta-D-thiogalactopyranoside (IPTG)."

/label=lac operator

/ApEinfo_fwdcolor=#008080

/ApEinfo_revcolor=#008080

/ApEinfo_graphicformat=arrow_data {{0 1 2 0 0 -1} {} 0}

width 5 offset 0

misc_feature 528..574

/note="STH-f target"

/label=STH-f target

/ApEinfo_fwdcolor=#ffff00

/ApEinfo_revcolor=#ffff00

/ApEinfo_graphicformat=arrow_data {{0 1 2 0 0 -1} {} 0}

width 5 offset 0

terminator 805..891

/gene="Escherichia coli rrnB"

/note="rrnB T1 terminator"

/note="transcription terminator T1 from the E. coli rrnB

gene"

/label=rrnB T1 terminator

/ApEinfo_fwdcolor=#ff0000

/ApEinfo_revcolor=#ff0000

/ApEinfo_graphicformat=arrow_data {{0 1 2 0 0 -1} {} 0}

width 5 offset 0

terminator 983..1010

/note="rrnB T2 terminator"

/note="transcription terminator T2 from the E. coli rrnB

gene"

/label=rrnB T2 terminator

/ApEinfo_fwdcolor=#ff0000

/ApEinfo_revcolor=#ff0000

/ApEinfo_graphicformat=arrow_data {{0 1 2 0 0 -1} {} 0}

width 5 offset 0

promoter 1029..1120

/gene="bla"

/note="AmpR promoter"

/label=AmpR promoter

/ApEinfo_fwdcolor=#00ff00

/ApEinfo_revcolor=#00ff00

/ApEinfo_graphicformat=arrow_data {{0 1 2 0 0 -1} {} 0}

width 5 offset 0

CDS 1121..1981

/codon_start=1

/gene="bla"

/product="beta-lactamase"

/note="AmpR"

/note="confers resistance to ampicillin, carbenicillin,

and related antibiotics"

/translation="MSIQHFRVALIPFFAAFCLPVFAHPETLVKVKDAEDQLGARVGYIELDLNSGKILESFRPEERFPMMSTFKVLLCGAVLSRVDAGQEQLGRRIHYSQNDLVEYSPVTEKHLTDGMTVRELCSAAITMSDNTAANLLLTTIGGPKELTAFLHNMGDHVTRLDRWEPELNEAIPNDERDTTMPVAMATTLRKLLTGELLTLASRQQLIDWMEADKVAGPLLRSALPAGWFIADKSGAGERGSRGIIAALGPDGKPSRIVVIYTTGSQATMDERNRQIAEIGASLIKHW"

/label=AmpR

/ApEinfo_fwdcolor=#800080

/ApEinfo_revcolor=#800080

/ApEinfo_graphicformat=arrow_data {{0 1 2 0 0 -1} {} 0}

width 5 offset 0

rep_origin 2152..2740

/direction=RIGHT

/note="colE1 ori"

/note="high-copy-number ColE1/pMB1/pBR322/pUC origin of

replication"

/label=colE1 ori

/ApEinfo_fwdcolor=#808000

/ApEinfo_revcolor=#808000

/ApEinfo_graphicformat=arrow_data {{0 1 2 0 0 -1} {} 0}

width 5 offset 0

promoter 3252..3329

/gene="lacI (mutant)"

/note="lacIq promoter"

/note="In the lacIq allele, a single base change in the

promoter boosts expression of the lacI gene about

10-fold."

/label=lacIq promoter

/ApEinfo_fwdcolor=#00ff00

/ApEinfo_revcolor=#00ff00

/ApEinfo_graphicformat=arrow_data {{0 1 2 0 0 -1} {} 0}

width 5 offset 0

CDS 3330..4412

/codon_start=1

/gene="lacI"

/product="lac repressor"

/note="lacI"

/note="The lac repressor binds to the lac operator to

inhibit transcription in E. coli. This inhibition can be

relieved by adding lactose or

isopropyl-beta-D-thiogalactopyranoside (IPTG)."

/translation="MKPVTLYDVAEYAGVSYQTVSRVVNQASHVSAKTREKVEAAMAELNYIPNRVAQQLAGKQSLLIGVATSSLALHAPSQIVAAIKSRADQLGASVVVSMVERSGVEACKAAVHNLLAQRVSGLIINYPLDDQDAIAVEAACTNVPALFLDVSDQTPINSIIFSHEDGTRLGVEHLVALGHQQIALLAGPLSSVSARLRLAGWHKYLTRNQIQPIAEREGDWSAMSGFQQTMQMLNEGIVPTAMLVANDQMALGAMRAITESGLRVGADISVVGYDDTEDSSCYIPPLTTIKQDFRLLGQTSVDRLLQLSQGQAVKGNQLLPVSLVKRKTTLAPNTQTASPRALADSLMQLARQVSRLESGQ"

/label=lacI

/ApEinfo_fwdcolor=#66ccff

/ApEinfo_revcolor=#66ccff

/ApEinfo_graphicformat=arrow_data {{0 1 2 0 0 -1} {} 0}

width 5 offset 0

ORIGIN

1 gtttgacagc ttatcatcga ctgcacggtg caccaatgct tctggcgtca ggcagccatc

61 ggaagctgtg gtatggctgt gcaggtcgta aatcactgca taattcgtgt cgctcaaggc

121 gcactcccgt tctggataat gttttttgcg ccgacatcat aacggttctg gcaaatattc

181 tgaaatgagc tgttgacaat taatcatccg gctcgtataa tgtgtggaat tgtgagcgga

241 taacaatttc acacaggaaa cagcgccgct gagaaaaagc gaagcggcac tgctctttaa

301 caatttatca gacaatctgt gtgggcactc gaccggaatt atcgattaac tttattatta

361 aaaattaaag aggtatatat taatgtatcg attaaataag gaggaataaa ccatgggggg

421 ttctcatcat catcatcatc atggtatggc tagcatgact ggtggacagc aaatgggtcg

481 ggatctgtac gacgatgacg ataaggatcc aacccttttc caagcttcat tgctaacgct

541 tatttagaag tagcgttaga atcaagcaaa ggcagcatgc ttccaaggcg aattcgaagc

601 ttggctgttt tggcggatga gagaagattt tcagcctgat acagattaaa tcagaacgca

661 gaagcggtct gataaaacag aatttgcctg gcggcagtag cgcggtggtc ccacctgacc

721 ccatgccgaa ctcagaagtg aaacgccgta gcgccgatgg tagtgtgggg tctccccatg

781 cgagagtagg gaactgccag gcatcaaata aaacgaaagg ctcagtcgaa agactgggcc

841 tttcgtttta tctgttgttt gtcggtgaac gctctcctga gtaggacaaa tccgccggga

901 gcggatttga acgttgcgaa gcaacggccc ggagggtggc gggcaggacg cccgccataa

961 actgccaggc atcaaattaa gcagaaggcc atcctgacgg atggcctttt tgcgtttcta

1021 caaactcttt tgtttatttt tctaaataca ttcaaatatg tatccgctca tgagacaata

1081 accctgataa atgcttcaat aatattgaaa aaggaagagt atgagtattc aacatttccg

1141 tgtcgccctt attccctttt ttgcggcatt ttgccttcct gtttttgctc acccagaaac

1201 gctggtgaaa gtaaaagatg ctgaagatca gttgggtgca cgagtgggtt acatcgaact

1261 ggatctcaac agcggtaaga tccttgagag ttttcgcccc gaagaacgtt ttccaatgat

1321 gagcactttt aaagttctgc tatgtggcgc ggtattatcc cgtgttgacg ccgggcaaga

1381 gcaactcggt cgccgcatac actattctca gaatgacttg gttgagtact caccagtcac

1441 agaaaagcat cttacggatg gcatgacagt aagagaatta tgcagtgctg ccataaccat

1501 gagtgataac actgcggcca acttacttct gacaacgatc ggaggaccga aggagctaac

1561 cgcttttttg cacaacatgg gggatcatgt aactcgcctt gatcgttggg aaccggagct

1621 gaatgaagcc ataccaaacg acgagcgtga caccacgatg cctgtagcaa tggcaacaac

1681 gttgcgcaaa ctattaactg gcgaactact tactctagct tcccggcaac aattaataga

1741 ctggatggag gcggataaag ttgcaggacc acttctgcgc tcggcccttc cggctggctg

1801 gtttattgct gataaatctg gagccggtga gcgtgggtct cgcggtatca ttgcagcact

1861 ggggccagat ggtaagccct cccgtatcgt agttatctac acgacgggga gtcaggcaac

1921 tatggatgaa cgaaatagac agatcgctga gataggtgcc tcactgatta agcattggta

1981 actgtcagac caagtttact catatatact ttagattgat ttaaaacttc atttttaatt

2041 taaaaggatc taggtgaaga tcctttttga taatctcatg accaaaatcc cttaacgtga

2101 gttttcgttc cactgagcgt cagaccccgt agaaaagatc aaaggatctt cttgagatcc

2161 tttttttctg cgcgtaatct gctgcttgca aacaaaaaaa ccaccgctac cagcggtggt

2221 ttgtttgccg gatcaagagc taccaactct ttttccgaag gtaactggct tcagcagagc

2281 gcagatacca aatactgtcc ttctagtgta gccgtagtta ggccaccact tcaagaactc

2341 tgtagcaccg cctacatacc tcgctctgct aatcctgtta ccagtggctg ctgccagtgg

2401 cgataagtcg tgtcttaccg ggttggactc aagacgatag ttaccggata aggcgcagcg

2461 gtcgggctga acggggggtt cgtgcacaca gcccagcttg gagcgaacga cctacaccga

2521 actgagatac ctacagcgtg agctatgaga aagcgccacg cttcccgaag ggagaaaggc

2581 ggacaggtat ccggtaagcg gcagggtcgg aacaggagag cgcacgaggg agcttccagg

2641 gggaaacgcc tggtatcttt atagtcctgt cgggtttcgc cacctctgac ttgagcgtcg

2701 atttttgtga tgctcgtcag gggggcggag cctatggaaa aacgccagca acgcggcctt

2761 tttacggttc ctggcctttt gctggccttt tgctcacatg ttctttcctg cgttatcccc

2821 tgattctgtg gataaccgta ttaccgcctt tgagtgagct gataccgctc gccgcagccg

2881 aacgaccgag cgcagcgagt cagtgagcga ggaagcggaa gagcgcctga tgcggtattt

2941 tctccttacg catctgtgcg gtatttcaca ccgcatatgg tgcactctca gtacaatctg

3001 ctctgatgcc gcatagttaa gccagtatac actccgctat cgctacgtga ctgggtcatg

3061 gctgcgcccc gacacccgcc aacacccgct gacgcgccct gacgggcttg tctgctcccg

3121 gcatccgctt acagacaagc tgtgaccgtc tccgggagct gcatgtgtca gaggttttca

3181 ccgtcatcac cgaaacgcgc gaggcagcag atcaattcgc gcgcgaaggc gaagcggcat

3241 gcatttacgt tgacaccatc gaatggtgca aaacctttcg cggtatggca tgatagcgcc

3301 cggaagagag tcaattcagg gtggtgaatg tgaaaccagt aacgttatac gatgtcgcag

3361 agtatgccgg tgtctcttat cagaccgttt cccgcgtggt gaaccaggcc agccacgttt

3421 ctgcgaaaac gcgggaaaaa gtggaagcgg cgatggcgga gctgaattac attcccaacc

3481 gcgtggcaca acaactggcg ggcaaacagt cgttgctgat tggcgttgcc acctccagtc

3541 tggccctgca cgcgccgtcg caaattgtcg cggcgattaa atctcgcgcc gatcaactgg

3601 gtgccagcgt ggtggtgtcg atggtagaac gaagcggcgt cgaagcctgt aaagcggcgg

3661 tgcacaatct tctcgcgcaa cgcgtcagtg ggctgatcat taactatccg ctggatgacc

3721 aggatgccat tgctgtggaa gctgcctgca ctaatgttcc ggcgttattt cttgatgtct

3781 ctgaccagac acccatcaac agtattattt tctcccatga agacggtacg cgactgggcg

3841 tggagcatct ggtcgcattg ggtcaccagc aaatcgcgct gttagcgggc ccattaagtt

3901 ctgtctcggc gcgtctgcgt ctggctggct ggcataaata tctcactcgc aatcaaattc

3961 agccgatagc ggaacgggaa ggcgactgga gtgccatgtc cggttttcaa caaaccatgc

4021 aaatgctgaa tgagggcatc gttcccactg cgatgctggt tgccaacgat cagatggcgc

4081 tgggcgcaat gcgcgccatt accgagtccg ggctgcgcgt tggtgcggat atctcggtag

4141 tgggatacga cgataccgaa gacagctcat gttatatccc gccgttaacc accatcaaac

4201 aggattttcg cctgctgggg caaaccagcg tggaccgctt gctgcaactc tctcagggcc

4261 aggcggtgaa gggcaatcag ctgttgcccg tctcactggt gaaaagaaaa accaccctgg

4321 cgcccaatac gcaaaccgcc tctccccgcg cgttggccga ttcattaatg cagctggcac

4381 gacaggtttc ccgactggaa agcgggcagt gagcgcaacg caattaatgt aagttagcgc

4441 gaattgatct g

//
